# Efficacy and safety of traditional Chinese medicine decoction as an adjuvant treatment for diabetic nephropathy: a systematic review and meta-analysis of randomized controlled trials

**DOI:** 10.3389/fphar.2024.1327030

**Published:** 2024-05-09

**Authors:** Shuyu Zheng, Yunxi Xu, Ya Zhang, Caiyi Long, Guo Chen, Zhao Jin, Shui Jiang, Junyu Chen, Yulian Qin

**Affiliations:** ^1^ Department of Clinical Medicine, Chengdu University of Traditional Chinese Medicine, Chengdu, China; ^2^ Department of Endocrinology, Affiliated Hospital of Chengdu University of Traditional Chinese Medicine, Chengdu, China; ^3^ Department of Infectious Diseases, Affiliated Hospital of Chengdu University of Traditional Chinese Medicine, Chengdu, China; ^4^ Chengdu University of Traditional Chinese Medicine, Chengdu, China; ^5^ Good Clinical Practice Department, Chengdu Jingdongfang Hospital, Chengdu, China

**Keywords:** adjuvant therapy, traditional Chinese medicine, Chinese medicine decoction, diabetic nephropathy, meta-analysis

## Abstract

**Objective:** This study aimed to assess the efficacy and safety of traditional Chinese medicine decoction as an adjunctive treatment for diabetic nephropathy in systematic evaluations.

**Methods:** A comprehensive search was conducted in PubMed, Web of Science, Cochrane Library, Embase, China National Knowledge Infrastructure (CNKI), and Wanfang databases, covering the period from January 2013 to July 2023. The search was restricted to randomized controlled trials (RCTs) conducted within the past decade that investigated the use of TCM decoction as an adjunctive treatment for diabetic nephropathy. The control group received western medicine treatment, while the intervention group received TCM decoction in addition to the conventional treatment. Endnote and Excel were employed for literature management and data organization, and Revman 5.3 and Stata 16 software were used for the analyses.

**Results:** 66 RCTs involving 6,951 participants were included in this study. The clinical efficacy of TCM decoction as an adjunctive treatment for diabetic nephropathy was found to be significantly higher than that of the control group (OR = 3.12, 95% CI [2.70, 3.60], I^2^ = 0%, *p* < 0.00001). The incidence of adverse events did not differ significantly between the intervention group and the control group (OR = 0.94, 95% CI [0.60, 1.48], I^2^ = 0%, *p* = 0.94). According to the secondary outcomes of renal function and blood glucose indicators, the intervention group showed better therapeutic efficacy compared to the control group. The most frequently used TCM categories were tonifying medicine, blood-activating medicine, astringent medicine, diuretic medicine, heat-clearing medicine, and laxative medicine. Among them, the top five frequently used Chinese medicine were *Astragalus mongholicus Bunge* [Fabaceae; Astragali mongholici radix](58 times), *Salvia miltiorrhiza Bunge* [Lamiaceae; Radix et rhizoma salviae miltiorrhizae] (42 times), *Dioscorea oppositifolia L.* [Dioscoreaceae; Dioscoreae rhizoma] (38 times), *Poria cocos (Schw.) Wolf* [Polyporaceae; Poria] (38 times), and *Cornus officinalis Siebold & Zucc.* [Cornaceae; Corni fructus] (35 times).

**Conclusion:** The combined use of TCM decoction with western medicine in the treatment of diabetic nephropathy can enhance clinical effectiveness and 2 This is a provisional file, not the final typeset article achieve superior therapeutic effects in comparison to western medicine alone, without significant risks.

**Systematic Review Registration:** https://www.crd.york.ac.uk/PROSPERO/#recordDetails, identifier [CRD42022529144].

## 1 Introduction

Diabetes Mellitus (DM) is a metabolic disorder caused by a combination of genetic, autoimmune, and environmental factors. In China, the total number of individuals with diabetes is approximately 120 million (International Diabetes Federation, 2023). It is estimated that 20%–40% of diabetes patients in China also suffer from diabetic nephropathy, which is the leading cause of end-stage renal disease (ESRD) and a major cause of mortality among diabetes patients (Barrera-Chimal et al., 2022; Naaman and Bakris, 2023).

Traditional Chinese medicine (TCM) decoction is commonly used as an adjunctive therapy for treating diabetic nephropathy in China. It has been shown to improve patient symptoms and slow down the progression of diabetic nephropathy, with good clinical efficacy and safety. Liu et al. (Liu et al., 2022) found that both Huang Kui Capsules and Zi Cui Yin Decoction were able to reduce Scr levels and improve symptom scores in patients. Furthermore, Zi Cui Yin Decoction was found to have a positive impact on correcting gut microbiota dysbiosis. Another study by Sun et al. (Sun et al., 2022) revealed that Danggui Buxue decoction improved insulin resistance, chronic inflammation, and lipid accumulation in diabetic nephropathy mice. Additionally, Wang et al. (Wang et al., 2018) discovered that Shenqi Dihuang decoction significantly intervened in the inflammatory response, reduced proteinuria, protected renal function, improved endothelial function and hemorheology, restored microcirculation to normal levels, or demonstrated good therapeutic efficacy in early-stage diabetic nephropathy patients.

Currently, most studies have been limited to small-sample clinical trials conducted at a single center or non-clinical trials. There is a wide variety of TCM decoctions used, considerable differences in outcome measures, or a low level of evidence. Therefore, there is a need for a systematic analysis and evaluation of the effectiveness and safety of TCM decoction as an adjunctive treatment for diabetic nephropathy.

## 2 Materials and methods

### 2.1 Protocol and registration

The meta-analysis was registered with the International Prospective Register of Systematic Reviews (PROSPERO) under the registration number CRD42022529144. We followed the Preferred Reporting Items for Systematic Reviews and Meta-Analyses (PRISMA), its protocols, and the PRISMA-extension statementfor meta-analysis to report the current results (Hutton et al., 2015).

### 2.2 Search methods

A systematic search of the PubMed, Web of Science, Cochrane Library, Embase, China National Knowledge Infrastructure (CNKI), and Wanfang databases for randomized controlled trials (RCTs) was conducted from 2013 to the present that investigated the use of traditional Chinese medicine decoction as an adjunctive treatment for diabetic nephropathy (DN). The Chinese search terms included “diabetic nephropathy& Xiao ke nephropathy” and “traditional Chinese medicine& Chinese botanical medicine& Chinese medicine decoction” and “randomized controlled trailsand clinical trials”. The English search terms included “diabetic kidney diseaseand diabetic nephropathyand diabetic” and “Chinese Medicine & Chinese Botanical Medicine & Traditional Chinese Medicine & Chinese Medicine decoction” and “Randomized Controlled Trial & Clinical Trial & Intervention Study & Clinical Study”. There were no language or geographic restrictions. In addition, we manually supplemented the included studies by examining the references of the identified articles that met the inclusion criteria. The search strategies are provided in Supplementary Table S1.

### 2.3 Data extraction

After screening and assessing the identified literature by reviewing titles, abstracts, and full texts, eligible RCTs were included based on the following inclusion and exclusion criteria.

Inclusion criteria:

(a) Participants: There were no gender or race restrictions. Adult individuals (aged 18 years and above) diagnosed with DN based on clinical assessment were included.
(b) Intervention and comparison: The control group received Western medicine treatment, while the intervention group received traditional Chinese medicine decoction in addition to the control group’s treatment.
(c) The study should report at least one of the following outcomes: primary outcome - clinical effective rate, adverse event rate. Secondary outcomes - renal function indicators: serum creatinine (Scr), urinary albumin excretion rate (UAER), blood urea nitrogen (BUN), 24-h urinary total protein (24 h-utp) and blood glucose indicators: fasting plasma glucose (FPG), glycated hemoglobin (HbA1c).

Exclusion criteria:

(a) Non-randomized controlled trials, retrospective studies, animal experiments, and review articles;
(b) Participants: Exclusion of individuals with renal dysfunction caused by other reasons.
(c) Intervention: Intervention group using other traditional Chinese medicine treatments, such as Chinese patent medicine, Chinese medicine pills, Chinese medicine injections, acupuncture, massage, or ear acupuncture.
(d) Outcomes: Data not accurate or outcome measurement incomplete, and unable to obtain data from the original authors.
(e) Duplicate publications.

### 2.4 Study selection

Two researchers (Shuyu Zheng and Yunxi Xu) independently conducted data collection using the aforementioned search methods and inclusion criteria. The following information was extracted:

(a) Publication information (title, first author, publication year).
(b) Study characteristics (study design, treatment duration).
(c) Participant characteristics (number of participants included, age, gender, duration of diabetes mellitus (DM), stage of diabetic nephropathy (DN)).
(d) Intervention (intervention drugs, dosage, frequency, route of administration for the control group; composition of traditional Chinese medicine decoctions, dosage, frequency of administration for the intervention group, based on the control group).
(e) Outcomes (primary and secondary outcomes). For continuous data, mean and standard deviation were extracted. For categorical data, the number of events and total count were extracted.

In cases of disagreement between the two researchers, a third researcher (Caiyi Long) made the final judgment.

### 2.5 Critical appraisal

Using the bias risk assessment tool provided by Cochrane, two researchers (Junyu Chen and Yulian Qin) independently assessed the quality of the included RCT studies and cross-checked their assessments. The assessment consisted of seven components: random sequence generation, allocation concealment, blinding of participants and personnel, completeness of outcome data, reporting bias, and other biases. Three categories were used to assess the methodological quality of the studies: “high risk of bias,” “low risk of bias,” and “unclear bias.” In cases of disagreement between the two researchers, a third researcher (Shui Jiang) made the final judgment.

### 2.6 Statistical analysis

EndNote 9.0 was used for literature management, Excel was used for data organization, and RevMan 5.3 and Stata 16.0 were employed for statistical analysis.

For binary outcome variables, the effect measure statistic used was odds ratio (OR), while for continuous outcome variables, the effect measure statistic used was mean difference (MD) or standardized mean difference (SMD). A 95% confidence interval (CI) and *I* ^*2*^ test were used to assess heterogeneity. If *p* ≥ 0.05 and *I* ^*2*^ < 50%, a fixed-effect model was used for analysis. If *p* < 0.05 and *I* ^*2*^ ≥ 50%, a random-effects model was used for analysis. A significance level of α = 0.05 was used for meta-analysis. Sensitivity analysis was conducted using the one-by-one elimination method. Publication bias was assessed using funnel plots and Egger’s test.

## 3 Results

### 3.1 Research and selection

**FIGURE 1:**
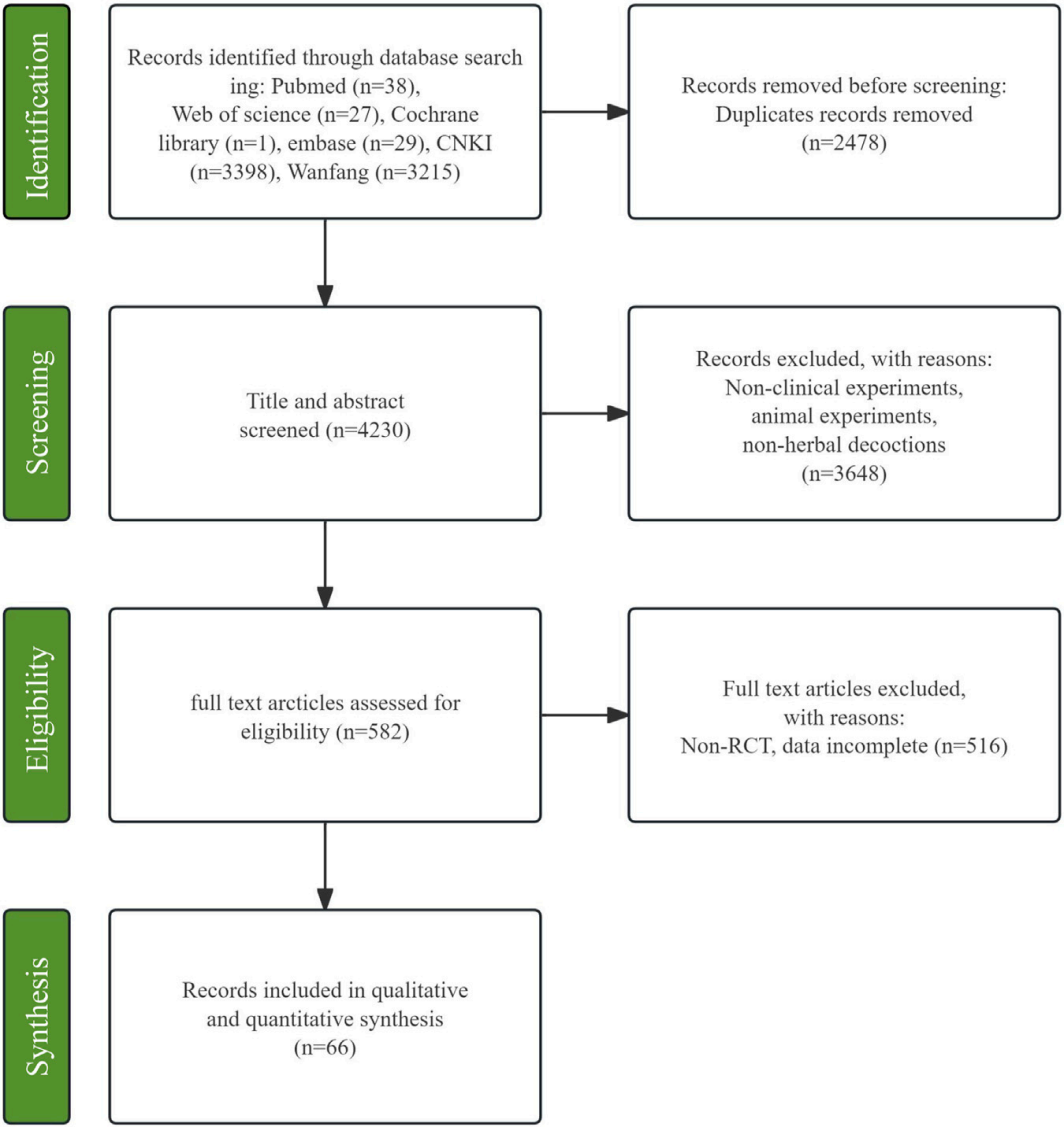
Flow plot.

After database searching, a total of 6,706 relevant studies were obtained. After removing 2,476 duplicate records, 4,230 studies were screened based on their titles and abstracts, resulting in the exclusion of 3,648 studies. Among the remaining 582 studies, full-text reading was conducted, and finally, 66 studies were included (Yi and Mianzhi, 2013; Angui, 2014; Liping et al., 2014; Yalian, 2014; Bailong et al., 2015; Beide and Tingting, 2015; Jialing and Yan, 2015; Jianmin, 2015; Jingjing et al., 2015; Ming et al., 2015; Ting et al., 2015; Lige, 2016; Shulan et al., 2016; Tao et al., 2016; Xiaojing et al., 2016; Ni et al., 2017; Ping and Lin, 2017; Rucui et al., 2017; Xiaoli et al., 2017; Aimin et al., 2018; Huajun et al., 2018; Liwen et al., 2018; Min et al., 2018; Shijian and Chunying, 2018; Xiaoyi, 2018; Xingguo et al., 2018; Changsong et al., 2019; Chenhui et al., 2019; Fangqiang et al., 2019; Gangyi et al., 2019; Jili and Hengji, 2019; Ling, 2019; Rong and Xiabo, 2019; Suqin et al., 2019; Xiaoze et al., 2019; Yangxi et al., 2019; Yangxia et al., 2019; Zhixiong et al., 2019; Dandan, 2020; Wei et al., 2020; Xinxin et al., 2020; Yalan et al., 2020; Bing and Guobin, 2021; Sun et al., 2022; An et al., 2022; Chuanfu et al., 2021; Chuanyong et al., 2022; Cuiqing et al., 2021; Duanyang et al., 2023; Haitao et al., 2022; Hongye et al., 2021; Huaizhi et al., 2021; Jiali and Dong, 2023; Jiang and Fang, 2021; Jinfeng et al., 2021; Lei et al., 2022; Li et al., 2022; Meizhen et al., 2022; Ping et al., 2022; Ruixuan et al., 2023; Xiaomei et al., 2021; Xin et al., 2023; Xue et al., 2023; Yali et al., 2020; Yan et al., 2022; Ying et al., 2021; Yu et al., 2022). Details of the selection process are presented in Figure 1.

### 3.2 Study characteristics

These 66 included studies involved a total of 6,915 patients, with 3,487 in the intervention group and 3,470 in the control group. The sample size of both the intervention and control groups was greater than 30, and all patients were from China. The average age of the intervention group ranged from 42.76 to 70.03, while the average age of the control group ranged from 45.37 to 69.21. The treatment duration ranged from 4 to 52 weeks. The included patients had DN stages ranging from III to IV. Interventions in the control group included conventional treatment, ACEI/ARB drugs, hypoglycemic medications, ipratropium bromide, prostaglandins, glutathione, and alfacalcidol capsules. The intervention group received traditional Chinese medicine decoctions in addition to the interventions used in the control group. The traditional Chinese medicine decoctions were classified into various groups based on their compositions. The criteria for classifying groups of Chinese medicines is the *Clinical Chinese Medicine* (Zhang et al., 2004), which delineate 12 groups including the Guizhi Fuling formula (GF) group, Yiqi Zishen formula (YZ) group, Shenqi Dihuang formula (SD) group, Yiqi Huayu formula (YH) group, Jianshen Huayu formula (JH) group, Buyuan Tongluo formula (BT) group, Wenshen Jianpi Huayu Tongluo formula (WJHT) group, Yiqi Yangyin Tongluo formula (YYT) group, Gushen Jianpi (GJ) group, Jiangtang Baoshen formula (JB) group, Bushen Yiqi Tongluo Huazhuo (BYTH) group and Huoxue Yishen formula (HY) group. A total of 105 different Chinese medicine from 14 categories were used, with the top five most frequently used Chinese medicine being *Astragalus mongholicus Bunge* [Fabaceae; Astragali mongholici radix], *Salvia miltiorrhiza Bunge* [Lamiaceae; Radix et rhizoma salviae miltiorrhizae], *Dioscorea oppositifolia L.* [Dioscoreaceae; Dioscoreae rhizoma], *Poria cocos (Schw.) Wolf* [Polyporaceae; Poria], and *Cornus officinalis Siebold & Zucc.* [Cornaceae; Corni fructus]. The studies’ characteristics, criteria for assessing clinical effectiveness rates, interventions, outcomes and the names of Chinese medicine decoction prescription are provided in Supplementary Tables S3–S8. All the Chinese medicine listed in the prescriptions have been categorized based on the standards set forth by *Clinical Herbal Medicine*. All Chinese medicines appearing in the prescription have been taxonomically validated in Kew Science resources and the *Pharmacopoeia of the People’s Republic of China*, 2020 edition (National Pharmacopoeia Committee, 2020; Trustees of the Royal Botanic Gardens and Kew, 2023). All the formula names were written in Chinese Pinyin and a table of standard terminologies for Chinese Pinyin is provided in Supplementary Table S7. For the scientific methodology of the writing, we consulted the guidelines by Rivera, D., et al. Please refer to Supplementary Table S8 for details (Rivera et al., 2014). We have adhered to the guidelines for reporting the composition, including preparation and included two PDFs of the assessment using the ConPhYMO tool. Please refer to the Appendix on page 89–90 for further information (Heinrich and Jalil, 2023).

### 3.3 Bias risk assessment results

**FIGURE 2:**
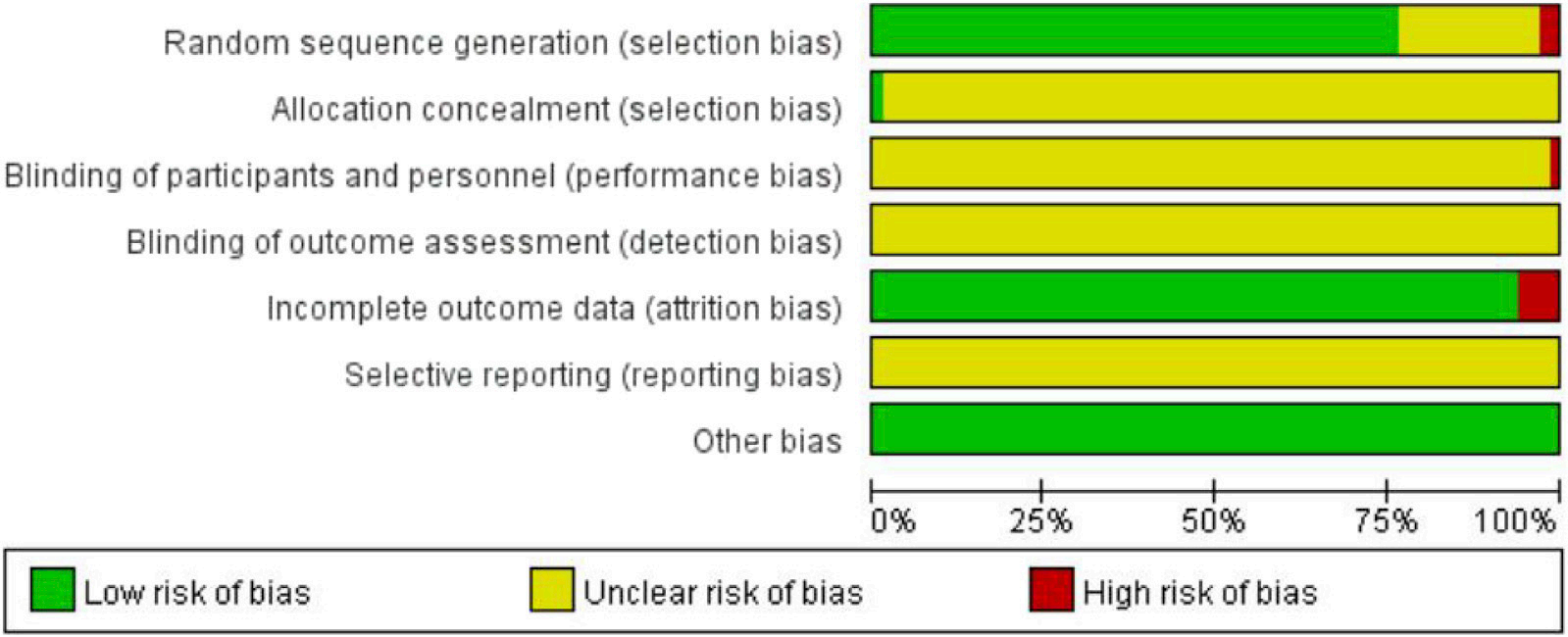
Risk of bias graph.

The quality of the included literature was evaluated using the Cochrane risk assessment tool. Among the 66 included studies, 50 studies used random number table allocation, and one study used dice rolling for random allocation (Jinfeng S, 2021), which were assessed as low risk. Two studies allocated participants based on the order of their visits (Rong Y, 2019; Suqin W, 2019), and were assessed as high risk. The remaining studies did not specify the specific method of allocation and were assessed as unknown risk. One study used the envelope method for allocation concealment (Yalian H, 2014), which was assessed as low risk. The remaining studies did not mention allocation concealment and were assessed as unknown risk. One study mentioned single-blinding and was assessed as high risk, while the remaining studies did not mention blinding and were assessed as unknown risk. Four studies had dropouts with corresponding explanations, and were assessed as high risk. The remaining studies had complete outcome data and were assessed as low risk. All studies were not registered and selective reporting could not be judged and was evaluated as unknown risk. No studies were found to have other biases and were assessed as low risk (Figure 2).

### 3.4 Primary outcomes

**FIGURE 3:**
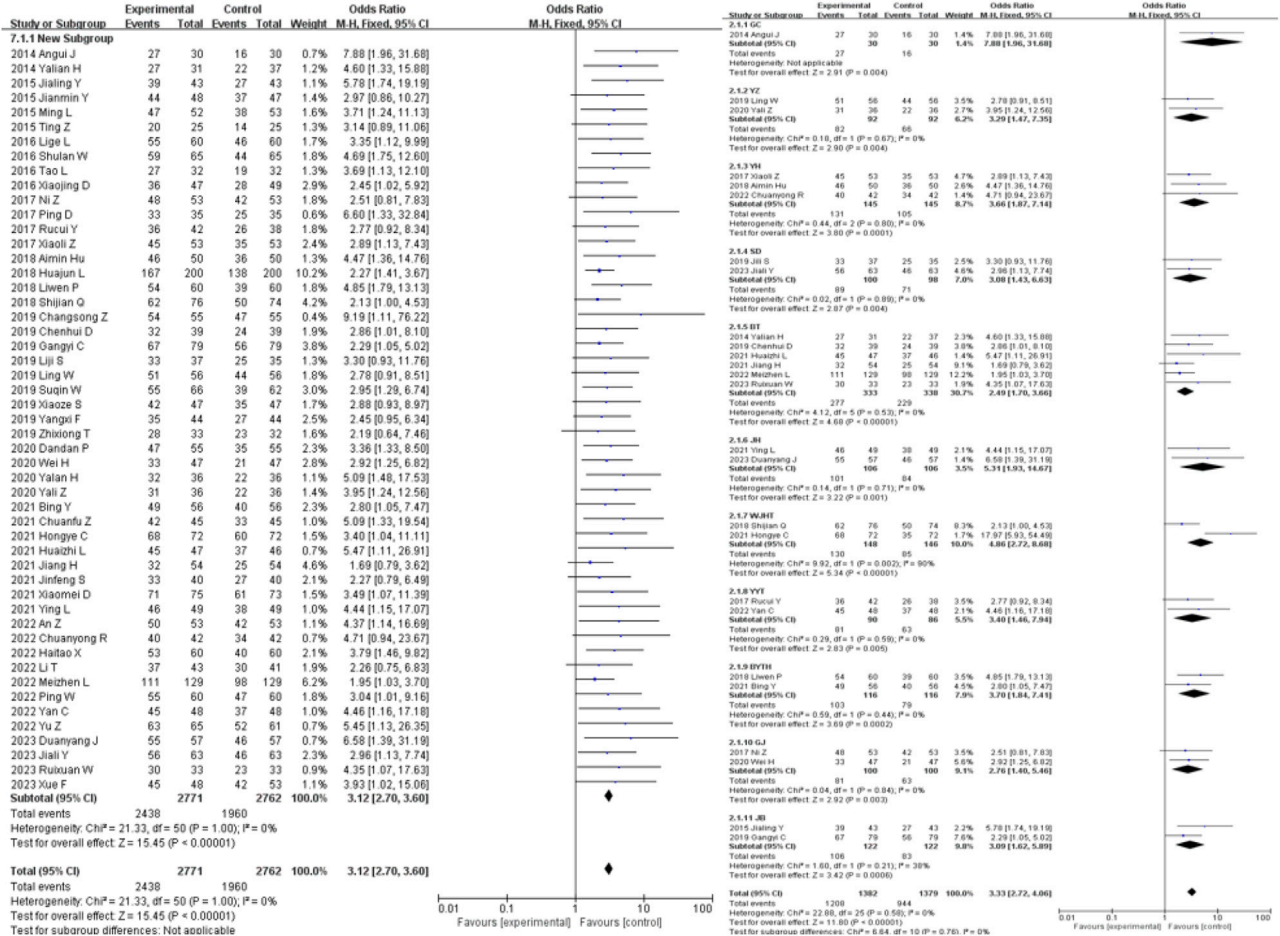
Efficacy forest plot & efficacy prescription forest plot.

51 studies were included in the analysis of the clinical efficacy outcome measure. The results showed that the clinical efficacy of traditional Chinese medicine decoctions as an adjunctive treatment for diabetic nephropathy was higher than that of the control group (OR = 3.12, 95% CI [2.70, 3.60], *I* ^*2*^ = 0%, *p* < 0.00001). Subgroup analysis based on the composition of the traditional Chinese medicine decoctions showed that the GF group had the most significant improvement in clinical efficacy compared to the control group (OR = 7.88, 95% CI [1.96, 31.68], *I* ^*2*^ = 0%, *p* = 0.004), while the WJHT group had the least significant improvement (OR = 2.46, 95% CI [1.31, 4.64], *I* ^*2*^ = 0%, *p* = 0.005) (Figure 3).

**FIGURE 4:**
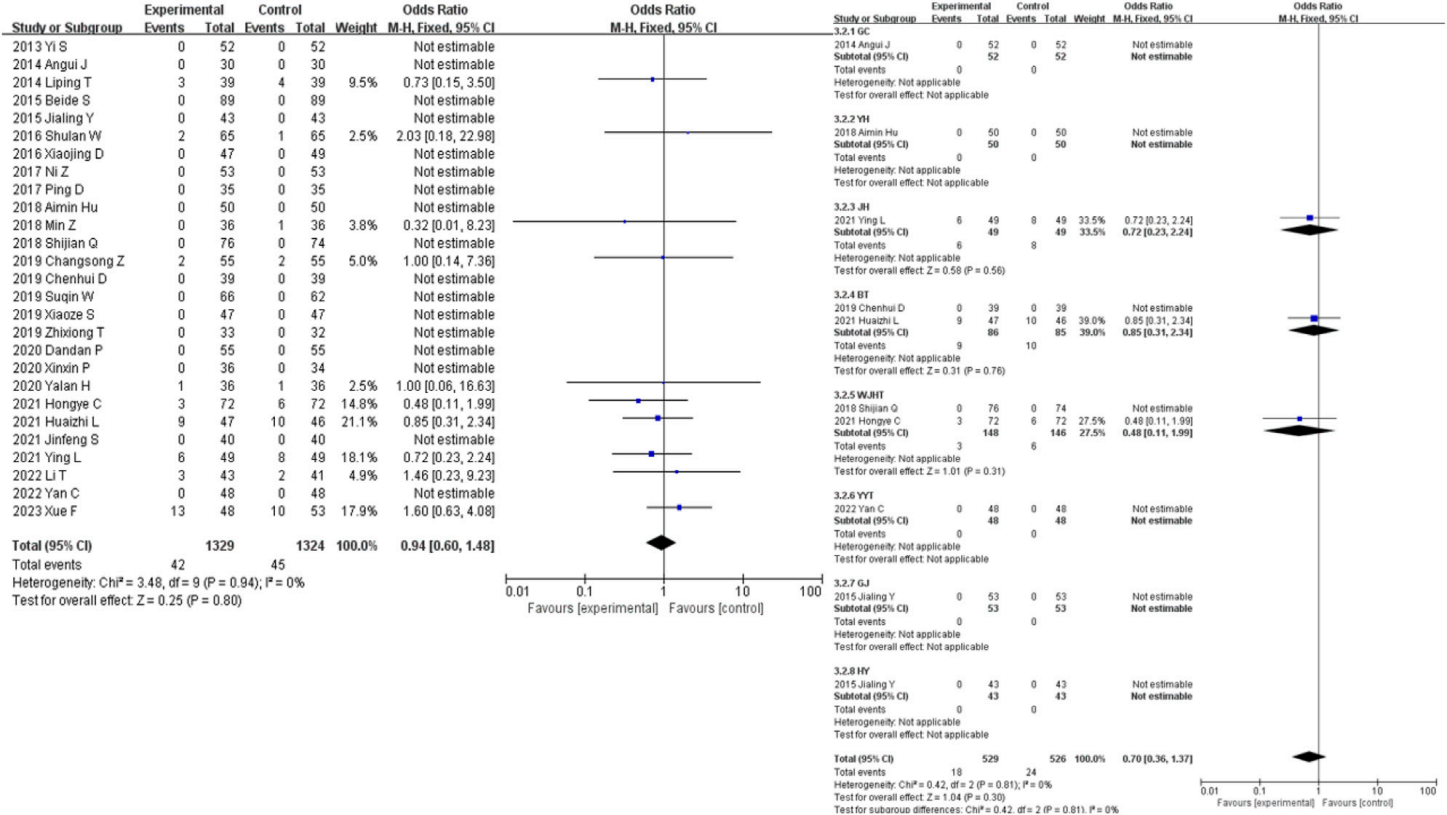
Adverse events forest plot & adverse events prescription forest plot.

A total of 28 studies were included in the analysis of the adverse event incidence outcome measure. For a further specific adverse reaction reports please check the Supplementary Table S9. The results showed that there was no statistically significant difference in the occurrence of adverse events between the intervention group and the control group for the treatment of diabetic nephropathy (OR = 0.94, 95% CI [0.60, 1.48], *I* ^*2*^ = 0%, *p* = 0.94). Sensitivity analysis did not reveal any significant sources of sensitivity. Subgroup analysis based on the composition of the traditional Chinese medicine decoctions showed that the WJHT group had the most significant reduction in the occurrence of adverse events compared to the control group (OR = 0.48, 95% CI [0.11, 1.99], *I* ^*2*^ = 0%, *p* = 0.76), while the BT group had the least significant reduction (OR = 0.85, 95% CI [0.31, 2.34], *I* ^*2*^ = 0%, *p* = 0.005) (Figure 4).

### 3.5 Secondary outcomes

**FIGURE 5:**
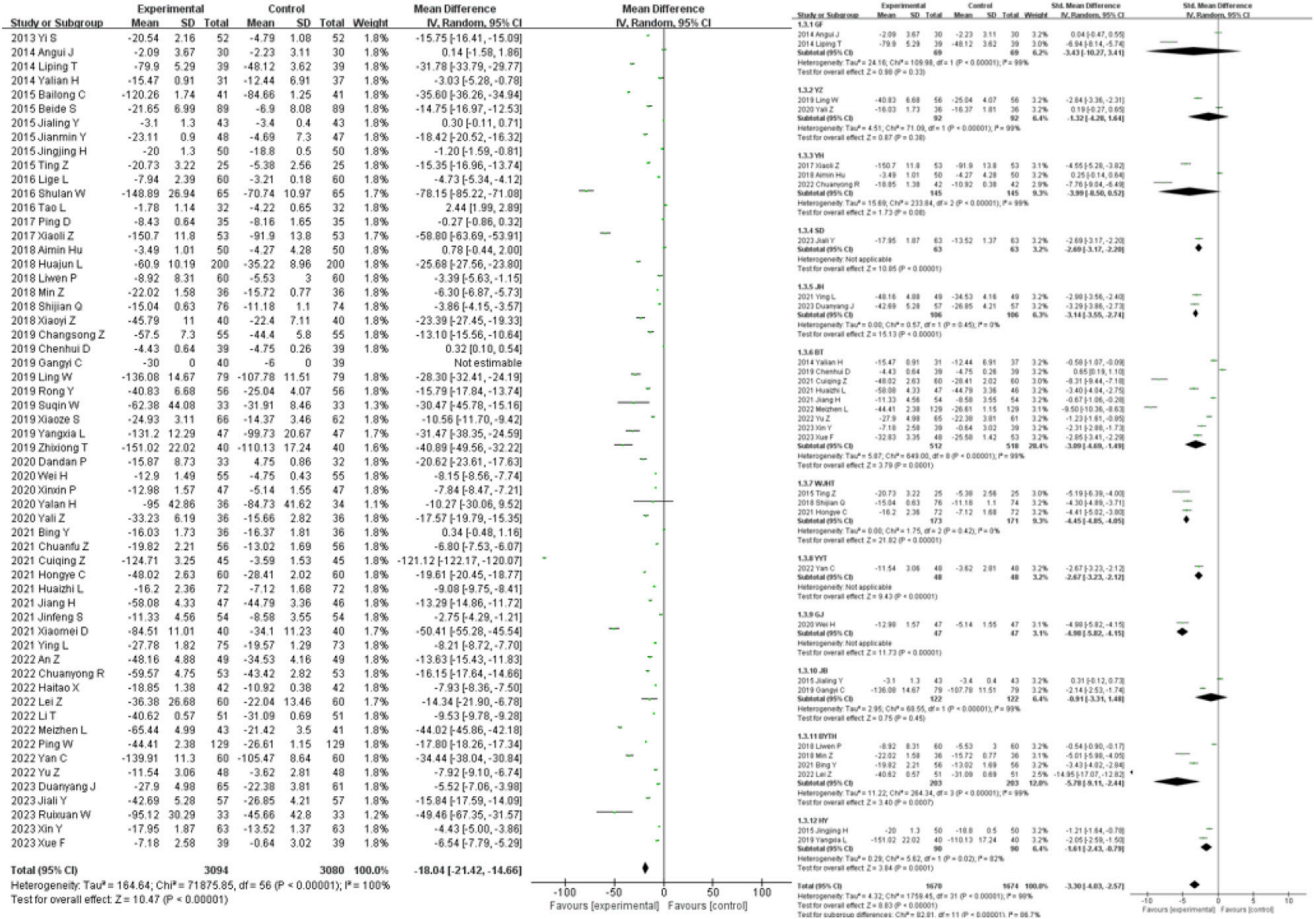
SCR forest plot & SCR prescription forest plot.

58 studies were included in the analysis of the SCR outcome measure. The results showed that traditional Chinese medicine decoctions as an adjunctive treatment were superior to the control group in reducing SCR in patients with diabetic nephropathy (MD = −18.04, 95% CI [-21.42, −14.66], *I* ^*2*^ = 100%, *p* < 0.00001). Subgroup analysis based on the composition of the traditional Chinese medicine decoctions showed that there was no statistically significant difference in the reduction of SCR between the intervention and control groups in the GF group, YZ group, YH group, and JB group (*p* = 0.33, *p* = 0.38, *p* = 0.08, *p* = 0.45, respectively). The most significant reduction in SCR compared to the control group was observed in the BYTH group (SMD = −5.78, 95% CI [-9.11, −2.44], *I* ^*2*^ = 99%, *p* = 0.0007), while the least significant reduction was observed in the JB group (SMD = −0.91, 95% CI [-3.31, 1.48], *I* ^*2*^ = 99%, *p* = 0.45) (Figure 5).

**FIGURE 6:**
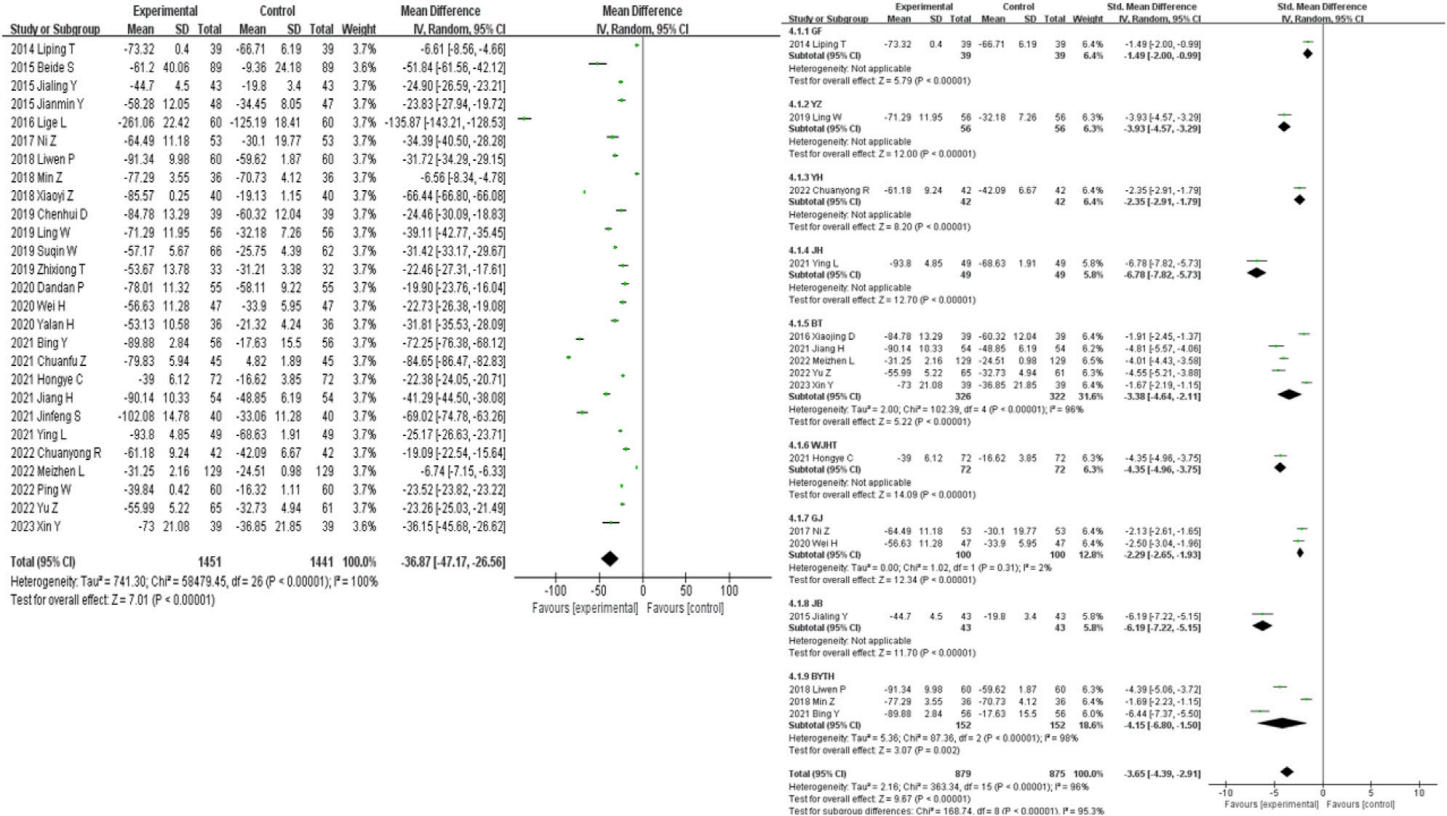
UAER forest plot & UAER prescription forest plot.

27 studies were included in the analysis of the UAER outcome measure. The results showed that traditional Chinese medicine decoctions as an adjunctive treatment were superior to the control group in reducing UAER in patients with diabetic nephropathy (MD = −36.87, 95% CI [-47.17, −26.56], *I* ^*2*^ = 100%, *p* < 0.00001). Subgroup analysis based on the composition of the traditional Chinese medicine decoctions showed that all subgroups of traditional Chinese medicine were able to significantly reduce UAER compared to the control group (*p* < 0.001). The most significant reduction in UAER compared to the control group was observed in the JH group (SMD = −6.78, 95% CI [-7.82, −5.73], *p* < 0.00001), while the least significant reduction was observed in the GF group (SMD = −1.49, 95% CI [-2.00, −0.99], *p* < 0.00001) (Figure 6).

**FIGURE 7:**
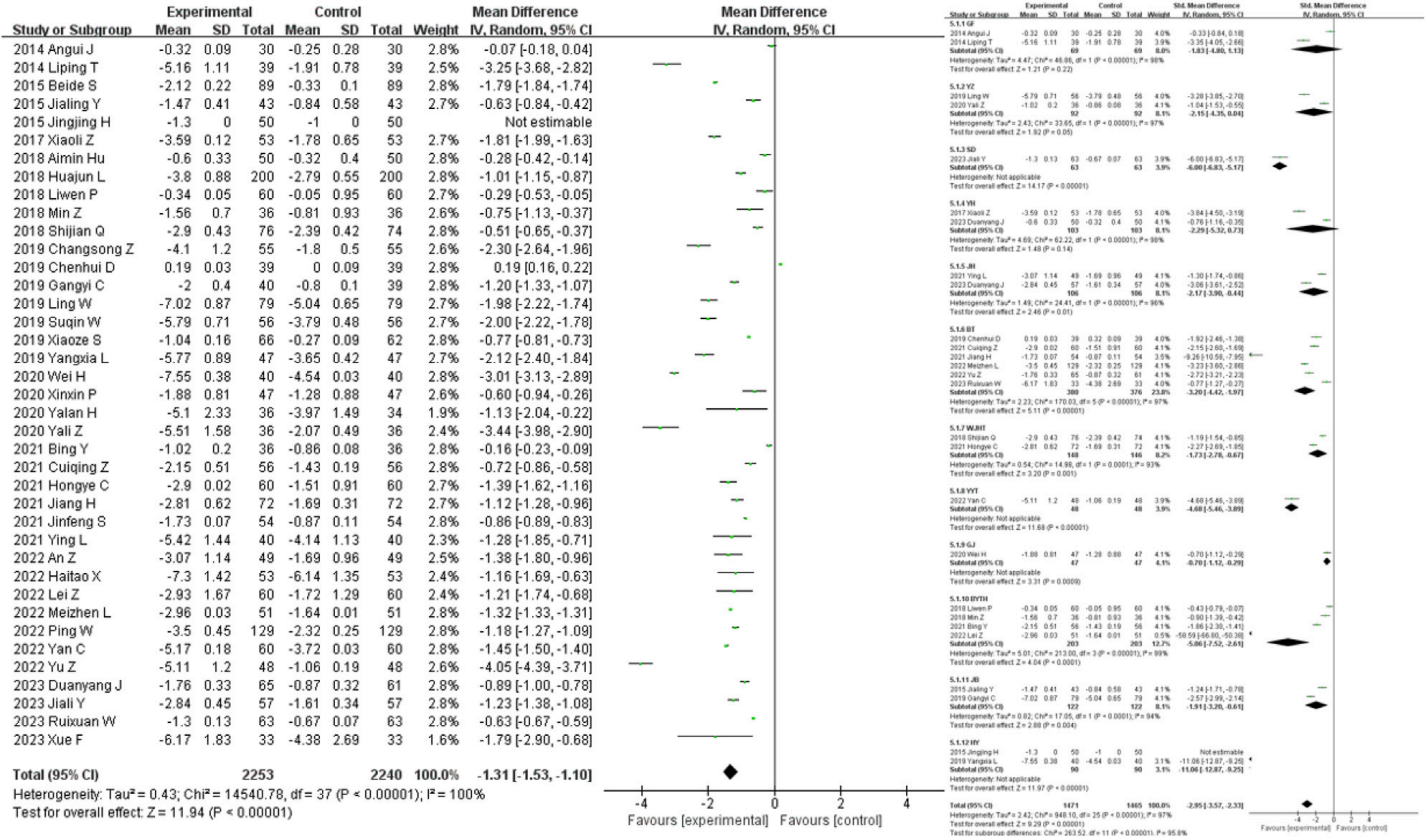
BUN forest plot & BUN prescription forest plot.

40 studies were included in the analysis of the BUN outcome measure. The results showed that traditional Chinese medicine decoctions as an adjunctive treatment were superior to the control group in reducing BUN levels in patients with diabetic nephropathy (MD = −1.31, 95% CI [-1.53, −1.10], *I* ^*2*^ = 100%, *p* < 0.00001). Subgroup analysis based on the composition of the traditional Chinese medicine decoctions showed that there was no statistically significant difference in the reduction of BUN levels between the GF group, YZ group, YH group, and the control group (*p* = 0.22, *p* = 0.05, *p* = 0.14). The most significant reduction in BUN levels compared to the control group was observed in the HY group (SMD = −11.06, 95% CI [-12.87, −9.25], *p* < 0.00001), while the least significant reduction was observed in the GJ group (SMD = −0.70, 95% CI [-1.12, −0.29], *p* = 0.0009) (Figure 7).

**FIGURE 8:**
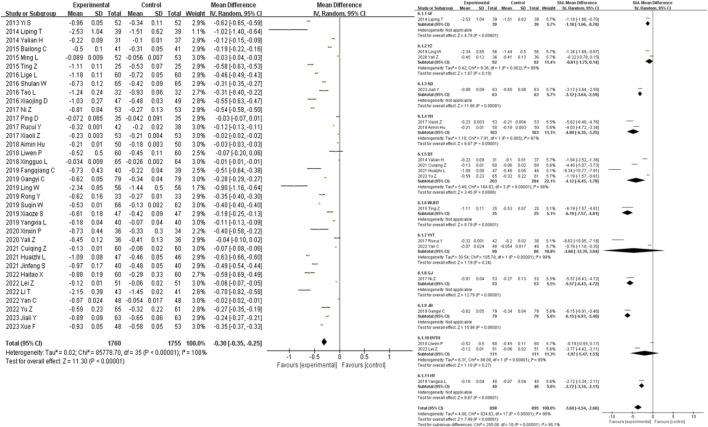
24h-utp forest plot & 24h-utp prescription forest plot.

36 studies were included in the analysis of the 24 h-utp outcome measure. The results showed that traditional Chinese medicine decoctions as an adjunctive treatment were superior to the control group in reducing 24 h-utp levels in patients with diabetic nephropathy (MD = −0.30, 95% CI [-0.35, −0.25], *I* ^*2*^ = 100%, *p* < 0.00001). Subgroup analysis based on the composition of the traditional Chinese medicine decoctions showed that there was no statistically significant difference in the reduction of 24 h-utp levels between the YZ group, YYT group, BYTH group, and the control group (*p* = 0.10, *p* = 0.24, *p* = 0.27). The most significant reduction in 24 h-utp levels compared to the control group was observed in the WJHT group (SMD = −6.19, 95% CI [-7.57, −4.81], *p* < 0.00001), while the least significant reduction was observed in the YZ group (SMD = −0.81, 95% CI [-1.75, 0.14], *I* ^*2*^ = 89%, *p* = 0.10) (Figure 8).

**FIGURE 9:**
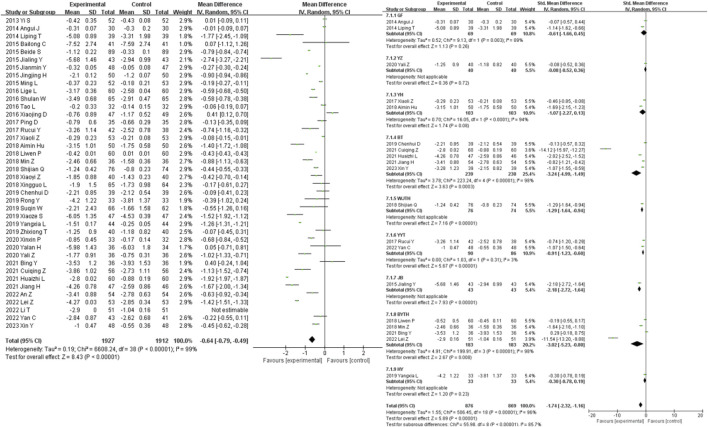
FPG forest plot & FPG prescription forest plot.

41 studies were included in the analysis of the FPG outcome measure. The results showed that traditional Chinese medicine decoctions as an adjunctive treatment were superior to the control group in reducing FPG levels in patients with diabetic nephropathy (MD = −0.64, 95% CI [-0.79, −0.49], *I* ^*2*^ = 100%, *p* < 0.00001). Subgroup analysis based on the composition of the traditional Chinese medicine decoctions showed that there was no statistically significant difference in the reduction of FPG levels between the GF group, YZ group, YH group, and HY group compared to the control group (*p* = 0.26, *p* = 0.72, *p* = 0.08, *p* = 0.23). The most significant reduction in FPG levels compared to the control group was observed in the BT group (SMD = −3.24, 95% CI [-4.99, −1.49], *I* ^*2*^ = 98%, *p* < 0.00001), while the least significant reduction was observed in the YZ group (SMD = −0.08, 95% CI [-0.52, 0.36], *p* = 0.72) (Figure 9).

**FIGURE 10:**
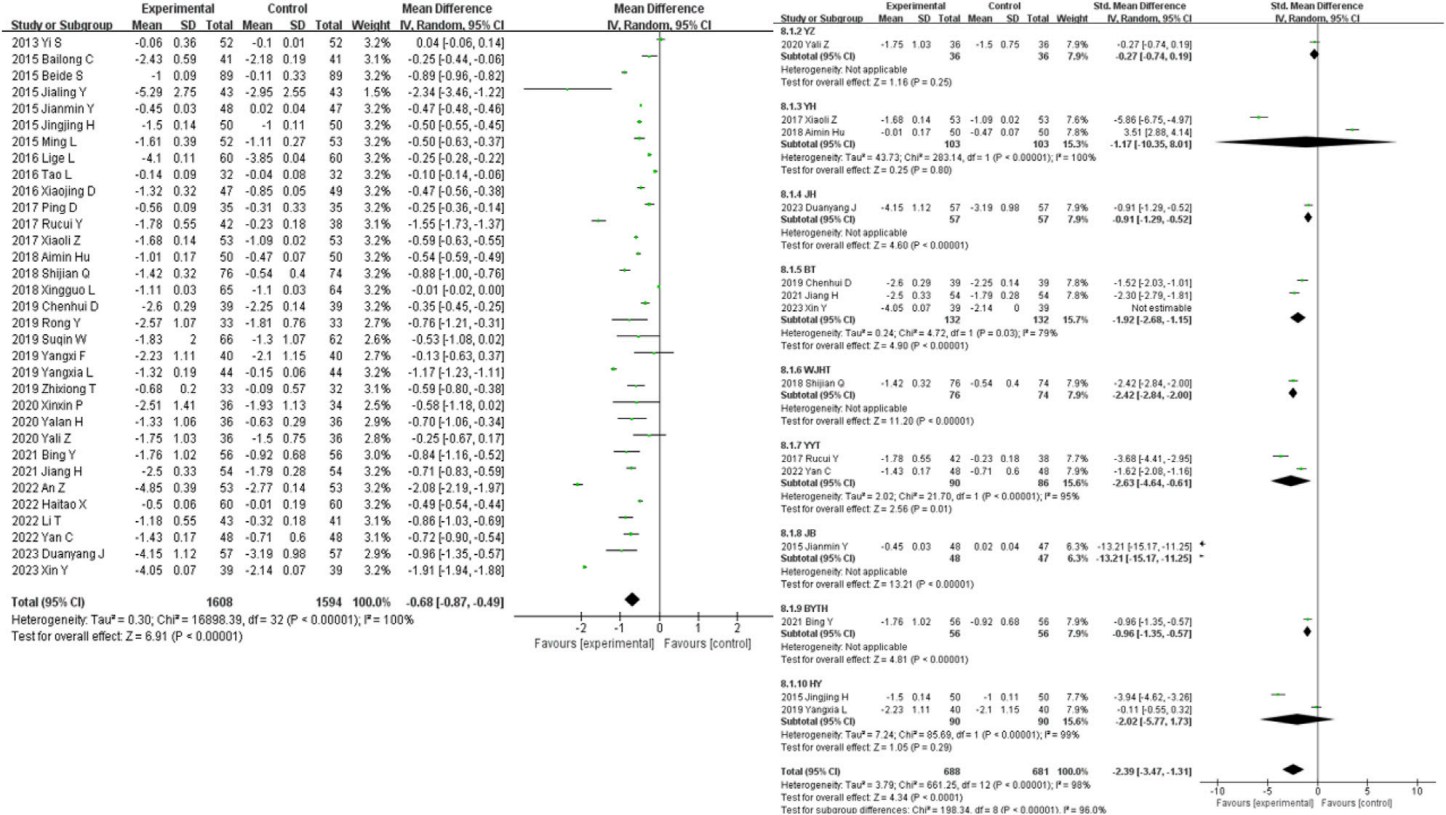
HbA1c forest plot & HbA1c prescription forest plot.

33 studies were included in the analysis of the HbA1c outcome measure. The results showed that traditional Chinese medicine decoctions as an adjunctive treatment were superior to the control group in reducing HbA1c levels in patients with diabetic nephropathy (MD = −0.68, 95% CI [-0.87, −0.49], *I* ^*2*^ = 100%, *p* < 0.00001). Subgroup analysis based on the composition of the traditional Chinese medicine decoctions showed that there was no statistically significant difference in the reduction of HbA1c levels between the GF group, YZ group, and HY group compared to the control group (*p* = 0.25, *p* = 0.80, *p* = 0.29). The most significant reduction in HbA1c levels compared to the control group was observed in the JB group (SMD = −13.21, 95% CI [-15.17, −11.25], *p* < 0.00001), while the least significant reduction was observed in the YZ group (SMD = −0.27, 95% CI [-0.74, 0.19], *p* = 0.80) (Figure 10).

### 3.6 Publication bias and sensitivity analysis

**FIGURE 11:**
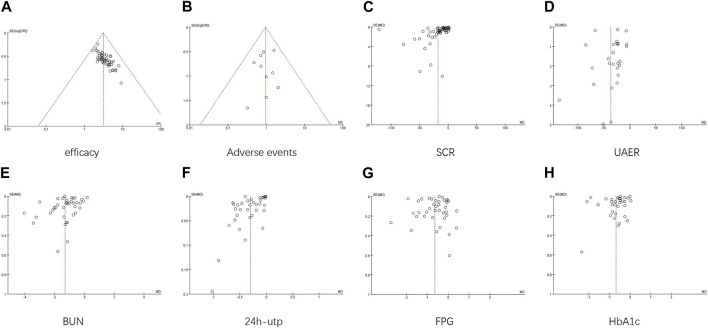
Funnel plot.

**FIGURE 12:**
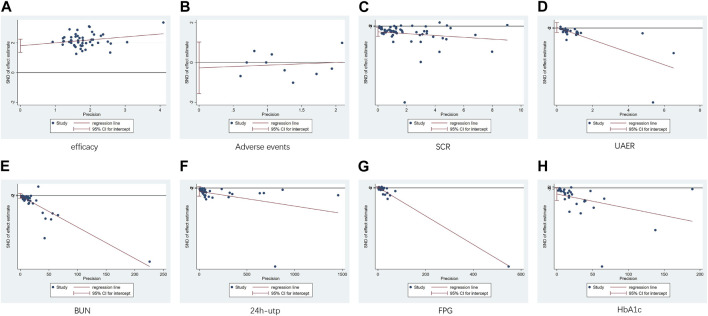
Egger plot.

The funnel plots showed asymmetry for SCR and UAER (Figure 11C; Figure 11D), indicating potential publication bias. The symmetry of the other plots was acceptable. Egger’s test was conducted to assess the publication bias. The results revealed significant publication bias for clinical efficacy and SCR (*p* < 0.00001, *p* = 0.012) (Figure 12A; Figure 12C), while no publication bias was observed for the remaining results (*p* = 0.642, *p* = 0.831, *p* = 0.742, *p* = 0.165, *p* = 0.092, *p* = 0.061) (Figures 12B, D, E, F, G, H). Sensitivity analysis using the one-by-one exclusion method did not reveal any significant sources of sensitivity.

## 4 Discussion

China has become the country with the highest number of diabetes patients in the world, and it is estimated that by 2045, there will be 170 million diabetes patients in China (International Diabetes Federation, 2023). Diabetic nephropathy, as a common complication of diabetes, is also one of the leading causes of death among diabetes patients, imposing a heavy social and economic burden on China and the world. Given this burden, timely diagnosis, treatment, and management of diabetic nephropathy in its early stages are of significant importance to improve symptoms, enhance quality of life, and improve prognosis for diabetic nephropathy patients. Traditional Chinese medicine has been widely used in the treatment of diabetic nephropathy in China for a long time. Diabetic nephropathy falls within the TCM categories of “xiao ke” (wasting and thirst disorder), “shui zhong” (odema), “xu lao” (asthenia), “guan ge” (urinary block and vomiting). Zhang Jingyue mentioned in the *Classified Canon* that if the wasting and thirst disorder is severe and uncontrolled, it will affect the liver and kidneys (Jiangyi et al., 2022).

Review by Wang B (Wang et al., 2011) reported that Chinese medicines could relieve several symptoms of diabetic nephropathy, improve the quality of life, reduce proteinuria levels and kidney damage, and further improve renal function via multiple pathways. There are many meta-analysis studies of other TCM dosage forms for treating diabetic nephropathy. For example, Long C’s network meta-analysis (Long et al., 2023) reported that the total effective rate of combined Salvia miltiorrhiza injection, Danshen-Chuanxiongqin injection, Danhong injection, Huangqi injection, and Shenkang injection combined with alprostadil injection (PGE1) was better than PGE1 alone. Within all subgroups, they found that PGE1+Shenkang injection was most effective for glomerular filtration function, while PGE1+Danhong injection was most effective for urinary protein-related indices. Shi RY’s study (Shi et al., 2023) showed that combining western medicine and Chinese patent medicine was superior to western medicine alone in reducing serum creatinine (Scr), blood urea nitrogen (BUN), and urinary albumin excretion rate (UAER), and improving the total effective rate of treatment. The focus of our study on TCM decoctions is due to the fact that TCM decoctions are a widely used clinical dosage form in China, and it has been found that the supramolecular structure in a TCM decoction can not only serve as a drug carrier to promote the absorption and distribution of medicinal components but may also exhibit biological activities superior to those of single active ingredients or their physical mixtures (Gao et al., 2022).

This study found that the adjunctive use of traditional Chinese medicine decoctions significantly improved clinical efficacy and reduced levels of SCR, BUN, UAER, 24 h-utp, FPG, and HbA1c in the treatment of diabetic nephropathy. The safety profile of traditional Chinese medicine decoctions was not statistically different from the control group. Sensitivity analysis did not reveal any significant sources of sensitivity. However, there was serious publication bias in terms of clinical efficacy and SCR results, while no significant publication bias was observed in the remaining results.

In this study, it was found that the Chinese medicines used as adjunctive treatment for diabetic nephropathy were mostly tonic, blood-activating, astringent, dampness-resolving, interior heat-clearing, and purgative medicines.

The top five Chinese medicine in terms of frequency were *Astragalus mongholicus Bunge* [Fabaceae; Astragali mongholici radix](58 times), *Salvia miltiorrhiza Bunge* [Lamiaceae; Radix et rhizoma salviae miltiorrhizae] (42 times), *Dioscorea oppositifolia L.* [Dioscoreaceae; Dioscoreae rhizoma] (38 times), *Poria cocos (Schw.) Wolf* [Polyporaceae; Poria] (38 times), and *Cornus officinalis Siebold & Zucc.* [Cornaceae; Corni fructus] (35 times).

*Astragalus mongholicus Bunge* [Fabaceae; Astragali mongholici radix] functions as a tonic for the spleen qi and as diuretic for resolving edema. Its main active metabolites include Astragalus polysaccharides, astragaloside IV and Astragalus flavone. Studies have shown that Astragalus polysaccharides can reduce the expression of inflammatory cytokines and inhibit the TLR4/NF-κB pathway, thereby alleviating renal inflammation and reducing kidney damage (Guo M. et al., 2023; Guo C. et al., 2023). Astragaloside IV is selected as a chemical marker in the Chinese Pharmacopoeia for quality control purposes. It has been proven to improve diabetic kidney disease by counteracting oxidative stress, alleviating endoplasmic reticulum stress, regulating calcium homeostasis, reducing inflammation, improving vascular function, and improving the transition from epithelial to mesenchymal cells (Gao et al., 2023). Astragalus flavone reduce oxidative damage induced by high glucose levels by protecting intracellular antioxidant enzyme activity and enhancing endogenous antioxidant function (Tang et al., 2018).

According to *Ben Cao Zheng Yi*, *Salvia miltiorrhiza Bunge* [Lamiaceae; Salviae Miltiorrhizae Radix et Rhizoma] can internally reach the organs, transform stasis, and relieve obstructions, while externally, it benefits the joints and promotes circulation in the meridians. It possesses the abilities to transform stasis, clear heat, relieve abscesses, and calm restlessness. The active metabolites in *Salvia miltiorrhiza Bunge* [Lamiaceae; Radix et rhizoma salviae miltiorrhizae] mainly consist of tanshinones and phenolic acids. Tanshinones can delay the progression of diabetic kidney disease by inhibiting Txnip/NLRP3 inflammasomes (Wu et al., 2023), while phenolic acids can inhibit AGE-RAGE and restore glomerular endothelial function, alleviating renal structural deterioration and effectively improving early-stage diabetic nephropathy (Hou et al., 2017).

*Dioscorea oppositifolia L.* [Dioscoreaceae; Dioscoreae rhizoma] also known as Chinese yam, serves both as a food and a medicinal botanical drug. It can tonify qi, nourish yin, and strengthen the lungs, spleen and kidneys. It is rich in various bioactive metabolites and has effects such as antioxidant, anti-aging, and immune regulation. The Diosgenin from Dioscorea oppositifolia L improves diabetic nephropathy by enhancing autophagy and mitochondrial autophagy, as well as improving mitochondrial dynamics in a CaMKK2-dependent manner (Zhong et al., 2023).

*Poria cocos (Schw.) Wolf* [Polyporaceae; Poria] possesses diuretic and dampness-draining properties, calming the heart and relieving restlessness. It is mainly used to treat odema and phlegm-fluid disorders. The main metabolites of *Poria cocos (Schw.) Wolf* [Polyporaceae; Poria] are Wolfiporia cocos polysaccharide can inhibit the over-expression of the Bax gene in the renal tissues of diabetic mice, thus inhibiting the trend of cellular apoptosis in diabetic kidney disease and demonstrating a certain preventive effect (Huang et al., 2016).

*Cornus officinalis Siebold & Zucc.* [Cornaceae; Corni fructus] has a sour and sweet taste, a slightly warm property, and belongs to the liver and kidney meridians. It functions as a tonic for kidney qi, nurishing kidney yin, semen-secure and urine-astringing. Extracts of *Cornus officinalis Siebold & Zucc.* [Cornaceae; Corni fructus] can inhibit the expression of FN and IL-50 in mesangial cells stimulated by high glucose, thereby improving diabetic nephropathy (Ma et al., 2014).

This study included 66 clinical randomized controlled trials of Chinese medicine decoctions as adjunctive treatment for diabetic nephropathy and conducted a comprehensive analysis. It is currently the largest meta-analysis in this field, confirming the effectiveness and safety of Chinese medicine decoctions as adjunctive treatment for diabetic nephropathy. The study explored the sources of heterogeneity through multidimensional analysis, providing evidence-based medicine evidence for further clinical research. However, this study also has some limitations. Due to the significant differences in the composition and dose of the Chinese medicine decoctions included in the studies, there was high heterogeneity in the results. Although most studies lacked clarity regarding the blinding and allocation scheme, as presented in the quality assessment graph, there was no significant impact on our outcome analysis. Most studies sources were searched from Chinese databases consisted of small-sample, single-center studies, leading to publication bias and regional selection bias. Further high-quality experiments are needed to increase the level of evidence.

## 5 Conclusion

In conclusion, this study demonstrates that the combined use of Western medicine and Chinese medicine decoctions can increase clinical efficacy and reduce SCR, UAER, BUN, 24 h-utp, FPG, and HbA1c levels, surpassing the efficacy of using Western medicine alone, without posing significant risks. These results indicate that Chinese medicine decoctions are a safe and effective adjunctive therapy for treating diabetic nephropathy.

## Data Availability

The original contributions presented in the study are included in the article/Supplementary Material, further inquiries can be directed to the corresponding authors.
