# Supplementary material for: Efficacy and safety of traditional Chinese medicine decoction as an adjuvant treatment for diabetic nephropathy: a systematic review and meta-analysis of randomized controlled trials
Source: Front Pharmacol. 2024 May 9;15:1327030. doi: 10.3389/fphar.2024.1327030 (PMC11111926; doi:10.3389/fphar.2024.1327030)
Supplement: Supplementary file 1 [file DataSheet1.pdf]

Supplementary Material

1 Supplementary Data

Supplementary Material should be uploaded separately on submission. Please include any supplementary data, figures and/or tables.

Supplementary material is not typeset so please ensure that all information is clearly presented, the appropriate caption is included in the file and not in the manuscript, and that the style conforms to the rest of the article.

2 Supplementary Figures and Tables

Table 1 search strategies of database

	Search strategy of PubMed
#1	"Diabetic Nephropathy" [MeSH Terms]
#2	(Diabetic Nephropathy [Title/Abstract]) OR (Nephropathy, Diabetic[Title/Abstract]) OR (Nephropathies, Diabetic[Title/Abstract]) OR (Diabetic Nephropathies[Title/Abstract]) OR (Diabetic Kidney Disease[Title/Abstract]) OR (Diabetic Kidney Diseases[Title/Abstract]) OR (Kidney Disease, Diabetic[Title/Abstract]) OR (Kidney Diseases, Diabetic[Title/Abstract]) OR (Diabetic Glomerulosclerosis[Title/Abstract]) OR (Glomerulosclerosis, Diabetic[Title/Abstract]) OR (Intracapillary Glomerulosclerosis[Title/Abstract]) OR (Nodular Glomerulosclerosis[Title/Abstract]) OR (Glomerulosclerosis, Nodular[Title/Abstract]) OR (Kimmelstiel-Wilson Syndrome[Title/Abstract]) OR (Kimmelstiel Wilson Syndrome[Title/Abstract]) OR (Syndrome, Kimmelstiel -Wilson[Title/Abstract]) OR (Kimmelstiel-Wilson Disease[Title/Abstract]) OR (Kimmelstiel Wilson Disease[Title/Abstract])
#3	#1 OR #2
#4	"Traditional Chinese Medicine" [MeSH Terms]
#5	(Traditional Chinese Medicine[Title/Abstract]) OR (Chung I Hsueh[Title/Abstract]) OR (Hsueh, Chung I[Title/Abstract]) OR (Traditional Medicine, Chinese[Title/Abstract]) OR (Zhong Yi Xue[Title/Abstract]) OR (Chinese Traditional Medicine[Title/Abstract]) OR (Chinese Medicine, Traditional[Title/Abstract]) OR (Chinese Medicine Decoction [Title/Abstract]) OR (Decoction, Chinese Medicine[Title/Abstract]) OR (Chinese Botanical Medicine[Title/Abstract]) OR (Botanical, Chinese Medicine[Title/Abstract])
#6	#4 OR #5
#7	(randomized controlled trial[pt] OR controlled clinical trial[pt] OR randomized[tiab] OR placebo[tiab] OR drug therapy[sh] OR randomly[tiab] OR trial[tiab] OR groups[tiab]) OR Intervention Study[pt] OR Clinical Study[pt] NOT (animals[mh] NOT humans[mh])
#8	#3 AND #6 AND #7
	Search strategy of Web of Science
#1	TS= (Diabetic Nephropathy OR Diabetic Nephropathies OR Diabetic Kidney Disease OR Diabetic Kidney Diseases OR Diabetic Glomerulosclerosis OR Intracapillary Glomerulosclerosis OR Nodular Glomerulosclerosis OR Kimmelstiel-Wilson Syndrome OR Kimmelstiel Wilson Syndrome OR Syndrome, Kimmelstiel-Wilson OR Kimmelstiel-Wilson Disease OR Kimmelstiel Wilson Disease)

#2	TS= (Traditional Chinese Medicine OR Chung I Hsueh OR Zhong Yi Xue OR Chinese Traditional Medicine OR Chinese Medicine Decoction OR Chinese Botanical Medicine)
#3	TS= (randomized controlled trial OR controlled clinical trial OR randomized OR placebo OR drug therapy OR randomly OR trial OR groups OR Intervention Study OR Clinical Study OR Intervention Study OR Clinical Study NOT animals NOT humans)
#4	#1 AND #2 AND #3
	Search strategy of Cochrane Library
#1	MeSH descriptor: [Diabetic Nephropathy] explode all trees
#2	(Nephropathies, Diabetic):ti,ab,kw OR (Nephropathy, Diabetic):ti,ab,kw OR (Diabetic Nephropathies):ti,ab,kw OR (Diabetic Kidney Disease):ti,ab,kw OR Diabetic Kidney Diseases):ti,ab,kw OR (Kidney Disease, Diabetic):ti,ab,kw OR (Kidney Diseases, Diabetic):ti,ab,kw OR (Diabetic Glomerulosclerosis):ti,ab,kw OR (Glomerulosclerosis, Diabetic):ti,ab,kw OR (Intracapillary Glomerulosclerosis):ti,ab,kw OR (Nodular Glomerulosclerosis):ti,ab,kw OR (Glomerulosclerosis, Nodular):ti,ab,kw OR (Kimmelstiel-Wilson Syndrome):ti,ab,kw OR (Kimmelstiel Wilson Syndrome):ti,ab,kw OR (Syndrome, Kimmelstiel-Wilson):ti,ab,kw OR (Kimmelstiel-Wilson Disease):ti,ab,kw OR (Kimmelstiel Wilson Disease):ti,ab,kw
#3	#1 OR #2
#4	MeSH descriptor: [Traditional Chinese Medicine] explode all trees
#5	(Chung I Hsueh):ti,ab,kw OR (Hsueh, Chung I):ti,ab,kw OR (Traditional Medicine, Chinese):ti,ab,kw OR (Zhong Yi Xue):ti,ab,kw OR (Chinese Traditional Medicine):ti,ab,kw OR (Chinese Medicine, Traditional):ti,ab,kw OR (Chinese Medicine Decoction):ti,ab,kw OR (Decoction, Chinese Medicine):ti,ab,kw OR (Chinese Botanical Medicine):ti,ab,kw OR (Botanical, Chinese Medicine)
#6	#4 OR #5
#7	MeSH descriptor: [randomized controlled trial] explode all trees
#8	(randomized controlled trial):ti,ab,kw OR (controlled clinical trial):ti,ab,kw):ti,ab,kw OR (randomized):ti,ab,kw OR (placebo):ti,ab,kw OR (drug therapy):ti,ab,kw OR (randomly):ti,ab,kw OR (trial):ti,ab,kw OR (groups):ti,ab,kw OR (Intervention Study):ti,ab,kw OR (Clinical Study):ti,ab,kw OR (Intervention Study):ti,ab,kw OR (Clinical Study):ti,ab,kw NOT (animals)
#9	#7 OR #8
#10	#3 AND #6 AND #9
	Search strategy of Embase
#1	‘Diabetic Nephropathy ':ab,kw,ti OR 'Nephropathy, Diabetic':ab,kw,ti OR 'Nephropathies, Diabetic':ab,kw,ti OR 'Diabetic Nephropathies':ab,kw,ti OR 'Diabetic Kidney Disease':ab,kw,ti OR '(Diabetic Kidney Diseases':ab,kw,ti OR 'Kidney Disease, Diabetic':ab,kw,ti OR 'Kidney Diseases, Diabetic':ab,kw,ti OR 'Diabetic Glomerulosclerosis':ab,kw,ti OR 'Glomerulosclerosis, Diabetic':ab,kw,ti OR 'Intracapillary Glomerulosclerosis':ab,kw,ti OR 'Nodular Glomerulosclerosis':ab,kw,ti OR 'Glomerulosclerosis, Nodular':ab,kw,ti OR 'Kimmelstiel-Wilson Syndrome':ab,kw,ti OR 'Kimmelstiel Wilson Syndrome':ab,kw,ti OR 'Syndrome, Kimmelstiel-Wilson':ab,kw,ti OR 'Kimmelstiel-Wilson Disease':ab,kw,ti OR 'Kimmelstiel Wilson Disease’
#2	‘Traditional Chinese Medicine':ab,kw,ti OR 'Chung I Hsueh':ab,kw,ti OR 'Hsueh, Chung I':ab,kw,ti OR '(Traditional Medicine, Chinese':ab,kw,ti OR 'Zhong Yi Xue':ab,kw,ti OR 'Chinese Traditional Medicine':ab,kw,ti OR 'Chinese Medicine, Traditional':ab,kw,ti OR 'Chinese Medicine Decoction':ab,kw,ti OR 'Decoction, Chinese Medicine':ab,kw,ti OR 'Chinese Botanical Medicine':ab,kw,ti OR 'Botanical, Chinese Medicine’
#3	'crossover procedure':de OR 'double-blind procedure':de OR 'randomized controlled trial':de OR 'single-blind procedure':de OR (random* OR factorial* OR crossover* OR cross NEXT/1 over* OR placebo* OR doubl* NEAR/1 blind* OR singl* NEAR/1 blind* OR assign* OR allocat* OR volunteer*):de,ab,ti
#4	#1 AND #2 AND #3
	Search Strategy of China National Knowledge Infrastructure (CNKI)
#1	(篇关摘=糖尿病肾病 + 消渴肾病) AND (篇关摘=中医 + 中药 + 中草药) AND (篇关摘=随机对照试验 + 临床试验)
	Search Strategy of Wanfang database
#1	检索表达式（中英文扩展&主题词扩展）：全部:(糖尿病肾病 + 消渴肾病) and 全部:(中医 + 中药 + 中草药) and 全部:(随机对照试验 + 临床试验)

Table 2 table of standard terminologies for Chinese Pinyin

pinyin	standard terminologies
xiao ke	wasting and thirst disorders
shui zhong	odema
xu lao	asthenia
guan ge	urinary block and vomiting
Guizhi Fuling formula	Cinnamon twig and Poria formula
Jiangtang Kangshen formula	hypoglycemic and benefit the kidney formua
Yiqi Zishen formula	benefit qi, nourish and supplement kidney yin formula
Qiju Dihuang formula	Fructus Lycii, Flos Chrysanthemi and Rehmannia formula
Yiqi Huayu formula	benefit qi and transform stasis formula
Tangshen formula	diabeteic nephropathy formula
Jianpi Yishen formula	strengthen the spleen and benefit kidney formula
Shenqi Dihuang formulate	Panax ginseng, Astragalus and Rehmannia formula
Danggui Dihuang formula	Chinese Angelica Six Yellow formula
Buyuan Tongluo formula	benefit Yuan-primordial qi and unblock collaterals formula
Yiqi Yangyin Tongluo formula	benefit qi, nourish yin and unblock collaterals formula
Shenqi Dihuang formula	Panax ginseng, Astragalus and Rehmannia formula
Shenmai Dihuang formula	Panax ginseng,Radix Ophiopogonis and Rehmannia formula
Shenqi Dihuang formula	Panax ginseng, Astragalus and Rehmannia formula
Qidi Shenkang formula	Astragalus and Rehmannia and strengthen the kidney formula
Zhenwu formula	true warrior formula
Shenqi Dihuang formula	Panax ginseng, Astragalus and Rehmannia formula
Buyang Huanwu formula	Yang-Supplementing and Five-Returning formula
Jianshen Huayu formula	strengthen the kidney and transform stasis formula

Shenqi Dihuang formulate	Panax ginseng, Astragalus and Rehmannia formula
Wenshen Jianpi Huayu Tongluo formula	warm the kidney, strengthen the spleen, transform stasis and unblock collaterals
Yiqi Huoxue Gushen formula	benefit qi,circulate blood and
Shenqi Dihuang formula	Panax ginseng, Astragalus and Rehmannia formula
Yiqi Yangyin Tongluo formula	benefit qi, nourish yin and unblock collaterals formula
Shenqi Dihuang formula	Panax ginseng, Astragalus and Rehmannia formula
Bushen Yiqi Tongluo Huazhuo formula	nourish the Kidney, benefit qi, unblock collaterals and transform turbidity formula
Shenqi Dihuang formula	Panax ginseng, Astragalus and Rehmannia formula
Jianpi Baoshen Tongluo formula	strengthen the spleen, preserve the kidney and unblock collaterals formula
Four Junzi formula plus Liuwei Dihuang formula	Four Gentleme formula combined with Six-Ingredient Rehmannia formula
Self-formulate formula	Self-formulated formula
Gushen Jianpi formula	secure the kidney, strengthen spleen and resolve dampness formula
Jiangtang Baoshen formula	hypoglycemic and preserve the kidney formua
Yiqi Yangyin Quyu formula	benefit qi, nurish yin and transform stasis formula
Shenqi Dihuang formula	Panax ginseng, Astragalus and Rehmannia formula
Huoxue Yishen formula	circulate blood and benetit the kidney formula

Table 3 characteristic table

study	Study type	treatment duration(w)	Case E/C	Age(y) E/C	Gender(M:F) E/C	DM duration(y) E/C	DN staging
2013 Yi S [7]	RCT	8	52/52	59.75±7.5/58.6±8.1	28:24/26:26	10. 1±5.3/9.8±6.1	IV
2014 Angui J [8]	RCT	4	30/30	58.8±4.5/57.3±4.4	13:17/14:16	7.55±3.21/7.61±3.32	/
2014 Liping T [9]	RCT	24	39/39	46. 16±11.92/47.76±10.97	28:11/24:15	9.87±3.47/10. 10±3.45	IV
2014 Yalian H [10]	RCT	24	31/37	54.23±6. 11/55.28±5.86	15:16/17:20	7.23±2.54/7.44±2.49	III
2015 Bailong C [11]	RCT	12	41/41	58.83±11.95/59.43±10.29	25:16/23:18	12.56±2.73/12.35±2.89	IV
2015 Beide S [12]	RCT	4	89/89	53.45±13.67/52.97±12.92	43:46/41:48	9.75±3.63/9.97±4.12	III~IV
2015 Jialing Y [13]	RCT	8	43/43	55.6±5.8/56. 1±5.6	24:19/22:21	/	III
2015 Jianmin Y [14]	RCT	4	48/47	58.5±11.4		/	III
2015 Jingjing H [15]	RCT	8	50/50	58. 1±1.0/58.8±1.3	26:24/25:25	7.2±0.6/7.4±0.9	III
2015 Ming L [16]	RCT	12	52/53	53. 1±11.5/52.5±10.9	30:23/29:32	10. 1±2.9/9.3±3.1	III
2015 Ting Z [17]	RCT	12	25/25	65.3±5.2/64.9±5.4	14:11/12:13	11.23±3. 14/12.02±3.35	IV

2016 Lige L [18]	RCT	8	60/60	56. 1±2.6/55.4±2.9	39:21/37:23	9.7±2.3/9.3±2.1	III
2016 Shulan W [19]	RCT	4	65/65	46.82±7.65/45.69±7.26	34:31/32:33	/	IV
2016 Tao L [20]	RCT	12	32/32	52. 1±16.2/51.9±17.3	18:14/17:15	/	IV
2016 Xiaojing D [21]	RCT	8	47/49	52.36±8. 17/53.96±9.04	21:26/23:26	6.24±2.31/6. 18±2.28	III
2017 Ni Z [22]	RCT	8	53/53	62.34±10.81/60. 13±9.15	30:23/32:21	6.4±2.3/6.6±1.1	/
2017 Ping D [23]	RCT	6	35/35	53. 10±13.20/51.40±12.50	19:16/18:17	9.8±2.2/8.8±2.0	III
2017 Rucui Y [24]	RCT	12	42/38	53.6±7.0/52.5±8.1	22:20/21:17	8.4±2.5/8. 1±2.2	III
2017 Xiaoli Z [25]	RCT	12	53/53	55.9±8.6/56.2±8.3	26:27/28:25	8.3±4.5/8. 1±4.3	III
2018 Aimin Hu [26]	RCT	24	50/50	55.98±9.60/57. 14±7.46	29:17/31:18	9.72±4.64/9.76±6.22	III~IV
2018 Huajun L [27]	RCT	8	200/200	69.32±3.79/69.27±3.75	129:71/133:67	5.59±1.23/5.57±1.22	/
2018 Liwen P [28]	RCT	52	60/60	55.8± 13.3/58.6±15.7	40:20/39:21	12.3±6.7/10. 1±8.9	III
2018 Min Z [29]	RCT	8	36/36	52.42±5.09/52.38±5.07	21:15/20:16	4.91±0.68/4.89±0.67	III
2018 Shijian Q [30]	RCT	12	76/74	48.62±5.93/48.43±5.88	37:39/36:38	7.56±1.86/7.47±1.83	III

2018 Xiaoyi Z [31]	RCT	4	40/40	53.05±6.87/55.23±7.50	22:18/19:21	7.83±2.64/6.86±3.08	III
2018 Xingguo L [32]	RCT	12	65/64	54~72	87:41	8~19	III~IV
2019 Changsong Z [33]	RCT	4	55/55	68.5±4.7/68.9±5.1	32:23/33:22	14.2±4.5/14.5±3.6	/
2019 Chenhui D [34]	RCT	12	39/39	58.67±6.28/59.07±5.79	22:17/23:16	7.47±2.66/7.68±2.41	III
2019 Fangqiang C [35]	RCT	12	39/40	63.35±8.02/62.95±7.24	22:18/19:20	/	/
2019 Gangyi C [36]	RCT	12	79/79	57.44±6.93/57.04±6.82	41:38/40:39	7.22±0.90/7.30±0.91	III~IV
2019 Jili S [37]	RCT	8	37/35	52. 14±3.73/53.28±3.46	17:20/17:18	6.42±2.08/6. 16±2.47	III
2019 Ling W [38]	RCT	12	56/56	52.8±12.9/54.3±14.1	31:25/33:23	8.4±6. 1/7.8±5.3	III~IV
2019 Rong Y [39]	RCT	8	33/33	50.36±8.54/52. 18±8.31	21:12/20:13	1.74±0.41/2.35±0.50	III
2019 Suqin W [40]	RCT	12	66/62	55.78±9.30/59.68±9.12	36:30/39:23	14. 12±7.25/12.81±8.14	III~IV
2019 Xiaoze S [41]	RCT	4	47/47	59.46±8. 14/60.75±7.93	30:17/27:20	7.65±1.71/7.37±1.42	III~IV
2019 Yangxi F [42]	RCT	4	40/40	59.56±4.90/59.87±4.77	19:21/18:22	11.61±6.01/11.45±6.12	/

2019 Yangxia L [43]	RCT	12	44/44	57. 13±8. 12/56.73±8.04	21:23/24:20	6.22±0.86/6. 12±0.83	IV
2019 Zhixiong T [44]	RCT	8	33/32	63. 1±7.6/62.4±7.7	20:13/32:18	7.3±2.2/6.9±2.1	III
2020 Dandan P [45]	RCT	24	55/55	56.55±8.48/56.24±8.67	28:27/30:25	8.86±3.41/8.72±3.58	III
2020 Wei H [46]	RCT	12	47/47	42.76±10.93/45.37±11.26	29:18/27:20	8.46±2.47/8.95±2.51	III
2020 Xinxin P [47]	RCT	8	36/34	57.7±9.7/60.0±7.4	18:18/16:18	8. 1±3.4/8.4±2.0	IV
2020 Yalan H [48]	RCT	12	36/36	50.84±6.92/52.45±8.46	22:14/20:16	8.26±3.51/7.48±2.74	III
2020 Yali Z [49]	RCT	12	36/36	57.03±7.27/56.41±7.09	25:11/21:15	8. 12±1.25/8.43±1.01	III
2021 Bing Y [50]	RCT	4	56/56	52.33±2. 11/52.54±2.08	27:29/31:25	3.02±0. 17/3. 11±0.17	IV
2021 Chuanfu Z [51]	RCT	12	45/45	49.63±8.29/50.58±7.41	25:20/21:24	8.74±4. 13/7.36±4.72	IV
2021 Cuiqing Z [52]	RCT	5	60/60	45.37±15.54/46. 12±16.42	31:29/27:33	9.54±3.27/9.32±3.43	III
2021 Hongye C [53]	RCT	12	72/72	62.03±5.01/61.52±4.88	41:31/43:29	8.25±1.49/8.33±1.54	/
2021 Huaizhi L [54]	RCT	12	47/46	50.04±3.92/49.35±3.61	27:20/25:21	11.43±1.81/11. 17±1.59	III

2021 Jiang H [55]	RCT	4	54/54	59.46±7.81/57.36±9.02	30:24/33:21	12.03±1.49/11.57±1.86	III
2021 Jinfeng S [56]	RCT	12	40/40	53.25±7.23/52.69±6.67	24:16/22:18	/	IV
2021 Xiaomei D [57]	RCT	12	75/73	58.62±5.91/57.93±5.72	47:33/44:36	7.91±0.86/7.86±0.83	III~IV
2021 Ying L [58]	RCT	12	49/49	54.29±4.09/53.37±4.18	20:29/17:32	2.51±1.00/2.38±0.93	III
2022 An Z [59]	RCT	12	53/53	48.00±9.82/50.25±8.83	29:24/26:27	11.64±2. 11/10.81±2.77	/
2022 Chuanyong R [60]	RCT	12	42/42	65. 1±5.2/64.7±4.7	25:17/23:19	14. 1±2.3/13.7±2.0	III
2022 Haitao X [61]	RCT	12	60/60	48.63±15.31/50.49±13.31	33:28/29:30	8.35±5.64/7.85±6.23	III~IV
2022 Lei Z [62]	RCT	8	51/51	55.82±10.32/56. 14±10.42	19:32/20:31	9.21±4.67/9.32±4.49	/
2022 Li T [63]	RCT	12	43/41	54.02±4.63/54. 16±4.76	26:17/23:18	7.93±0.86/8.21±0.93	III~IV
2022 Meizhen L [64]	RCT	12	129/129	/	/	/	III
2022 Ping W [65]	RCT	8	60/60	65.23±4.67/67.55±2.34	34:26/37:23	5.52 ±1.62/6. 13±1.38	/
2022 Yan C [66]	RCT	8	48/48	55.45±5.38/54.31±4.12	28:20/29:19	9.08±2.21/9.61±2.47	/
2022 Yu Z [67]	RCT	12	65/61	56.02±7.59/55.94±8.12	36:29/33:28	13.97±6.49/14.04±5.52	III~IV

2023 Duanyang J [68]	RCT	8	57/57	64. 12±5.32/64.24±5.41	32:25/31:26	3.37± 1.01/3.29±1.92	IV
2023 Jiali Y [69]	RCT	12	63/63	63.62±7.44/63.47±7.41	44:19/41:22	7.81±0.89/7.73±0.88	III
2023 Ruixuan W [70]	RCT	8	33/33	63.09±11.85/64.48±9.6	23:10:28:5	/	IV~V
2023 Xin Y [71]	RCT	12	39/39	62.20±8.36/61.46±7.74	23:16/25:14	8. 12±2. 16/8.29±2.27	III
2023 Xue F [72]	RCT	12	48/53	70.03±4.51/69.21±4.25	32:16/32:21	7. 16±2.35/7.35±2.14	III~IV

RCT:Randomized controlled trial; w:week; y:year; E:experimental group; C:control group; M:male; F:female; DM:diabetic Mellitus; DN:diabetic nephropathy.

Table 4 Citeria for assessing clinical effectiveness rates

Study	efficacy criteria
2013 Yi S	not mentioned
2014 Angui J	Guiding Principles for Clinical Research on New Chinese Medicines
2014 Liping T	not mentioned
2014 Yalian H	Criteria for the Diagnosis, Identification and Classification of Diabetic Nephropathy and Evaluation of Therapeutic Effect.
2015 Bailong C	not mentioned
2015 Beide S	not mentioned
2015 Jialing Y	Criteria for assessing the efficacy of treatment of xiao ke diseases.
2015 Jianmin Y	Criteria for the Diagnosis and Efficacy of Chinese Medicine Diseases
2015 Jingjing H	not mentioned
2015 Ming L	Criteria for the Diagnosis, Identification and Classification of Diabetic Nephropathy and Evaluation of Therapeutic Effect.
2015 Ting Z	Guidelines for Clinical Research on New Chinese Medicines for the Treatment of Diabetes Mellitus
2016 Lige L	Criteria for the Diagnosis, Identification and Classification of Diabetic Nephropathy and Evaluation of Therapeutic Effect.
2016 Shulan W	Draft Chinese and Western Medicine Diabetes Diagnostic and Treatment Standards
2016 Tao L	Criteria for the Diagnosis, Identification and Classification of Diabetic Nephropathy and Evaluation of Therapeutic Effect.
2016 Xiaojing D	Guiding Principles for Clinical Research on New Chinese Medicines
2017 Ni Z	Diabetic Nephropathy Diagnosis, Diagnostic Typing and Efficacy Assessment Criteria
2017 Ping D	Guiding Principles for Clinical Research on New Chinese Medicines
2017 Rucui Y	Guiding Principles for Clinical Research on New Chinese Medicines
2017 Xiaoli Z	Guiding Principles for Clinical Research on New Chinese Medicines
2018 Aimin Hu	Guiding Principles for Clinical Research of New Chinese Medicines (for Trial Implementation)
2018 Huajun L	Guiding Principles for Clinical Research of New Chinese Medicines (for Trial Implementation)

2018 Liwen P	Guiding Principles for Clinical Research of New Chinese Medicines (for Trial Implementation)
2018 Min Z	not mentioned
2018 Shijian Q	Guiding Principles for Clinical Research of New Chinese Medicines (for Trial Implementation)
2018 Xiaoyi Z	Guiding Principles for Clinical Research of New Chinese Medicines (for Trial Implementation)
2018 Xingguo L	not mentioned
2019 Changsong Z	Guiding Principles for Clinical Research of New Chinese Medicines (for Trial Implementation)
2019 Chenhui D	Guiding Principles for Clinical Research on New Chinese Medicines
2019 Fangqiang C	not mentioned
2019 Jili S	Guiding Principles for Clinical Research on New Chinese Medicines
2019 Ling W	Guiding Principles for Clinical Research on New Chinese Medicines
2019 Rong Y	Guiding Principles for Clinical Research on New Chinese Medicines
2019 Suqin W	not mentioned
2019 Xiaoze S	Diabetic Nephropathy Diagnosis, Diagnostic Typing and Efficacy Assessment Criteria (Trial Programme)
2019 Yangxi F	Guiding Principles for Clinical Research on New Chinese Medicines
2019 Yangxia L	Diabetic Nephropathy Diagnosis, Diagnostic Typing and Efficacy Assessment Criteria (Trial Programme)
2019 Zhixiong T	Guiding Principles for Clinical Research of New Chinese Medicines (for Trial Implementation)
2019 Gangyi C	Chinese Medicine Evidence Scoring Criteria
2020 Dandan P	Criteria for the Diagnosis, Identification and Classification of Diabetic Nephropathy and Evaluation of Therapeutic Effect.
2020 Wei H	Guiding Principles for Clinical Research of New Chinese Medicines (for Trial Implementation)
2020 Xinxin P	not mentioned
2020 Yalan H	Guiding Principles for Clinical Research of New Chinese Medicines (for Trial Implementation)
2020 Yali Z	Diabetic Nephropathy Diagnosis, Diagnostic Typing and Efficacy Assessment Criteria (Trial Programme)
2021 Bing Y	Guiding Principles for Clinical Research on New Chinese Medicines
2021 Chuanfu Z	Diabetic Nephropathy Diagnosis, Diagnostic Typing and Efficacy Assessment Criteria (Trial Programme)

2021 Cuiqing Z	not mentioned
2021 Hongye C	symptom and renal Function Indicators
2021 Huaizhi L	Chinese Guidelines for the Prevention and Control of Type 2 Diabetes Mellitus
2021 Jiang H	Guiding Principles for Clinical Research on New Chinese Medicines
2021 Jinfeng S	Guiding Principles for Clinical Research on New Chinese Medicines
2021 Xiaomei D	Guiding Principles for Clinical Research on New Chinese Medicines
2021 Ying L	symptom and renal Function Indicators
2022 An Z	symptom and renal Function Indicators
2022 Chuanyong R	Chinese Medicine Symptom Score
2022 Haitao X	Criteria for the Diagnosis, Identification and Classification of Diabetic Nephropathy and Evaluation of Therapeutic Effect.
2022 Lei Z	not mentioned
2022 Li T	Chinese Guidelines for the Clinical Diagnosis and Treatment of Diabetic Kidney Disease
2022 Meizhen L	Criteria for Diagnostic Efficacy in Chinese Medicine
2022 Ping W	Guiding Principles for Clinical Research on New Chinese Medicines
2022 Yan C	Guiding Principles for Clinical Research on New Chinese Medicines
2022 Yu Z	symptom and renal Function Indicators
2023 Duanyang J	symptom and renal Function Indicators
2023 Jiali Y	symptom and renal Function Indicators
2023 Ruixuan W	Guiding Principles for Clinical Research on New Chinese Medicines
2023 Xin Y	not mentioned
2023 Xue F	Criteria for Diagnostic Efficacy in Chinese Medicine

Table 5 Intervention of study

Study	study intervention	
	experimental group	control group
2013 Yi S	Bushen Yiqi Huoxue formula 250ml bid+Enalapril tablets po 10mg qd	Enalapril tablets po 10mg qd
2014 Angui J	Guizhi Fuling formula100ml bid+Enalapril Maleate tablets5 mg qd	Enalapril Maleate tablets po 5mg qd
2014 Liping T	Jiangtang Yishen formula 100ml bid	routine treatment
2014 Yalian H	Buyuan Huoxue formula 150ml qd+Epalrestat tablets po 150mg qd	Epalrestat tablets po 150mg qd
2015 Bailong C	Bushen Huayu Tangshen formula 200ml bid	routine treatment
2015 Beide S	Yiqi Yangyin Huoxue formula 250ml bid	routine treatment
2015 Jialing Y	Jiangtang Baoshen formula 100ml bid	routine treatment
2015 Jianmin Y	Huoxue Yishen formula qd	routine treatment
2015 Jingjing H	Shenqi Dihuang formula100ml bid+Valsartan capsules po 80mg qd	Valsartan capsules po 80mg qd
2015 Ming L	Shenqi Dihuang formula 150ml bid	routine treatment
2015 Ting Z	Wenshen Jianpi Huayu Tongluo formula 100ml bid+Fosinopril po 10mg qd	Fosinopril po 10mg qd
2016 Lige L	Shenqi Dihuang formula plus Taohe Chengqi formula formula bid+Irbesartan Tablets 150mg qd	Irbesartan tablets po 150mg qd
2016 Shulan W	Zhenwu formula 50ml tid	routine treatment
2016 Tao L	Yiqi Bushen formula100ml bid+ Timosartan tablets po 20mg qd	Timosartan tablets po 20mg qd
2016 Xiaojing D	Tangshen Qingxuan formula 150ml bid+Benadryl tablets po 10mg qd	Benazepril tablets po 10mg qd
2017 Ni Z	Self-formulated formula+Timosartan tablets po 80mg qd	Timosartan tablets po 80mg qd
2017 Ping D	Shenqi Dihuang formula 200ml tid	routine treatment
2017 Rucui Y	Yiqi Yangyin Tongluo formula bid	routine treatment
2017 Xiaoli Z	Yiqi Huayu formula bid	routine treatment
2018 Aimin Hu	Tangshen formula 200ml bid+Benadryl hydrochloride Benadryl tablets 10mg qd	Benazepril hydrochloride tablets po 10mg qd
2018 Huajun L	Jianpi Gushen formula bid	routine treatment

2018 Liwen P	Bushen Yiqi Tongluo Huazhuo formula 300ml bid	routine treatment
2018 Min Z	Shenqi Dihuang formula 100ml bid+Valsartan capsules 80mg qd	Valsartan capsules po 80mg qd
2018 Shijian Q	Yiqi Huoxue Gushen formula 150ml bid	routine treatment
2018 Xiaoyi Z	Yiqi Ziyin Huoxue formula bid+Timosartan tablets po 80mg bid	Timosartan tablets po 80mg bid
2018 Xingguo L	Liuwei Dihuang formula plus Shengmai formula bid	routine treatment
2019 Changsong Z	Yiqi Ziyin Huoxue Huayu formula bid +Valsartan tablets po 80mg qd	Valsartan tablets po 80mg qd
2019 Chenhui D	Yiqi Yangyin Tongluo formula 100ml bid	routine treatment
2019 Fangqiang C	Baoshenfang Formula	routine treatment
2019 Jili S	Shenqi Dihuang formula bid+Timosartan tablets po 40mg qd	routine treatment
2019 Ling W	Zishen Yiqi formula 100ml bid+Epalrestat tablets po 150mg qd	Timosartan tablets po 40mg qd
2019 Rong Y	Wentang Baoshen Xiaozheng formula 100ml bid	Epalrestat tablets po 150mg qd
2019 Suqin W	Tongzhuo Jiangzuo Yishen formula 200ml bid	routine treatment
2019 Xiaoze S	Xiaoke formula bid+Reduced glutathione injection igtt 0.3g qd	routine treatment
2019 Yangxi F	Shenqi Dihuang formula +Prostaglandin i.v 10ug qd	Reduced glutathione injection igtt 0.3g qd
2019 Yangxia L	Huoxue Yishen formula 100ml bid	Prostaglandin i.v 10ug qd
2019 Zhixiong T	Qitian formula 100ml bid+Valsartan Potassium tablets po 50mg qd	routine treatment
2019 Gangyi C	Yiqi Yangyin Quyu formula 200ml bid	Valsartan Potassium tablets po 50mg qd
2020 Dandan P	Huoxue Yangyin formula+Enalapril Maleate tablets 20-40mg qd	Enalapril Maleate tablets po 20-40mg qd
2020 Wei H	Gushen Jianpi Hhuashi formula200ml bid+Candesartan ester tablets po 4mg qd	Candesartan ester tablets po 4mg qd
2020 Xinxin P	Tongluo Digui formula 100ml bid	routine treatment
2020 Yalan H	Buyang Huanwu formula plus Sanren formula 100ml bid	Buyang Huanwu formula with Three-nuts formula 100ml bid+Valsartan Potassium tablets po 50mg qd
2020 Yali Z	Qiju Dihuang formula 200ml bid+Exenatide i.h 5-10μg bid	Exenatide i.h 5-10μg bid
2021 Bing Y	Jianpi Baoshen Tongluo formula bid+Candesartan ester tablets po 2# qd	Candesartan ester tablets po 2# qd

2021 Chuanfu Z	Tangshen formula 100ml bid	routine treatment
2021 Cuiqing Z	Shenqi Dihuang formula 100ml bid+prostaglandin iv 5ug qd	Prostaglandin iv 5ug qd
2021 Hongye C	Shenqi Dihuang formula 100ml bid+Alfacalcitol Soft capsule 0.5μg qd	Alfacalcitol Soft capsule po 0.5μg qd
2021 Huaizhi L	Shenmai Dihuang formula 150ml bid+Valsartan capsules 80mg qd	Valsartan Capsules 80mg qd
2021 Jiang H	Ginseng and Astragalus Dihuang formula bid	routine treatment
2021 Jinfeng S	Ginseng and Astragalus Dihuang formula 150ml bid	routine treatment
2021 Xiaomei D	Ginseng and Astragalus Dihuang formula 200ml bid	routine treatment
2021 Ying L	Jianshen Huayu formula 100ml tid +Prostaglandin iv 5ug qd	Prostaglandin i.v 5ug qd
2022 An Z	Shenqi Dihuang formula 167~250ml bid/tid+AstraZeneca tablets po 10mg qd	Dagliflozin tablets po 10mg qd
2022 Chuanyong R	Jianpi Yishen formula 125ml bid+Epalrestat tablets po 50mg tid	Epalrestat tablets po 50mg tid
2022 Haitao X	Jianpi Yangyin Guse formulaStrengthening the Spleen, Nourishing Yin, and Arresting Discharge foemula 150ml bid +Valsartan tablets 80~160mg qd	Valsartan tablets po 80~160mg qd
2022 Lei Z	Four Junzi formula plus Liuwei Dihuang formula 200ml bid	routine treatment
2022 Li T	Yiqi Yangyin Tongluo formula 150ml bid+Prostaglandin i.v 10ug qd	Prostaglandin i.v 10ug qd
2022 Meizhen L	Qidi Shenkang formula bid+Prostaglandin iv 10ug qd	Prostaglandin i.v 10ug qd
2022 Ping W	Jianpi Gushen formula formula bid	routine treatment
2022 Yan C	Shenqi Dihuang formula 200ml tid+Insulin i.h	Insulin i.h
2022 Yu Z	Zhenwu formula bid+Valsartan Potassium Tablets po 100mg qd	Valsartan Potassium tablets po 100mg qd
2023 Duanyang J	Shenqi Dihuang formula 150ml bid	routine treatment
2023 Jiali Y	Danggui Liuhuang formula 75ml bid+Epalrestat tablets po 150mg qd	Epalrestat tablets po 150mg qd
2023 Ruixuan W	Shenqi Dihuang formula bid	routine treatment
2023 Xin Y	Buyang Huanwu formula 150ml bid+Dagliflozin tablets po 10mg qd	Dagliflozin tablets po 10mg qd
2023 Xue F	Yishen Paidu formula 200ml bid	routine treatment

Table 6 outcomes of study

Study	efficientive rate（E/C）	adverse event(E/C)	FBG difference(mmol/l)		HbA1c difference(%)		SCR difference (μmol/L)		UAER difference (μg/min)		24-hour urine protein difference(g/24h)		BUN difference（mmol/L）	
			experimental group	control group	experimental group	control group	experimental group	control group	experimental group	control group	/	/	-BUN difference（mmol	-
2013 Yi S	90.0%/53.3%	0/0	-0.42±0.35	-0.43±0.08	-0.06	-0.1±0.01	-20.54±2.16	-4.79±1.08	/	/	-0.96±0.05	-0.34±0.11	/	/
2014 Angui J	/	0/0	-0.31±0.07	-0.3±0.2	-2.09	-2.23±3.11	-2.09±3.67	-2.23±3.11	/	/	/	/	-0.32±0.09	-0.25±0.28
2014 Liping T	87.10%/59.46%	3/4	-5.08±0.89	-3.31±1.98	/	/	-79.9±5.29	-48.12±3.62	-73.32±0.4	-66.17±6.19	-2.53±1.04	-1.51±0.62	-5.16±1.11	-1.91±0.78
2014 Yalian H	/	unmentioned	/	/	/	/	-15.47±0.91	-12.55±6.91	/	/	-0.22±0.09	-0.10±0.01	/	/
2015 Bailong C	/	unmentioned	-7.52±2.74	-7.59±2.74	-2.43	-2.18±0.19	-120.26±1.74	-84.66±1.25	/	/	-0.50±0.10	-0.31±0.05	/	/
2015 Beide S	90.7%/67.4%	0/0	-1.12±0.22	-0.33±0.10	-1	-0.11±0.33	-21.65±6.99	-6.9±8.08	-61.2±40.06	-9.36±24.18	/	/	-2.12±0.22	-0.33±0.10
2015 Jialing Y	91.67%/78.72%	0/0	-5.68±1.46	-2.94±0.99	-5.29	-2.95±2.55	-3.1±1.3	-3.4±0.4	-44.7±4.5	-19.8±3.4	/	/	-1.47±0.41	-0.84±0.58
2015 Jianmin Y	/	unmentioned	-0.32±0.05	-0.05±0.08	-0.45	0.02±0.04	-23.11±0.9	-4.69±7.3	-58.28±12.05	-34.45±8.05	/	/	/	/
2015 Jingjing H	47/38	unmentioned	-2.1±0.12	-1.2±0.07	-1.5	-1.0±0.11	-20.0±1.3	-18.8±0.5	/	/	/	/	-1.3±0.0	-1.0±0.0
2015 Ming L	80.0%/56.0%	unmentioned	-0.37±0.2	-0.18±	-1.61	-1.11±	/	/	/	/	-0.71±0.	-0.056±0.	/	/

			3	0.21		0.27					009	007		
2015 Ting Z	91.7%/76.7%	unmentioned	/	/	/	/	-20.73±3.22	-5.38±2.56	/	/	-1.11±0.11	-0.53±0.07	/	/
2016 Lige L	90.77%/67.69%	unmentioned	-3.17±0.36	-2.58±0.04	-4.1	-3.85±0.04	-7.94±2.39	-3.21±0.18	-261.06±22.42	-125.19±18.41	-1.18±0.11	-0.72±0.05	/	/
2016 Shulan W	84.3%/59.3%	2/1	-3.49±0.68	-2.91±0.47	/	/	-148.89±26.94	-70.74±10.97	/	/	-0.73±0.12	-0.42±0.09	/	/
2016 Tao L	76.59%/57.14%	unmentioned	-0.2±0.33	-0.14±0.15	-0.14	-0.04±0.08	-1.78±1.14	4.22±0.65	/	/	-1.24±0.24	0.93±0.06	/	/
2016 Xiaojing D	90.57%/79.25%	0/0	-0.76±0.89	-1.17±0.52	-1.32	-0.85±0.05	/	/	/	/	-1.03±0.27	0.48±0.03	/	/
2017 Ni Z	94.3%/71.4%	0/0	/	/	/	/	/	/	-64.49±11.18	-30.1±19.77	-0.81±0.04	0.27±0.13	/	/
2017 Ping D	85.71%/68.42%	0/0	-0.79±0.6	-0.66±0.29	-0.56	-0.31±0.33	-8.43±0.64	-8.16±1.65	/	/	-0.072±0.065	-0.042±0.09	/	/
2017 Rucui Y	84.91%/66.04%	unmentioned	-3.26±1.14	-2.52±0.78	-1.78	-0.23±0.18	/	/	/	/	-0.32±0.001	0.20±0.02	/	/
2017 Xiaoli Z	91.3%/71.4%	unmentioned	-0.29±0.23	-0.21±0.08	-1.68	-1.09±0.02	-150.7±11.8	-91.9±13.8	/	/	-0.23±0.003	-0.21±0.004	-3.59±0.12	-1.78±0.65
2018 Aimin Hu	83.5%/69%	0/0	-3.15±1.01	-1.75±0.58	-1.01	-0.47±0.07	-3.49±1.01	-4.27±4.28	/	/	-0.21±0.01	-0.18±0.003	-0.6±0.33	-0.32±0.40
2018 Huajun L	90/0%/65/0%	unmentioned	/	/	/	/	-60.9±10.19	-35.22±8.96	/	/	/	/	-3.8±0.88	-2.79±0.55
2018 Liwen P	/	unmentioned	-0.42±0.01	0.01±0.01	/	/	-8.92±8.31	-5.53±3.00	-91.34±9.98	-59.62±1.87	-0.52±0.50	-0.45±0.11	-0.34±0.05	-0.05±0.95
2018	81.6%/67.6	0/1	-	-	/	/	-	-	-	-	/	/	-1.56±0.70	-

Min Z	%		2.46±0.66	1.58±0.36			22.02±1.58	15.72±0.77	77.29±3.55	70.73±4.12				0.81±0.93
2018 Shijian Q	/	0/0	-1.24±0.42	-0.80±0.23	-1.42	-0.54±0.40	-15.04±0.63	-11.18±1.10	/	/	/	/	-2.9±0.43	-2.39±0.42
2018 Xiaoyi Z	/	/	-1.85±0.88	-1.43±0.23	/	/	-45.79±11	-22.4±7.11	-85.57±0.25	-19.13±1.15	/	/	/	/
2018 Xingguo L	98.2%/85.5%	unmentioned	-1.90±1.50	-1.73±0.98	-1.11	-1.10±0.03	/	/	/	/	-0.034±0.009	0.026±0.002	/	/
2019 Changsong Z	82.05%/61.53%	2/2	/	/	/	/	-57.5±7.3	-44.4±5.8	/	/	/	/	-4.1±1.2	-1.8±0.5
2019 Chenhui D	/	0/0	-2.21±0.85	-2.12±0.54	-2.6	-2.25±0.14	-4.43±0.64	-4.75±0.26	-84.78±13.29	-60.32±12.04	/	/	0.19±0.03	0.32±0.09
2019 Fangqiang C	84.81%/70.89%	/	/	/	/	/	-30±0.001	-6±0.001	/	/	-0.73±0.43	-0.22±0.04	-2±0.4	-0.8±0.1
2019 Jili S	94.59%/71.48%	unmentioned	/	/	/	/	-136.08±14.67	-107.78±11.51	/	/	-0.62±0.05	-0.34±0.04	-7.02±0.87	-5.04±0.65
2019 Ling W	91.1%/78.6%	/	/	/	/	/	/	/	-0.5±0.21	-0.17±0.08	/	/	/	/
2019 Rong Y	/	unmentioned	/	/	/	/	-40.83±6.68	-25.04±4.07	-71.29±11.95	-32.18±7.26	-2.34±0.85	-1.44±0.50	-5.79±0.71	-3.79±0.48
2019 Suqin W	83.33%/62.90%	unmentioned	-4.2±1.22	-3.81±1.37	-2.57	-1.81±0.76	-62.38±44.08	-31.91±8.46	/	/	-0.62±0.16	-0.27±0.01	/	/
2019 Xiaoze S	89.36%/74.47%	0/0	-2.21±2.43	-1.66±1.58	-1.83	-1.30±1.07	-24.93±3.11	-14.37±3.46	-57.17±5.67	-25.75±4.39	-0.53±0.01	-0.13±0.002	-1.04±0.16	-0.27±0.09
2019	/	0/0	-	-	/	/	-	-	/	/	-	-	-5.77±0.89	-

Yangxi F			6.05±1.35	4.53±0.39			131.2±12.29	99.73±20.67			0.61±0.18	0.42±0.09		3.65±0.42
2019 Yangxia L	35/27	unmentioned	-1.25±0.9	-1.18±0.82	-2.23	-2.1±1.15	-151.02±22.02	-110.13±17.24	/	/	-0.18±0.04	-0.07±0.04	-7.55±0.38	-4.54±0.03
2019 Zhixiong T	84.85%/71.87%	unmentioned	-1.51±0.17	-0.25±0.05	-1.32	-0.15±0.06	/	/	/	-	/	/	/	/
2019 Gangyi C	85.45%/63.64%	0/0	-0.85±0.45	-0.17±-.14	-0.68	-0.09±0.57	-15.87±8.73	0.78±0.86	-53.67±13.78	-31.21±3.38	/	/	/	/
2020 Dandan P	70.21%/44.68%	0/0	/	/	/	/	-12.9±1.49	-4.75±0.43	-78.01±11.32	-58.11±9.22	/	/	/	/
2020 Wei H	/	unmentioned	/	/	/	/	-12.98±1.57	-5.14±1.55	-56.63±11.28	-33.9±5.95	/	/	-1.88±0.81	-1.28±0.88
2020 Xinxin P	88.9%/61.1%	0/0	-5.98±1.43	-6.03±1.8	-2.51	-1.93±1.13	-95.0±42.86	-84.73±41.62	/	/	-0.73±0.44	-0.33±0.30	-5.1±2.33	-3.97±1.49
2020 Yalan H	86.11%/61.11%	1/1	-1.77±0.91	-0.75±0.31	-1.33	-0.63±0.29	-33.23±6.19	-15.66±2.82	-53.13±10.58	-21.32±4.24	/	/	-5.51±1.58	-2.07±0.49
2020 Yali Z	87.50%/71.43%	unmentioned	-3.53±1.2	-3.93±1.53	-1.5	-1.75±1.03	-16.03±1.73	-16.37±1.81	/	/	-0.45±0.12	-0.41±0.13	-1.02±0.2	-0.86±0.08
2021 Bing Y	93.3%/73.3%	unmentioned	-3.86±1.02	-2.73±1.11	-1.76	-0.92±0.68	-19.82±2.21	-13.02±1.69	-89.88±2.84	-17.63±15.50	/	/	-2.15±0.51	-1.43±0.19
2021 Chuanfu Z	/	unmentioned	/	/	/	/	-124.71±3.25	-3.59±1.53	-79.83±5.94	4.82±1.89	/	/	/	/
2021 Cuiqing Z	94.44%/83.33%	unmentioned	-2.80±0.02	-0.88±0.19	/	/	-48.02±2.63	-28.41±2.02	/	/	-0.13±0.01	-0.06±0.02	-2.9±0.02	-1.51±0.9

													1
2021 Hongye C	95.74%/80.83%	3/6	/	/	/	/	-16.2±2.36	-7.12±1.68	-39±6.12	-16.62±3.85	/	/	-2.81±0.62	-1.69±0.31
2021 Huaizhi L	59.25%/46.30%	9/10	-4.26±0.78	-2.59±0.86	/	/	-58.08±4.33	-44.79±3.36	/	/	-1.09±0.08	-0.46±0.05	/	/
2021 Jiang H	82.50%67.50%	unmentioned	-3.41±0.88	-2.78±0.63	-2.5	-1.79±0.28	-11.33±4.56	-8.58±3.55	-90.14±10.33	-48.85±6.19	/	/	-1.73±0.07	-0.87±0.11
2021 Jinfeng S	94.67%/83.56%	0/0	/	/	/	/	-84.51±11.01	-34.1±11.23	-102.08±14.78	-33.06±11.28	-0.97±0.17	-0.48±0.05	-5.42±1.44	-4.14±1.13
2021 Xiaomei D	93.88%/77.55%	unmentioned	-1.1±0.22	-1.01±0.18	-2.18	-1.8±0.08	-27.78±1.82	-19.57±1.29	/	/	-0.42±0.038	-0.197±0.031	/	/
2021 Ying L	94.34%/79.25%	6/8	/	/	/	/	-48.16±4.88	-34.53±4.16	-93.8±4.85	-68.63±1.91	/	/	-3.07±1.14	-1.69±0.96
2022 An Z	95.24%:80.95%	unmentioned	-4.27±0.03	-2.85±0.34	-4.85	-2.77±0.14	-59.57±4.75	-43.42±2.82	/	/	/	/	-7.30±1.42	-6.14±1.35
2022 Chuanyong R	88.5%/66.1%	unmentioned	/	/	/	/	-18.85±1.38	-10.92±0.38	-61.18±9.24	-42.09±6.67	/	/	/	/
2022 Haitao X	/	unmentioned	/	/	-0.5	-0.01±0.19	-36.38±26.68	-22.04±13.46	/	/	-0.88±0.19	-0.29±0.33	-2.93±1.67	-1.72±1.29
2022 Lei Z	86.05%/73.17%	unmentioned	-2.90±0.00	-1.04±0.16	/	/	-40.62±0.57	-31.09±0.69	/	/	-0.12±0.01	-0.06±0.02	-2.96±0.03	-1.64±0.01
2022 Li T	86.05%/75.97%	3/2	-2.84±0.87	-2.62±0.68	-1.18	-0.32±0.18	-65.44±4.99	-21.42±3.5	/	/	-2.15±0.39	-1.45±0.02	/	/
2022	91.67%/78.	unmenti	/	/	/	/	-	-	-	-	/	/	-3.5±0.45	-

Meizhen L	33%	oned					44.41±2.38	26.61±1.15	31.25±2.16	24.51±0.98				2.32±0.25
2022 Ping W	93.75%/77.08%	unmentioned	/	/	/	/	-139.91±11.30	-105.47±8.64	-39.84±0.42	-16.32±1.11	/	/	-5.17±0.18	-3.72±0.03
2022 Yan C	96.92%/85.25%	0/0	-1±0.47	-0.55±0.36	-1.43	-0.71±0.6	-11.54±3.06	-3.62±2.81	/	/	-0.07±0.024	-0.054±0.017	-5.11±1.2	-1.06±0.19
2022 Yu Z	96.49%:80.70%	unmentioned	/	/	/	/	-27.9±4.98	-22.38±3.81	-55.99±5.22	-32.73±4.94	-0.59±0.23	-0.32±0.22	-1.76±0.33	-0.87±0.32
2023 Duanyang J	88.89%/73.02%	unmentioned	/	/	-4.15	-3.19±0.98	-42.69±5.28	-26.85±4.21	/	/	/	/	-2.84±0.45	-1.61±0.34
2023 Jiali Y	90/91%/69.70%	unmentioned	/	/	/	/	-17.95±1.87	-13.52±1.37	/	/	-0.89±0.09	-0.65±0.06	-1.3±0.13	-0.67±0.07
2023 Ruixuan W	/	unmentioned	/	/	/	/	-95.12±30.29	-45.66±42.8	/	/	/	/	-6.17±1.83	-4.38±2.69
2023 Xin Y	93.75%/79.25%	unmentioned	-3.28±1.23	-2.15±0.82	-4.05	-2.14±0.00	-7.18±2.58	-0.64±3.02	-73.00±21.08	-36.85±21.85	/	/	/	/
2023 Xue F	/	13/10	/	/	/	/	-32.83±3.35	-25.58±1.42	/	/	-0.93±0.05	-0.58±0.05	-2.81±0.22	-1.67±0.01

E:Experimenta group; C:Control group;
FBG difference: Post-treatment Fasting Plasma Glucose minus pre-treatment Fasting Plasma Glucose;
HbA1c difference: Post-treatment Hemoglobin A1C minus pre-treatment Hemoglobin A1C;
SCR difference: Post-treatment serum creatinine minus pre-treatment serum creatinine;
UAER difference: Post-treatment urinary albumin excretion rates minus pre-treatment urinary albumin excretion rates;
24-hour urine protein difference: Post-treatment 24-hour urine protein minus pre-treatment 24-hour urine protein;
BUN difference: Post-treatment blood urea nitrogen minus pre-treatment blood urea nitrogen.

Table 7 Chinese medicine decoction prescription

group	study	Name of traditional Chinese medicine decoction
GF group	2014 Angui J	Guizhi Fuling formula
	2014 Liping T	Jiangtang Kangshen formula
YZ group	2019 Ling W	Yiqi Zishen formula
	2020 Yali Z	Qiju Dihuang formula
YH group	2017 Xiaoli Z	Yiqi Huayu formula
	2018 Aimin Hu	Tangshen formula
	2022 Chuanyong R	Jianpi Yishen formula
SD group	2019 Jili S	Shenqi Dihuang formulate
	2023 Jiali Y	Danggui Dihuang formula
BT group	2014 Yalian H	Buyuan Tongluo formula
	2019 Chenhui D	Yiqi Yangyin Tongluo formula
	2021 Cuiqing Z	Shenqi Dihuang formula
	2021 Huaizhi L	Shenmai Dihuang formula
	2021 Jiang H	Shenqi Dihuang formula
	2022 Meizhen L	Qidi Shenkang formula
	2022 Yu Z	Zhenwu formula

	2023 Ruixuan W	Shenqi Dihuang formula
	2023 Xin Y	Buyang Huanwu formula
JH group	2021 Ying L	Jianshen Huayu formula
	2023 Duanyang J	Shenqi Dihuang formulate
WJHT group	2015 Ting Z	Wenshen Jianpi Huayu Tongluo formula
	2018 Shijian Q	Yiqi Huoxue Gushen formula
	2021 Hongye C	Shenqi Dihuang formula
YYT group	2017 Rucui Y	Yiqi Yangyin Tongluo formula
	2022 Yan C	Shenqi Dihuang formula
BYTH group	2018 Liwen P	Bushen Yiqi Tongluo Huazhuo formula
	2018 Min Z	Shenqi Dihuang formula
	2021 Bing Y	Jianpi Baoshen Tongluo formula
	2022 Lei Z	Four Junzi formula plus Liuwei Dihuang formula
GJ group	2017 Ni Z	Self-formulate formula
	2020 Wei H	Gushen Jianpi formula
JB group	2015 Jialing Y	Jiangtang Baoshen formula
	2019 Gangyi C	Yiqi Yangyin Quyu formula
HY group	2015 Jingjing H	Shenqi Dihuang formula
	2019 Yangxia L	Huoxue Yishen formula

Table 8 composition of Chinese medicine decoction (Chinese romanization)

Study	Tonic	Blood-activating	Astringent	Dispelling Dampness	Heat-clearing	Laxative	Other
2013 Yi S	*Astragalus mongholicus Bunge* [Fabaceae;Astragali mongholici radix] 90g<br><br>*Eucommia ulmoides Oliv.* [Eucommiaceae;Cortex eucommiae] 30g	*Sparganium stoloniferum (Buch.-Ham. ex Graebn.)Buch.-Ham. exJuz.* [Typhaceae;Sparganii rhizoma] 30g<br><br>*Conioselinum anthriscoides 'Chuanxiong'* [Apiaceae;Chuanxiong rhizoma] 60g<br><br>*Salvia miltiorrhiza Bunge* [Lamiaceae;Radix et rhizoma salviae miltiorrhizae]	*Schisandra chinensis (Turcz.)Baill.* [Schisandraceae;Schisandrae chinensis fructus] 60g<br><br>*Rubus chingi Hu* [Rosaceae;Rubi fructus] 30g	*Plantago asiatica L.* [Plantaginaceae; Plantaginis herba] 30g<br><br>*Plantago asiatica L.* [Plantaginaceae; Plantaginis semen] 30g	*Buchoziajaponica (Thunb.)Calm.* [Rubiaceae;Buchozia japonica herba] 30g<br><br>*Cassia obtusifolia L.* [Leguminosae;Cassiae Semen] 30g<br><br>*Rehmannia glutinosa(Gaertn.) DC.* [Orobanchaceae;Radix rehmanniae] 30g<br><br>*Smilaxglabra Roxb.* [Smilacaceae;Smilacis glabrae	*Rheum oficinale Baill.* [Polygonaceae;Radix et rhizoma rhei] 30g	*Brassica juncea(L.) Czern.* [Brassicaceae;Sinapis flos] 30g

		30g<br>*Curcuma kwangsiensis S.G.Lee& C.F.Liang* [Zingiberaceae; Curcumae radix] 30g			rhizoma] 30g<br>*Scutelaria barbata D.Don* [Lamiaceae;Scutellariae barbatae herba] 30g		
2014 Angui J	*Astragalus mongholicus Bunge* [Fabaceae;Astragali mongholici radix] 45g<br>*Atractylodes macrocephala Koidz.* [Asteraceae;Atractylodis macrocephalae rhizoma] 15g<br>*Glycyrrhiza glabra L.* [Fabaceae;Glycyrrhizae radix et rhizoma] 6g	*Conioselinum anthriscoides 'Chuanxiong'* [Apiaceae;Chuanxiong rhizoma] 15g<br>*Salvia miltiorrhiza Bunge* [Lamiaceae;Radix et rhizoma salviae miltiorrhizae] 20g<br>*Prunus persica (L.)Batsch*	/	*Polyporus umbelatus(Pers.)Fries* [Polyporaceae;Polyporus] 15g<br>*Poria cocos (Schw.)Wolf* [Polyporaceae;Poria] 25g	*Buchoziajaponica (Thunb.)Calm.* [Rubiaceae;Buchozia japonica herba] 15g<br>*Paeonia × sufruticosa Andrews* [Paeoniaceae;Moutan cortex] 20g	*Rheum oficinale Baill.* [Polygonaceae;Radix et rhizoma rhei] 9g	*Cinnamomum verumJ.Presl* [Lauraceae;Cinnamomi ramulus] 15g<br>*Aconitum carmichaeli Debeaux* [Ranunculaceae;Aconiti lateralis radix preparata] 12g<br>*Cinnamomum verum J.Presl* [Lauraceae;Cinnamomi

		[Rosaceae;Persicae semen] 9g					cortex] 6g
2014 Liping T	*Astragalus mongholicus Bunge* [Fabaceae;Astragali mongholici radix] 45g, *Atractylodes macrocephala Koidz.* [Asteraceae;Atractylodis macrocephalae rhizoma] 15g, *Glycyrrhiza glabra L.* [Fabaceae;Glycyrrhizae radix et rhizoma]6g	*Conioselinum anthriscoides 'Chuanxiong'* [Apiaceae;Chuanxiong rhizoma] 15g<br>*Salvia miltiorrhiza Bunge* [Lamiaceae;Radix et rhizoma salviae miltiorrhizae] 20g<br>*Prunus persica (L.) Batsch* [Rosaceae;Persicae semen] 9g	/	*Polyporus umbelatus(Pers.)Fries* [Polyporaceae;Polyporus] 15g<br>*Poria cocos (Schw.) Wolf* [Polyporaceae;Poria] 25g	*Buchoziajaponica (Thunb.) Calm.* [Rubiaceae;Buchozia japonica herba] 15g<br>*Paeonia × sufruticosa Andrews* [Paeoniaceae;Moutan cortex] 20g	*Rheum oficinale Baill.* [Polygonaceae;Radix et rhizoma rhei] 9g	*Cinnamomum verum J.Presl* [Lauraceae;Cinnamomi ramulus] 15g<br>*Aconitum carmichaeli Debeaux* [Ranunculaceae; Aconiti lateralis radix preparata] 12g<br>*Cinnamomum verumJ.Presl* [Lauraceae;Cinnamomi cortex] 7g

2014 Yalian H	*Atractylodes macrocephala Koidz.* [Asteraceae;Atractylodis macrocephalae rhizoma] 10g<br><br>*Angelica sinensis (Oliv.) Diels* [Apiaceae;Angelicae sinensis radix] 10g, *Lyciumbarbarum L.* [Solanaceae;Fructus lycii] 10g<br><br>*Eclipta prostrata(L.)L.* [Asteraceae;Ecliptae herba] 15g<br><br>*Ligustrumlucidum W.T.Aiton* [Oleaceae;Fructus ligustri lucidi]10g<br><br>*Dioscorea oppositifolia L.* [Dioscoreaceae;Dioscor eae rhizoma] 20g	*Salvia miltiorrhiza Bunge* [Lamiaceae;Radix et rhizoma salviae miltiorrhizae] 15g<br><br>*Leonurus japonicus Hout.* [Lamiaceae;Leonuri herba] 15g	*Cornus oficinalis Siebold&Zucc.* [Cornaceae;Corni fructus] 10g<br><br>*Euryaleferox Salisb.* [Nymphaeaceae;Euryales semen] 30g<br><br>*Rosalaevigata Michx.* [Rosaceae;Rosae laevigatae fructus] 25g	*Poria cocos (Schw.)Wolf* [Polyporaceae;Poria] 15g<br><br>*Atractylodes lancea(Thunb.) DC.* [Asteraceae;Atractylodis lanceae rhizoma] 15g	*Rehmannia glutinosa(Gaertn.) DC.* [Orobanchaceae;Radix rehmanniae] 10g	/	/

2015 Bailon g C	*Astragalus mongholicus Bunge* [Fabaceae;Astragali mongholici radix] 20g	*Dioscorea nipponica Makino* [Dioscoreaceae; Dioscoreae nipponicae rhizoma] 30g<br>*Salvia miltiorrhiza Bunge* [Lamiaceae;Radix et rhizoma salviae miltiorrhizae] 30g<br>*Prunus persica (L.)Batsch* [Rosaceae;Persicae semen] 12g<br>*Gleditsia sinensis Lam.* [Fabaceae;Fuctu s gleditsiae] 15g	*Cornus oficinalis Siebold&Zucc.* [Cornaceae;Corni fructus] 12g<br>*Rosalaevigata Michx.* [Rosaceae;Rosae laevigatae fructus] 9g<br>*Euryaleferox Salisb.* [Nymphaeaceae;Euryales semen] 15g	*Atractylodes lancea(Thunb.) DC.* [Asteraceae;Atractylodis lanceae rhizoma] 15g	*Rehmannia glutinosa(Gaertn.) DC.* [Orobanchaceae;Radix rehmanniae] 15g<br>*Smilaxglabra Roxb.* [Smilacaceae;Smilacis glabrae rhizoma] 30g	*Rheum oficinale Baill.* [Polygonaceae;Radix et rhizoma rhei] 9g	*Sargassum palidum(Turn.)C. Ag* [Sargassum;Sargassum] 15g<br>*Ostreagigas* [Thunberg;Ostreae concha]30g<br>*Citrus reticulata Blanco* [Rutaceae;Citri reticulatae pericarpium] 10g

2015 Beide S	*Astragalus mongholicus Bunge* [Fabaceae;Astragali mongholici radix] 30g<br>*Pseudostelaria heterophyla(Miq.)Pax* [Caryophyllaceae;Pseudostellariae radix] 15g<br>*Angelica sinensis (Oliv.) Diels* [Apiaceae;Angelicae sinensis radix] 10g<br>*Eucommia ulmoides Oliv.* [Eucommiaceae;Cortex eucommiae] 15g	*Salvia miltiorrhiza Bunge* [Lamiaceae;Radix et rhizoma salviae miltiorrhizae] 15g	*Cornus oficinalis Siebold&Zucc.* [Cornaceae;Corni fructus] 15g<br>*Euryaleferox Salisb.* [Nymphaeaceae;Euryales semen] 15g<br>*Rosalaevigata Michx.* [Rosaceae;Rosae laevigatae fructus] 15g	*Dioscorea coletivar. hypoglauca (Palib.) S.J.Pei& C.T.Ting* [Dioscoreaceae; Dioscoreae hypoglaucae rhizoma] 15g<br>*Plantago asiatica L.* [Plantaginaceae; Plantaginis semen] 15g	*Rehmannia glutinosa(Gaertn.) DC.* [Orobanchaceae;Radix rehmanniae] 15g	/	*Pheretima aspergilum(E. Perrier)* [Pheretima;Pheretima] 10g<br>*Cinnamomum verumJ.Presl* [Lauraceae;Cinnamomi cortex] 3g
2015 Jialing Y	*Astragalus mongholicus Bunge* [Fabaceae;Astragali mongholici radix] 30g<br>*Angelica sinensis (Oliv.) Diels* [Apiaceae;Angelicae	*Salvia miltiorrhiza Bunge* [Lamiaceae;Radix et rhizoma salviae miltiorrhizae]	*Cornus oficinalis Siebold&Zucc.* [Cornaceae;Corni fructus] 10g<br>*Euryaleferox Salisb.*	/	/	/	/

	sinensis radix] 15g<br>*Lyciumbarbarum L.* [Solanaceae;Fructus lycii] 15g<br>*Dioscorea oppositifolia L.* [Dioscoreaceae;Dioscor eae rhizoma] 20g<br>*Cuscuta chinensis Lam.* [Convolvulaceae;Cuscu tae semen] 20g	30g<br>*Whitmania pigraWhitman* [Hirudinidae;Hir udo] 6g<br>*Leonurus japonicus Hout.* [Lamiaceae;Leon uri herba] 20g	[Nymphaeaceae ;Euryales semen] 10g<br>*Rosalaevigata Michx.* [Rosaceae;Rosa e laevigatae fructus] 10g				
2015 Jianmi n Y	*Astragalus mongholicus Bunge* [Fabaceae;Astragali mongholici radix] 30g<br>*Salvia miltiorrhiza Bunge* [Lamiaceae;Radix et rhizoma salviae miltiorrhizae] 20g<br>*Paeonialactiflora Pall.* [Paeoniaceae;Paeoniae	*Conioselinum anthriscoides 'Chuanxiong'* [Apiaceae;Chua nxiong rhizoma] 9g<br>*Salvia miltiorrhiza Bunge* [Lamiaceae;Radi x et rhizoma salviae	*Cornus oficinalis Siebold&Zucc.* [Cornaceae;Corn i fructus] 23g	/	/	/	*Pheretima aspergilum(E. Perrier)* [Pheretima;Ph eretima] 9g

radix alba] 12g<br><br>*Trionyx sinensis Wiegmann* [Trionychidae;Trionycis Carapax] 15g<br><br>*Cordyceps sinensis (Berk.)Sacc.* [Clavicipitaceae;Cordyceps] 9g<br><br>*Angelica sinensis (Oliv.) Diels* [Apiaceae;Angelicae sinensis radix] 15g<br><br>*Dendrobium nobile Lindl.* [Orchidaceae;Dendrobi i caulis]15g<br><br>*Glycyrrhiza glabra L.* [Fabaceae;Glycyrrhizae radix et rhizoma]3g<br><br>*Ganodermalucidum (Leyss.ex Fr.)* Karst.[Polyporaceae;Ga	miltiorrhizae] 15g<br><br>*Carthamus tinctorius L.* [Asteraceae;Cart hamiflos] 9g

	noderma] 9g<br><br>*Ophiopogonjaponicus (Thunb.)Ker Gawl.* [Asparagaceae;Liriopis seu ophiopogonis tuber] 15g<br><br>*Dioscorea oppositifolia L.* [Dioscoreaceae;Dioscor eae rhizoma] 20g<br><br>*Rehmannia glutinosa (Gaertn.)DC.* [Orobanchaceae;Radix rehmanniae praeparata] 12g						
2015 Jingjin g H	*Astragalus mongholicus Bunge* [Fabaceae;Astragali mongholici radix] 30g<br><br>*Rehmannia glutinosa (Gaertn.)DC.* [Orobanchaceae;Radix rehmanniae	*Conioselinum anthriscoides 'Chuanxiong'* [Apiaceae;Chua nxiong rhizoma] 10g<br><br>*Salvia miltiorrhiza*	*Euryaleferox Salisb.* [Nymphaeaceae ;Euryales semen] 20g	*Alisma plantago-aquatica subsp. orientale (Sam.) Sam.* [Alismataceae;Ali smatis rhizoma] 20g	/	*Rheum oficinale Baill.* [Polygonac eae;Radix et rhizoma rhei] 15g	*Pheretima aspergilum(E. Perrier)* [Pheretima;Ph eretima] 20g<br><br>*OsDraconis(Fo ssiliaOssiaMas todi)* 20g

	praeparata] 30g<br>*Angelica sinensis (Oliv.) Diels* [Apiaceae;Angelicae sinensis radix] 20g<br>*Dioscorea oppositifolia L.* [Dioscoreaceae;Dioscor eae rhizoma]30g<br>*Eucommia ulmoides Oliv.* [Eucommiaceae;Cortex eucommiae] 20g	*Bunge* [Lamiaceae;Radi x et rhizoma salviae miltiorrhizae] 20g<br>*Carthamus tinctorius L.* [Asteraceae;Cart hamiflos] 10g<br>*Prunus persica (L.)Batsch* [Rosaceae;Persic ae semen] 20g<br>*Eupolyphaga sinensis Walker* [Corydiidae;Stel eophaga] 10g					
2015 Ming L	*Astragalus mongholicus Bunge* [Fabaceae;Astragali mongholici radix] 20g<br>*Salvia miltiorrhiza Bunge*	*Salvia miltiorrhiza Bunge* [Lamiaceae;Radi x et rhizoma salviae	*Cornus oficinalis Siebold&Zucc.* [Cornaceae;Corn i fructus] 10g<br>*Schisandra*	*Stephania tetrandra S.Moore* [Menispermacea e;Stephaniae tetrandrae radix]	*Trichosanthes kirilowiMaxim.* [Cucurbitaceae;Ra dix trichosanthis] 10g<br>*Rehmannia*	/	*Panax notoginseng (Burkil) F.H.Chen* [Araliaceae;No toginseng

	[Lamiaceae;Radix et rhizoma salviae miltiorrhizae] 10g<br><br>*Rehmannia glutinosa (Gaertn.)DC.* [Orobanchaceae;Radix rehmanniae praeparata] 15g<br><br>Adenophora triphylla (Thunb.) A.DC. [Campanulaceae;Adenophorae radix] 10g<br><br>*Dioscorea oppositifolia L.* [Dioscoreaceae;Dioscor eae rhizoma] 15g<br><br>*Ophiopogonjaponicus (Thunb.)Ker Gawl.* [Asparagaceae;Liriopis seu ophiopogonis tuber] 10g<br><br>*Glycyrrhiza glabra L.* [Fabaceae;Glycyrrhizae	miltiorrhizae] 15g	*chinensis (Turcz.)Baill.* [Schisandraceae ;Schisandrae chinensis fructus] 10g	10g<br><br>*Alisma plantago-aquatica subsp. orientale (Sam.) Sam.* [Alismataceae;Alismatis rhizoma] 10g	*glutinosa(Gaertn.) DC.* [Orobanchaceae;Radix rehmanniae] 15g	radix et rhizoma] 3g

	radix et rhizoma]6g						
2015 Ting Z	*Astragalus mongholicus Bunge* [Fabaceae;Astragali mongholici radix] 30g, *Salvia miltiorrhiza Bunge* [Lamiaceae;Radix et rhizoma salviae miltiorrhizae] 15g *Dioscorea oppositifolia L.* [Dioscoreaceae;Dioscor eae rhizoma] 30g	/	*Cornus oficinalis Siebold&Zucc.* [Cornaceae;Corn i fructus] 10g	*Poria cocos (Schw.) Wolf* [Polyporaceae;P oria] 15g *Alisma plantago-aquatica subsp. orientale (Sam.) Sam.* [Alismataceae;Ali smatis rhizoma] 10g	/	/	/
2016 Lige L	*Astragalus mongholicus Bunge* [Fabaceae;Astragali mongholici radix] 30g *Angelica sinensis (Oliv.) Diels* [Apiaceae;Angelicae sinensis radix] 15g	*Conioselinum anthriscoides 'Chuanxiong'* [Apiaceae;Chua nxiong rhizoma] 12g *Carthamus tinctorius L.* [Asteraceae;Cart	/	/	*Buchoziajaponica (Thunb.) Calm.* [Rubiaceae;Bucho zia japonica herba] 15g *Coptischinensis Franch.* [Ranunculaceae;C optidis rhizoma]	/	*Pheretima aspergilum(E. Perrier)* [Pheretima;Ph eretima] 12g *Trichosanthes kirilowi Maxim.* [Cucurbitaceae ;Fructus

		hamiflos] 9g *Prunus persica (L.) Batsch* [Rosaceae;Persicae semen] 15g			30g		trichosanthis] 30g *Pinelia ternata (Thunb.) Makino* [Araceae;Pinelliae praeparatum rhizoma] 15g
2016 Shulan W	*Astragalus mongholicus Bunge* [Fabaceae;Astragali mongholici radix] 20g *Pseudostelaria heterophyla (Miq.) Pax* [Caryophyllaceae;Pseudostellariae radix] 15g *Angelica sinensis (Oliv.) Diels* [Apiaceae;Angelicae sinensis radix] 15g *Glycyrrhiza glabra L.* [Fabaceae;Glycyrrhizae	*Salvia miltiorrhiza Bunge* [Lamiaceae;Radix et rhizoma salviae miltiorrhizae] 15g *Prunus persica (L.) Batsch* [Rosaceae;Persicae semen] 15g	*Rosa laevigata Michx.* [Rosaceae;Rosa e laevigatae fructus] 9g	*Atractylodes lancea (Thunb.) DC.* [Asteraceae;Atractylodis lanceae rhizoma] 10g	*Coptis chinensis Franch.* [Ranunculaceae;Coptidis rhizoma] 8g *Paeonia × sufruticosa Andrews* [Paeoniaceae;Moutan cortex] 9g *Rehmannia glutinosa (Gaertn.) DC.* [Orobanchaceae;Radix rehmanniae]	*Rheum oficinale Baill.* [Polygonaceae;Radix et rhizoma rhei] 9g	*Cinnamomum verum J.Presl* [Lauraceae;Cinnamomi ramulus] 10g

	radix et rhizoma]6g<br><br>*Ophiopogonjaponicus (Thunb.)Ker Gawl.* [Asparagaceae;Liriopis seu ophiopogonis tuber] 20g<br><br>*Dioscorea oppositifolia L.* [Dioscoreaceae;Dioscor eae rhizoma] 20g				10g<br><br>*Trichosanthes kirilowiMaxim.* [Cucurbitaceae;Ra dix trichosanthis] 10g<br><br>*Scrophularia ningpoensis Hemsl.* [Scrophulariaceae; Scrophulariae radix] 15g		
2016 Tao L	*Astragalus mongholicus Bunge* [Fabaceae;Astragali mongholici radix] 20g<br><br>*Salvia miltiorrhiza Bunge* [Lamiaceae;Radix et rhizoma salviae miltiorrhizae] 20g<br><br>*Atractylodes macrocephala Koidz.* [Asteraceae;Atractylodi	*Salvia miltiorrhiza Bunge* [Lamiaceae;Radi x et rhizoma salviae miltiorrhizae] 15g	/	*Poria cocos (Schw.) Wolf* [Polyporaceae;P oria] 15g<br><br>*Alisma plantago-aquatica subsp. orientale (Sam.) Sam.* [Alismataceae;Ali smatis rhizoma] 12g	/	/	*Zingiber oficinale Roscoe* [Zingiberaceae ;Rhizoma zingiberis] 15g<br><br>*Aconitum carmichaeli Debeaux* [Ranunculacea e; Aconiti lateralis radix

	s macrocephalae rhizoma] 15g<br><br>*Paeonialactiflora Pall.* [Paeoniaceae;Paeoniae radix alba] 15g			*Zea maysL.* [Poaceae;Maidis stigma] 20g			preparata] 12g
2016 Xiaojing D	*Astragalus mongholicus Bunge* [Fabaceae;Astragali mongholici radix] 30g	*Conioselinum anthriscoides 'Chuanxiong'* [Apiaceae;Chuanxiong rhizoma] 15g	*Schisandra chinensis (Turcz.)Baill.* [Schisandraceae;Schisandrae chinensis fructus] 6g	*Alisma plantago-aquatica subsp. orientale (Sam.) Sam.* [Alismataceae;Alismatis rhizoma] 12g	*Phelodendron chinense C.K.Schneid.* [Rutaceae;Phellodendri chinensis cortex] 9g<br><br>*Coptischinensis Franch.* [Ranunculaceae;Coptidis rhizoma] 9g<br><br>*Rehmannia glutinosa(Gaertn.) DC.* [Orobanchaceae;Radix rehmanniae] 15g<br><br>*Gardenia*	*Rheum oficinale Baill.* [Polygonaceae;Radix et rhizoma rhei] 3g	*Saposhnikovia divaricata (Turcz. ex Ledeb.) Schischk.* [Apiaceae;Saposhnikoviae radix] 15g<br><br>*Pueraria montana var. lobata(Wild.) Maesen& S.M.Almeida ex Sanjappa& Predeep* [Fabaceae;Pueraria radix] 20g<br><br>*Ephedra sinica*

					jasminoides J.Elis [Rubiaceae;Gardeniae fructus] 6g		*Stapf* [Ephedraceae; Ephedrae herba] 3g
2017 Ni Z	*Astragalus mongholicus Bunge* [Fabaceae;Astragali mongholici radix] 20g<br><br>*Salvia miltiorrhiza Bunge* [Lamiaceae;Radix et rhizoma salviae miltiorrhizae] 15g<br><br>*Atractylodes macrocephala Koidz.* [Asteraceae;Atractylodis macrocephalae rhizoma] 10g<br><br>*Angelica sinensis (Oliv.) Diels* [Apiaceae;Angelicae sinensis radix] 10g<br><br>*Glycyrrhiza glabra L.* [Fabaceae;Glycyrrhizae	*Conioselinum anthriscoides 'Chuanxiong'* [Apiaceae;Chuanxiong rhizoma] 10g<br><br>*Salvia miltiorrhiza Bunge* [Lamiaceae;Radix et rhizoma salviae miltiorrhizae] 15g	*Cornus oficinalis Siebold&Zucc.* [Cornaceae;Corni fructus] 10g<br><br>*Euryaleferox Salisb.* [Nymphaeaceae;Euryales semen] 10g<br><br>*Rosalaevigata Michx.* [Rosaceae;Rosae laevigatae fructus] 10g	*Poria cocos (Schw.) Wolf* [Polyporaceae;Poria] 15g	*Centela asiatica (L.) Urb.* [Apiaceae;Centellae asiaticae herba] 10g	/	/

	radix et rhizoma]6g<br><br>*Dioscorea oppositifolia L.* [Dioscoreaceae;Dioscor eae rhizoma]10g<br><br>*Cuscuta chinensis Lam.* [Convolvulaceae;Cuscu tae semen] 10g<br><br>*Epimedium sagitatum (Siebold&Zucc.) Maxim.* [Berberidaceae;Epimed ii folium] 15g						
2017 Ping D	*Astragalus mongholicus Bunge* [Fabaceae;Astragali mongholici radix] 30g<br><br>*Salvia miltiorrhiza Bunge* [Lamiaceae;Radix et rhizoma salviae miltiorrhizae] 30g<br><br>*Atractylodes*	*Salvia miltiorrhiza Bunge* [Lamiaceae;Radi x et rhizoma salviae miltiorrhizae] 15g	*Rosalaevigata Michx.* [Rosaceae;Rosa e laevigatae fructus] 20g<br><br>*Euryaleferox Salisb.* [Nymphaeaceae ;Euryales semen] 20g	/	/	/	*Citrus reticulata Blanco* [Rutaceae;Citri reticulatae pericarpium] 15g

macrocephala Koidz.
[Asteraceae;Atractylodi
s macrocephalae
rhizoma] 20g

*Angelica sinensis (Oliv.)
Diels*
[Apiaceae;Angelicae
sinensis radix] 15g

Glycyrrhiza glabra L.
[Fabaceae;Glycyrrhizae

radix et
rhizoma]20g,*Ligustrum
lucidumW.T.Aiton*
[Oleaceae;Fructus
ligustri lucidi]30g

*Epimedium sagitatum
(Siebold&Zucc.)
Maxim.*
[Berberidaceae;Epimed ii
folium] 12g

2017 Rucui Y	*Astragalus mongholicus Bunge* [Fabaceae;Astragali mongholici radix] 30g<br><br>*Pseudostelaria heterophyla(Miq.)Pax* [Caryophyllaceae;Pseudostellariae radix] 24g<br><br>*Ophiopogonjaponicus (Thunb.)Ker Gawl.* [Asparagaceae;Liriopis seu ophiopogonis tuber] 10g<br><br>*Dioscorea oppositifolia L.* [Dioscoreaceae;Dioscor eae rhizoma]15g	*Salvia miltiorrhiza Bunge* [Lamiaceae;Radix et rhizoma salviae miltiorrhizae] 15g<br><br>*Carthamus tinctorius L.* [Asteraceae;Carthamiflos] 10g	*Cornus oficinalis Siebold&Zucc.* [Cornaceae;Corn i fructus] 12g	/	*Coptischinensis Franch.* [Ranunculaceae;Coptidis rhizoma] 3g<br><br>*Rehmannia glutinosa(Gaertn.) DC.* [Orobanchaceae;Radix rehmanniae] 15g<br><br>*Trichosanthes kirilowiMaxim.* [Cucurbitaceae;Radix trichosanthis] 10g	/	/
2017 Xiaoli Z	*Astragalus mongholicus Bunge* [Fabaceae;Astragali mongholici radix] 20g, *Salvia miltiorrhiza Bunge* [Lamiaceae;Radix et	*Salvia miltiorrhiza Bunge* [Lamiaceae;Radix et rhizoma salviae miltiorrhizae]	*Cornus oficinalis Siebold&Zucc.* [Cornaceae;Corn i fructus] 10<br><br>*Schisandra*	*Plantago asiatica L.* [Plantaginaceae; Plantaginis semen] 10g<br><br>*Alisma*	*Rehmannia glutinosa(Gaertn.) DC.* [Orobanchaceae;Radix rehmanniae] 10g	/	*Pheretima aspergilum(E. Perrier)* [Pheretima;Pheretima] 10g<br><br>*Panax*

rhizoma salviae miltiorrhizae] 15g<br><br>*Glehnia litoralis (A. Gray) F. Schmidt ex Miq.* [Apiaceae;Glehniae radix] 10g<br><br>*Ophiopogon japonicus (Thunb.) Ker Gawl.* [Asparagaceae;Liriopis seu ophiopogonis tuber] 10g<br><br>*Adenophora triphyla (Thunb.) A. DC.* [Campanulaceae;Adenophorae radix] 10g<br><br>*Dioscorea oppositifolia L.* [Dioscoreaceae;Dioscoreae rhizoma] 10g	10g	*chinensis (Turcz.) Bail.* [Schisandraceae;Schisandrae chinensis fructus] 10g	*plantago-aquatica subsp. orientale (Sam.) Sam.* [Alismataceae;Alismatis rhizoma] 10g	*Trichosanthes kirilowi Maxim.* [Cucurbitaceae;Radix trichosanthis] 10g		*notoginseng (Burkil) F.H.Chen* [Araliaceae;Notoginseng radix et rhizoma] 3g

2018 Aimin Hu	*Astragalus mongholicus Bunge* [Fabaceae;Astragali mongholici radix] 30g *Panaxquinquefolius L. [Araliaceae;Panacis* quinquefolii radix] 10g *Rehmannia glutinosa (Gaertn.)DC.* [Orobanchaceae;Radix rehmanniae praeparata] 15g, *Glycyrrhiza glabra L.* [Fabaceae;Glycyrrhizae radix et rhizoma]5g, *Lyciumbarbarum L.* [Solanaceae;Fructus lycii] 30g, *Polygonatum sibiricum Redouté* [Asparagaceae;Polygon ati rhizoma] 30g,	*Salvia miltiorrhiza Bunge* [Lamiaceae;Radi x et rhizoma salviae miltiorrhizae] 20g *Carthamus tinctorius L.* [Asteraceae;Cart hamiflos] 3g, Spatholobus suberectus Dunn [Fabaceae;Spath olobi caulis] 25g	*Rosalaevigata Michx.* [Rosaceae;Rosa e laevigatae fructus] 20g *Phylolobium chinense Fisch.* [Fabaceae;Astra gali complanati semen] 15g	*Plantago asiatica L.* [Plantaginaceae; Plantaginis semen] 15g *Poria cocos (Schw.)Wolf* [Polyporaceae;P oria] 20g	*Rehmannia glutinosa(Gaertn.) DC.* [Orobanchaceae;R adix rehmanniae] 20g	/	*Pheretima aspergilum(E. Perrier)* [Pheretima;Ph eretima] 10g *Panax notoginseng (Burkil) F.H.Chen* [Araliaceae;No toginseng radix et rhizoma] 4g

2018 Huajun L	*Astragalus mongholicus Bunge* [Fabaceae;Astragali mongholici radix] 30g<br><br>*Pseudostelaria heterophyla(Miq.)Pax* [Caryophyllaceae;Pseudostellariae radix] 20g<br><br>*Dioscorea oppositifolia L.* [Dioscoreaceae;Dioscoreae rhizoma]10g	/	*Cornus oficinalis Siebold&Zucc.* [Cornaceae;Corni fructus] 10g<br><br>*Schisandra chinensis (Turcz.)Baill.* [Schisandraceae;Schisandrae chinensis fructus] 20g	*Poria cocos (Schw.)Wolf* [Polyporaceae;Poria] 10g	*Buchoziajaponica (Thunb.)Calm.* [Rubiaceae;Buchozia japonica herba] 10g<br><br>*Rehmannia glutinosa(Gaertn.) DC.* [Orobanchaceae;Radix rehmanniae] 10g<br><br>*Paeonia × sufruticosa Andrews* [Paeoniaceae;Moutan cortex] 10g	/	*Panax notoginseng (Burkil) F.H.Chen* [Araliaceae;Notoginseng radix et rhizoma] 10g
2018 Liwen P	*Astragalus mongholicus Bunge* [Fabaceae;Astragali mongholici radix] 15g<br><br>*Salvia miltiorrhiza Bunge* [Lamiaceae;Radix et rhizoma salviae	*Salvia miltiorrhiza Bunge* [Lamiaceae;Radix et rhizoma salviae miltiorrhizae] 10g	*Rosalaevigata Michx.* [Rosaceae;Rosae laevigatae fructus] 15g<br><br>*Euryaleferox Salisb.* [Nymphaeaceae	*Poria cocos (Schw.)Wolf* [Polyporaceae;Poria] 10g	/	*Rheum oficinale Baill.* [Polygonaceae;Radix et rhizoma rhei] 1g	/

	miltiorrhizae] 30g<br><br>*Atractylodes macrocephala Koidz.* [Asteraceae;Atractylodis macrocephalae rhizoma] 10g<br><br>*Angelica sinensis (Oliv.) Diels* [Apiaceae;Angelicae sinensis radix] 10g<br><br>*Cuscuta chinensis Lam.* [Convolvulaceae;Cuscutae semen] 12g		;Euryales semen] 15g				
2018 Min Z	*Astragalus mongholicus Bunge* [Fabaceae;Astragali mongholici radix] 30g<br><br>*Eucommia ulmoides Oliv.* [Eucommiaceae;Cortex eucommiae] 30g	*Whitmania pigra Whitman* [Hirudinidae;Hirudo] 10g<br><br>*Prunus persica (L.) Batsch* [Rosaceae;Persicae semen] 15g	/	*Alisma plantago-aquatica subsp. orientale (Sam.) Sam.* [Alismataceae;Alismatis rhizoma] 20g	/	*Rheum oficinale Baill.* [Polygonaceae;Radix et rhizoma rhei] 10g	/

2018 Shijian Q	*Astragalus mongholicus Bunge* [Fabaceae;Astragali mongholici radix] 30g<br><br>*Salvia miltiorrhiza Bunge* [Lamiaceae;Radix et rhizoma salviae miltiorrhizae] 15g<br><br>*Dioscorea oppositifolia L.* [Dioscoreaceae;Dioscor eae rhizoma] 30g	/	*Cornus oficinalis Siebold&Zucc.* [Cornaceae;Corn i fructus] 10g	*Poria cocos (Schw.)Wolf* [Polyporaceae;P oria] 15g<br><br>*Alisma plantago-aquatica subsp. orientale (Sam.) Sam.* [Alismataceae;Ali smatis rhizoma] 10g	*Paeonia × sufruticosa Andrews* [Paeoniaceae;Mou tan cortex] 15g<br><br>*Rehmannia glutinosa(Gaertn.) DC.* [Orobanchaceae;R adix rehmanniae] 16g	/	/
2018 Xiaoyi Z	*Astragalus mongholicus Bunge* [Fabaceae;Astragali mongholici radix] 30g<br><br>Nanhaia speciosa (Champ. ex Benth.) J.Compton & Schrire [Fabaceae;Radix Millettiae Speciosae] 10g	*Whitmania pigraWhitman* [Hirudinidae;Hir udo] 5g	/	/	*Coptischinensis Franch.* [Ranunculaceae;C optidis rhizoma] 5g	*Rheum oficinale Baill.* [Polygonac eae;Radix et rhizoma rhei] 5g	/

	Alpin ia oxyphylla Miq. [Zingiberaceae;Alpiniae oxyphyllae fructus] 15g						
2018 Xingg uo L	/	*Conioselinum anthriscoides 'Chuanxiong'* [Apiaceae;Chua nxiong rhizoma] 15g<br><br>*Salvia miltiorrhiza Bunge* [Lamiaceae;Radi x et rhizoma salviae miltiorrhizae] 10g	*Rosalaevigata Michx.* [Rosaceae;Rosa e laevigatae fructus] 10g<br><br>*Euryaleferox Salisb.* [Nymphaeaceae ;Euryales semen] 12g	*Poria cocos (Schw.) Wolf* [Polyporaceae;P oria] 20g<br><br>*Wurfbainia vilosa(Lour.) Škorničk. & A.D. Poulsen* [Zingiberaceae;A momi fructus] 6g	/	/	*Panax notoginseng (Burkil) F.H.Chen* [Araliaceae;No toginseng radix et rhizoma] 3g<br><br>Gallus gallus domesticus [Brisson;Galli Gigeriae Endothelium Corneum] 10g
2019 Chang song Z	*Astragalus mongholicus Bunge* [Fabaceae;Astragali mongholici radix] 30g<br><br>Gynochthodes officinalis (F.C.How) Razafim. & B.Bremer	*Salvia miltiorrhiza Bunge* [Lamiaceae;Radi x et rhizoma salviae miltiorrhizae]	*Cornus oficinalis Siebold&Zucc.* [Cornaceae;Corn i fructus] 15g	*Poria cocos (Schw.) Wolf* [Polyporaceae;P oria] 20g<br><br>*Alisma plantago-aquatica subsp.*	*Paeonia × sufruticosa Andrews* [Paeoniaceae;Mou tan cortex] 10g<br><br>*Rehmannia glutinosa(Gaertn.)*	*Rheum oficinale Baill.* [Polygonac eae;Radix et rhizoma rhei] 15g	Styphnolobiu m japonicum (L.) Schott [Fabaceae;Sop horae flos et flos immaturus]

	[Rubiaceae;Morindae officinalis radix] 10g<br>*Dioscorea oppositifolia L.* [Dioscoreaceae;Dioscor eae rhizoma] 15g	15g<br>*Carthamus tinctorius L.* [Asteraceae;Cart hamiflos] 6g		*orientale (Sam.) Sam.* [Alismataceae;Ali smatis rhizoma] 10g	*DC.* [Orobanchaceae;R adix rehmanniae] 15g		10g
2019 Chenh ui D	*Astragalus mongholicus Bunge* [Fabaceae;Astragali mongholici radix] 20g<br>*Salvia miltiorrhiza Bunge* [Lamiaceae;Radix et rhizoma salviae miltiorrhizae] 20g<br>*Rehmannia glutinosa (Gaertn.) DC.* [Orobanchaceae;Radix rehmanniae praeparata] 20g<br>*Angelica sinensis (Oliv.) Diels* [Apiaceae;Angelicae	*Conioselinum anthriscoides 'Chuanxiong'* [Apiaceae;Chua nxiong rhizoma] 15g<br>*Salvia miltiorrhiza Bunge* [Lamiaceae;Radi x et rhizoma salviae miltiorrhizae] 15g<br>*Achyranthes bidentata Blume* [Amaranthaceae ;Achyranthis	*Cornus oficinalis Siebold&Zucc.* [Cornaceae;Corn i fructus] 15g	*Poria cocos (Schw.) Wolf* [Polyporaceae;P oria] 10g<br>*Alisma plantago-aquatica subsp. orientale (Sam.) Sam.* [Alismataceae;Ali smatis rhizoma] 10g	*Paeonia × sufruticosa Andrews* [Paeoniaceae;Mou tan cortex] 12g	/	/

	sinensis radix] 15g<br>*Dioscorea oppositifolia L.* [Dioscoreaceae;Dioscor eae rhizoma]30g	bidentatae radix] 15g					
2019 Jili S	*Astragalus mongholicus Bunge* [Fabaceae;Astragali mongholici radix] 30g<br>*Salvia miltiorrhiza Bunge* [Lamiaceae;Radix et rhizoma salviae miltiorrhizae] 15g<br>*Dioscorea oppositifolia L.* [Dioscoreaceae;Dioscor eae rhizoma]15g<br>*Ophiopogonjaponicus (Thunb.)Ker Gawl.* [Asparagaceae;Liriopis seu ophiopogonis tuber] 10g	/	*Cornus oficinalis Siebold&Zucc.* [Cornaceae;Corn i fructus] 10g	*Poria cocos (Schw.)Wolf* [Polyporaceae;P oria] 12g<br>*Alisma plantago-aquatica subsp. orientale (Sam.) Sam.* [Alismataceae;Ali smatis rhizoma] 15g	*Rehmannia glutinosa(Gaertn.) DC.* [Orobanchaceae;R adix rehmanniae] 15g<br>*Trichosanthes kirilowiMaxim.* [Cucurbitaceae;Ra dix trichosanthis] 10g<br>*Paeonia × sufruticosa Andrews* [Paeoniaceae;Mou tan cortex] 9g	/	*Pueraria montana var. lobata(Wild.) Maesen& S.M.Almeida ex Sanjappa& Predeep* [Fabaceae;Pue raria radix] 10g

2019 Ling W	*Astragalus mongholicus Bunge* [Fabaceae;Astragali mongholici radix] 25g<br><br>*Salvia miltiorrhiza Bunge* [Lamiaceae;Radix et rhizoma salviae miltiorrhizae] 20g<br><br>*Rehmannia glutinosa (Gaertn.)DC.* [Orobanchaceae;Radix rehmanniae praeparata] 20g	*Carthamus tinctorius L.* [Asteraceae;Cart hamiflos] 10g<br><br>*Prunus persica (L.)Batsch* [Rosaceae;Persic ae semen] 10g	/	*Poria cocos (Schw.)Wolf* [Polyporaceae;P oria] 15g<br><br>*Plantago asiatica L.* [Plantaginaceae; Plantaginis semen] 15g	*Scleromitrion difusum(Wild.) R.J.Wang* [Rubiaceae;Oldenl andiae diffusae herba] 10g	/	*Bupleurum chinense DC.* [Apiaceae;Bupl euri radix] 10g
2019 Rong Y	*Astragalus mongholicus Bunge* [Fabaceae;Astragali mongholici radix] 20g<br><br>*Salvia miltiorrhiza Bunge* [Lamiaceae;Radix et rhizoma salviae miltiorrhizae] 20g	*Salvia miltiorrhiza Bunge* [Lamiaceae;Radi x et rhizoma salviae miltiorrhizae] 20g<br><br>*Euonymus alatus(Thunb.)*	*Cornus oficinalis Siebold&Zucc.* [Cornaceae;Corn i fructus] 15g	*Poria cocos (Schw.)Wolf* [Polyporaceae;P oria] 10g, *Alisma plantago-aquatica subsp. orientale (Sam.) Sam.* [Alismataceae;Ali smatis rhizoma]	*Paeonia × sufruticosa Andrews* [Paeoniaceae;Mou tan cortex] 10g<br><br>*Centela asiatica (L.)Urb.* [Apiaceae;Centella e asiaticae herba]	/	*Cryptotympan apustulata* [Fabricius;Cica dae periostracum] 6g

	Rehmannia glutinosa (Gaertn.) DC. [Orobanchaceae;Radix rehmanniae praeparata] 15g<br><br>*Dioscorea oppositifolia L.* [Dioscoreaceae;Dioscor eae rhizoma] 15g	*Siebold* [Celastraceae;Eu onymilignum suberalatum] 15g		10g	15g		
2019 Suqin W	*Astragalus mongholicus Bunge* [Fabaceae;Astragali mongholici radix] 50g<br><br>*Polygonatum sibiricum Redouté* [Asparagaceae;Polygon ati rhizoma] 20g	*Salvia miltiorrhiza Bunge* [Lamiaceae;Radi x et rhizoma salviae miltiorrhizae] 30g<br><br>*Whitmania pigra Whitman* [Hirudinidae;Hir udo] 3g<br><br>*Leonurus japonicus Hout.* [Lamiaceae;Leon	*Cornus oficinalis Siebold&Zucc.* [Cornaceae;Corn i fructus] 10g	/	*Scleromitrion difusum (Wild.) R.J.Wang* [Rubiaceae;Oldenl andiae diffusae herba] 30g	*Rheum oficinale Baill.* [Polygonac eae;Radix et rhizoma rhei] 6g	*Bupleurum chinense DC.* [Apiaceae;Bupl euri radix]10g<br><br>*Pueraria montana var. lobata (Wild.) Maesen& S.M.Almeida ex Sanjappa& Predeep* [Fabaceae;Pue raria radix] 10g<br><br>*Pheretima*

		uri herba] 30g					*aspergilum(E. Perrier)* [Pheretima;Pheretima] 6g
2019 Xiaoze S	*Astragalus mongholicus Bunge* [Fabaceae;Astragali mongholici radix] 30g<br>*Atractylodes macrocephala Koidz.* [Asteraceae;Atractylodis macrocephalae rhizoma] 12g<br>*Rehmannia glutinosa (Gaertn.)DC.* [Orobanchaceae;Radix rehmanniae praeparata] 15g<br>*Culen corylifolium(L.) Medik.* [Fabaceae;Psoraleae fructus]12g<br>*Lyciumbarbarum L.* [Solanaceae;Fructus	*Conioselinum anthriscoides 'Chuanxiong'* [Apiaceae;Chuanxiong rhizoma] 12g<br>*Salvia miltiorrhiza Bunge* [Lamiaceae;Radix et rhizoma salviae miltiorrhizae] 15g<br>*Achyranthes bidentata Blume* [Amaranthaceae;Achyranthis bidentatae radix] 15g	*Cornus oficinalis Siebold&Zucc.* [Cornaceae;Corni fructus] 12g	/	*Coptischinensis Franch.* [Ranunculaceae;Coptidis rhizoma] 15g, *Trichosanthes kirilowiMaxim.* [Cucurbitaceae;Radix trichosanthis] 15g	/	*Pheretima aspergilum(E. Perrier)* [Pheretima;Pheretima] 9g

	lycii] 9g<br>*Dioscorea oppositifolia L.* [Dioscoreaceae;Dioscor eae rhizoma]9g<br>*Dendrobium nobile Lindl.* [Orchidaceae;Dendrobi i caulis] 9g						
2019 Yangxi F	*Astragalus mongholicus Bunge* [Fabaceae;Astragali mongholici radix] 30g<br>*Panax ginseng C.A.Mey.* [Araliaceae;Ginseng radix] 9g<br>*Ophiopogonjaponicus (Thunb.)Ker Gawl.* [Asparagaceae;Liriopis seu ophiopogonis tuber] 20g<br>*Dioscorea oppositifolia*	*Achyranthes bidentata Blume* [Amaranthaceae ;Achyranthis bidentatae radix] 15g	*Schisandra chinensis (Turcz.)Baill.* [Schisandraceae ;Schisandrae chinensis fructus] 15g	/	*Rehmannia glutinosa(Gaertn.) DC.* [Orobanchaceae;Radix rehmanniae] 15g<br>*Paeonia × sufruticosa Andrews* [Paeoniaceae;Moutan cortex] 15g<br>*Phelodendron chinense C.K.Schneid.*	*Rheum oficinale Baill.* [Polygonaceae;Radix et rhizoma rhei] 9g	/

	L. [Dioscoreaceae;Dioscor eae rhizoma] 20g				[Rutaceae;Phellod endri chinensis cortex] 10g		
2019 Yangxia L	*Astragalus mongholicus Bunge* [Fabaceae;Astragali mongholici radix] 30g<br>*Rehmannia glutinosa (Gaertn.) DC.* [Orobanchaceae;Radix rehmanniae praeparata] 30g<br>*Angelica sinensis (Oliv.) Diels* [Apiaceae;Angelicae sinensis radix] 20g<br>*Dioscorea oppositifolia L.* [Dioscoreaceae;Dioscor eae rhizoma]30g<br>*Eucommia ulmoides Oliv.* [Eucommiaceae;Cortex	*Conioselinum anthriscoides 'Chuanxiong'* [Apiaceae;Chua nxiong rhizoma] 10g<br>*Salvia miltiorrhiza Bunge* [Lamiaceae;Radi x et rhizoma salviae miltiorrhizae] 20g<br>*Carthamus tinctorius L.* [Asteraceae;Cart hamiflos] 10g<br>*Prunus persica (L.) Batsch* [Rosaceae;Persic	*Euryaleferox Salisb.* [Nymphaeaceae ;Euryales semen] 20g	*Alisma plantago-aquatica subsp. orientale (Sam.) Sam.* [Alismataceae;Ali smatis rhizoma] 20g	/	*Rheum oficinale Baill.* [Polygonac eae;Radix et rhizoma rhei] 15g	*Pheretima aspergilum (E. Perrier)* [Pheretima;Ph eretima] 20g<br>*OsDraconis(Fo ssiliaOssiaMas todi)* 20g

	eucommiae] 20g	ae semen] 20g *Eupolyphaga sinensis Walker* [Corydiidae;Steleophaga] 10g					
2019 Zhixiong T	*Astragalus mongholicus Bunge* [Fabaceae;Astragali mongholici radix] 30g	*Rhodiola rosea L.* [Crassulaceae;Rhodiolae roseae rhizoma] 15g	/	/	/	/	/
2019gangyi C	*Astragalus mongholicus Bunge* [Fabaceae;Astragali mongholici radix] 20g *Pseudostelaria heterophyla(Miq.)Pax* [Caryophyllaceae;Pseudostellariae radix] 15g *Rehmannia glutinosa (Gaertn.)DC.* [Orobanchaceae;Radix rehmanniae	*Conioselinum anthriscoides 'Chuanxiong'* [Apiaceae;Chuanxiong rhizoma] 15g *Salvia miltiorrhiza Bunge* [Lamiaceae;Radix et rhizoma salviae miltiorrhizae]	*Cornus oficinalis Siebold&Zucc.* [Cornaceae;Corn i fructus] 15g	/	/	/	/

	praeparata] 15g	15g					
2020 Dandan P	*Astragalus mongholicus Bunge* [Fabaceae;Astragali mongholici radix] 30g<br>*Salvia miltiorrhiza Bunge* [Lamiaceae;Radix et rhizoma salviae miltiorrhizae] 15g<br>*Polygonatum sibiricum Redouté* [Asparagaceae;Polygon ati rhizoma] 20g	*Conioselinum anthriscoides 'Chuanxiong'* [Apiaceae;Chuanxiong rhizoma] 15g<br>*Salvia miltiorrhiza Bunge* [Lamiaceae;Radix et rhizoma salviae miltiorrhizae] 15g<br>*Whitmania pigraWhitman* [Hirudinidae;Hirudo] 10g	*Cornus oficinalis Siebold&Zucc.* [Cornaceae;Corn i fructus] 10g	/	*Rehmannia glutinosa(Gaertn.) DC.* [Orobanchaceae;Radix rehmanniae] 20g	/	*Pheretima aspergilum(E. Perrier)* [Pheretima;Pheretima] 10g

2020 Wei H	*Astragalus mongholicus Bunge* [Fabaceae;Astragali mongholici radix] 20g<br><br>*Salvia miltiorrhiza Bunge* [Lamiaceae;Radix et rhizoma salviae miltiorrhizae] 15g<br><br>*Atractylodes macrocephala Koidz.* [Asteraceae;Atractylodis macrocephalae rhizoma] 10g<br><br>*Angelica sinensis (Oliv.) Diels* [Apiaceae;Angelicae sinensis radix] 10g<br><br>*Glycyrrhiza glabra L.* [Fabaceae;Glycyrrhizae radix et rhizoma]6g<br><br>*Dioscorea oppositifolia L.*	*Conioselinum anthriscoides 'Chuanxiong'* [Apiaceae;Chuanxiong rhizoma] 10g<br><br>*Salvia miltiorrhiza Bunge* [Lamiaceae;Radix et rhizoma salviae miltiorrhizae] 15g	*Cornus oficinalis Siebold&Zucc.* [Cornaceae;Corni fructus] 10g, *Euryaleferox Salisb.* [Nymphaeaceae;Euryales semen] 10g<br><br>*Rosalaevigata Michx.* [Rosaceae;Rosa e laevigatae fructus] 10g	*Poria cocos (Schw.) Wolf* [Polyporaceae;Poria] 15g	*Centela asiatica (L.) Urb.* [Apiaceae;Centellae asiaticae herba] 10g	/	/

	[Dioscoreaceae;Dioscor eae rhizoma]10g<br><br>*Cuscuta chinensis Lam.* [Convolvulaceae;Cuscu tae semen] 10g<br><br>*Epimedium sagitatum (Siebold&Zucc.) Maxim.* [Berberidaceae;Epimed ii folium] 15g						
2020 Xinxin P	*Astragalus mongholicus Bunge* [Fabaceae;Astragali mongholici radix] 20g<br><br>*Rehmannia glutinosa (Gaertn.)DC.* [Orobanchaceae;Radix rehmanniae praeparata] 20g<br><br>*Angelica sinensis (Oliv.) Diels* [Apiaceae;Angelicae sinensis radix] 30g	*Whitmania pigraWhitman* [Hirudinidae;Hir udo] 12g	/	*Alisma plantago-aquatica subsp. orientale (Sam.) Sam.* [Alismataceae;Ali smatis rhizoma] 10g	/	*Rheum oficinale Baill.* [Polygonac eae;Radix et rhizoma rhei] 15g	/

	Glycyrrhiza glabra L. [Fabaceae;Glycyrrhizae radix et rhizoma]6g<br><br>*Chinemys reevesi (Gray)*[Testudinidae; Testudinis Carapax et Plastrum] 30g						
2020 Yalan H	*Astragalus mongholicus Bunge* [Fabaceae;Astragali mongholici radix] 30g<br><br>*Angelica sinensis (Oliv.) Diels* [Apiaceae;Angelicae sinensis radix] 15g<br><br>*Glycyrrhiza glabra L.* [Fabaceae;Glycyrrhizae radix et rhizoma] 6g	*Conioselinum anthriscoides 'Chuanxiong'* [Apiaceae;Chuanxiong rhizoma] 15g	/	*Coix lacryma-jobivar. ma-yuen(Rom.Caill.) Stapf* [Poaceae;Coicis semen] 30g	*Buchoziajaponica (Thunb.)Calm.* [Rubiaceae;Buchozia japonica herba] 15g<br><br>*Galiumaparine L.* [Rubiaceae;Galium aparine herba] 30g	*Rheum oficinale Baill.* [Polygonaceae;Radix et rhizoma rhei] 10g	*Panax notoginseng (Burkil) F.H.Chen* [Araliaceae;Notoginseng radix et rhizoma] 15g
2020 Yali Z	*Rehmannia glutinosa (Gaertn.)DC.* [Orobanchaceae;Radix rehmanniae	*Conioselinum anthriscoides 'Chuanxiong'* [Apiaceae;Chuanxiong rhizoma]	*Prunus mume (Siebold) Siebold&Zucc.* [Rosaceae;Fruct	*Poria cocos (Schw.)Wolf* [Polyporaceae;Poria] 12g	*Paeonia × sufruticosa Andrews* [Paeoniaceae;Mou	/	*Chrysanthemum × morifolium (Ramat.) Hemsl.*

	praeparata] 15g<br><br>*Angelica sinensis (Oliv.) Diels*<br>[Apiaceae;Angelicae sinensis radix] 12g<br><br>*Lyciumbarbarum L.*<br>[Solanaceae;Fructus lycii] 25g<br><br>*Dioscorea oppositifolia L.*<br>[Dioscoreaceae;Dioscor eae rhizoma] 12g	12g<br><br>*Salvia miltiorrhiza Bunge*<br>[Lamiaceae;Radi x et rhizoma salviae miltiorrhizae] 10g<br><br>*Carthamus tinctorius L.*<br>[Asteraceae;Cart hamiflos] 10g<br><br>*Prunus persica (L.) Batsch*<br>[Rosaceae;Persic ae semen] 10g	us mume] 10g<br><br>*Schisandra chinensis (Turcz.) Baill.*<br>[Schisandraceae ;Schisandrae chinensis fructus] 15g<br><br>*Tenodera sinensis Saussure*<br>[Mantodea;Man tidis ootheca] 10g	*Alisma plantago-aquatica subsp. orientale (Sam.) Sam.*<br>[Alismataceae;Ali smatis rhizoma] 10g	tan cortex] 10g		[Asteraceae;Ch rysanthemi flos] 12g
2021 Bing Y	*Astragalus mongholicus Bunge*<br>[Fabaceae;Astragali mongholici radix] 30g<br><br>*Panaxquinquefolius L. [Araliaceae;Panacis*	*Salvia miltiorrhiza Bunge*<br>[Lamiaceae;Radi x et rhizoma salviae miltiorrhizae]	*Cornus oficinalis Siebold&Zucc.*<br>[Cornaceae;Corn i fructus] 12g<br><br>*Rosalaevigata Michx.*	*Poria cocos (Schw.) Wolf*<br>[Polyporaceae;P oria] 15g	/	*Rheum oficinale Baill.*<br>[Polygonac eae;Radix et rhizoma rhei] 10g	/

	quinquefolii radix] 10g *Atractylodes macrocephala Koidz.* [Asteraceae;Atractylodis macrocephalae rhizoma] 12g *Rehmannia glutinosa (Gaertn.)DC.* [Orobanchaceae;Radix rehmanniae praeparata] 15g *Angelica sinensis (Oliv.) Diels* [Apiaceae;Angelicae sinensis radix] 12g *Dioscorea oppositifolia L.* [Dioscoreaceae;Dioscor eae rhizoma] 15g	30g *Whitmania pigraWhitman* [Hirudinidae;Hirudo] 6g *Lycopuslucidus Turcz. ex Benth.* [Lamiaceae;Lycopi herba] 10g	[Rosaceae;Rosae laevigatae fructus] 15g, *Euryaleferox Salisb.* [Nymphaeaceae;Euryales semen] 15g, *Schisandra chinensis (Turcz.)Bail.* [Schisandraceae;Schisandrae chinensis fructus] 12g				
2021 Chuan fu Z	*Astragalus mongholicus Bunge* [Fabaceae;Astragali mongholici radix] 30g	*Gypsophila vaccaria(L.)Sm.* [Caryophyllaceae; Vaccariae	*Euryaleferox Salisb.* [Nymphaeaceae;Euryales	*Vincetoxicum mukdenense Kitag.* [Apocynaceae;Cy	*Coptischinensis Franch.* [Ranunculaceae;Coptidis rhizoma]	*Rheum oficinale Baill.* [Polygonac	/

	Angelica sinensis (Oliv.) Diels [Apiaceae;Angelicae sinensis radix] 10g<br><br>*Trigonelafoenum-graecumL.* [Fabaceae;Foenugraeci semen] 10g<br><br>*Ganodermalucidum (Leyss.ex Fr.)* Karst.[Polyporaceae;Ganoderma] 30g	semen] 30g<br><br>*Gleditsia sinensis Lam.* [Fabaceae;Fuctu s gleditsiae] 30g	semen] 15g	nanchi paniculati radix et rhizoma] 15g	5g<br><br>*Smilaxglabra Roxb.* [Smilacaceae;Smilacis glabrae rhizoma] 30g	eae;Radix et rhizoma rhei] 30g	
2021 Cuiqing Z	*Astragalus mongholicus Bunge* [Fabaceae;Astragali mongholici radix] 20g<br><br>*Salvia miltiorrhiza Bunge* [Lamiaceae;Radix et rhizoma salviae miltiorrhizae] 20g<br><br>*Rehmannia glutinosa (Gaertn.)DC.* [Orobanchaceae;Radix	*Salvia miltiorrhiza Bunge* [Lamiaceae;Radix et rhizoma salviae miltiorrhizae] 10g	*Cornus oficinalis Siebold&Zucc.* [Cornaceae;Corn i fructus] 10g	*Poria cocos (Schw.) Wolf* [Polyporaceae;Poria] 15g<br><br>*Alisma plantago-aquatica subsp. orientale (Sam.) Sam.* [Alismataceae;Alismatis rhizoma] 10g	*Paeonia × sufruticosa Andrews* [Paeoniaceae;Moutan cortex] 10g	/	/

	rehmanniae praeparata] 10g<br><br>*Glycyrrhiza glabra L.* [Fabaceae;Glycyrrhizae radix et rhizoma]5g<br><br>*Dioscorea oppositifolia L.* [Dioscoreaceae;Dioscor eae rhizoma]15g<br><br>*Reynoutria multiflora (Thunb.)Moldenke* [Polygonaceae;Polygon i multiflori radix praeparata] 15g<br><br>*Cuscuta chinensis Lam.* [Convolvulaceae;Cuscu tae semen] 15g						
2021 Hongye C	*Astragalus mongholicus Bunge* [Fabaceae;Astragali mongholici radix] 30g<br><br>*Atractylodes macrocephala Koidz.*	/	*Cornus oficinalis Siebold&Zucc.* [Cornaceae;Corn i fructus] 10g	*Poria cocos (Schw.)Wolf* [Polyporaceae;P oria] 15g<br><br>*Alisma plantago-*	*Paeonia × sufruticosa Andrews* [Paeoniaceae;Mou tan cortex] 15g<br><br>*Rehmannia*	/	/

	[Asteraceae;Atractylodis macrocephalae rhizoma] 20g<br>*Salvia miltiorrhiza Bunge* [Lamiaceae;Radix et rhizoma salviae miltiorrhizae] 15g<br>*Dioscorea oppositifolia L.* [Dioscoreaceae;Dioscoreae rhizoma] 30g			*aquatica subsp. orientale (Sam.) Sam.* [Alismataceae;Alismatis rhizoma] 10g	*glutinosa(Gaertn.) DC.* [Orobanchaceae;Radix rehmanniae] 17g		
2021 Huaizhi L	*Pseudostelaria heterophyla(Miq.)Pax* [Caryophyllaceae;Pseudostellariae radix] 20g, *Atractylodes macrocephala Koidz.* [Asteraceae;Atractylodis macrocephalae rhizoma] 10g<br>*Glycyrrhiza glabra L.* [Fabaceae;Glycyrrhizae radix et rhizoma]6g	*Salvia miltiorrhiza Bunge* [Lamiaceae;Radix et rhizoma salviae miltiorrhizae] 10g	*Cornus oficinalis Siebold&Zucc.* [Cornaceae;Corni fructus] 15g	*Poria cocos (Schw.) Wolf* [Polyporaceae;Poria] 15g<br>*Alisma plantago-aquatica subsp. orientale (Sam.) Sam.* [Alismataceae;Alismatis rhizoma] 15g	*Paeonia × sufruticosa Andrews* [Paeoniaceae;Moutan cortex] 15g<br>*Rehmannia glutinosa(Gaertn.) DC.* [Orobanchaceae;Radix rehmanniae] 15g<br>*Scrophularia*	/	/

	Ophiopogonjaponicus (Thunb.)Ker Gawl. [Asparagaceae;Liriopis seu ophiopogonis tuber] 15g<br><br>*Dioscorea oppositifolia L.* [Dioscoreaceae;Dioscor eae rhizoma]15g				*ningpoensis Hemsl.* [Scrophulariaceae; Scrophulariae radix] 15g		
2021 Jiang H	*Astragalus mongholicus Bunge* [Fabaceae;Astragali mongholici radix] 30g<br><br>*Salvia miltiorrhiza Bunge* [Lamiaceae;Radix et rhizoma salviae miltiorrhizae] 20g<br><br>*Dioscorea oppositifolia L.* [Dioscoreaceae;Dioscor eae rhizoma]15g<br><br>*Polygonatum sibiricum*	*Conioselinum anthriscoides 'Chuanxiong'* [Apiaceae;Chua nxiong rhizoma] 15g<br><br>*Salvia miltiorrhiza Bunge* [Lamiaceae;Radi x et rhizoma salviae miltiorrhizae] 15g	*Cornus oficinalis Siebold&Zucc.* [Cornaceae;Corn i fructus] 15g	*Zea maysL.* [Poaceae;Maidis stigma] 30g<br><br>*Alisma plantago-aquatica subsp. orientale (Sam.) Sam.* [Alismataceae;Ali smatis rhizoma] 15g	*Rehmannia glutinosa(Gaertn.) DC.* [Orobanchaceae;R adix rehmanniae] 20g<br><br>*Paeonia × sufruticosa Andrews* [Paeoniaceae;Mou tan cortex] 15g	/	/

	Redouté [Asparagaceae;Polygon ati rhizoma] 15g<br>*Angelica sinensis (Oliv.) Diels* [Apiaceae;Angelicae sinensis radix] 15g						
2021 Jinfeng S	*Astragalus mongholicus Bunge* [Fabaceae;Astragali mongholici radix] 30g<br>*Salvia miltiorrhiza Bunge* [Lamiaceae;Radix et rhizoma salviae miltiorrhizae] 20g<br>*Dioscorea oppositifolia L.* [Dioscoreaceae;Dioscor eae rhizoma] 15g	*Salvia miltiorrhiza Bunge* [Lamiaceae;Radi x et rhizoma salviae miltiorrhizae] 15g	*Cornus oficinalis Siebold&Zucc.* [Cornaceae;Corn i fructus] 10g<br>*Euryaleferox Salisb.* [Nymphaeaceae ;Euryales semen] 15g<br>*Rosalaevigata Michx.* [Rosaceae;Rosa e laevigatae fructus] 15g	*Poria cocos (Schw.) Wolf* [Polyporaceae;P oria] 10g<br>*Alisma plantago-aquatica subsp. orientale (Sam.) Sam.* [Alismataceae;Ali smatis rhizoma] 10g<br>*Zea maysL.* [Poaceae;Maidis stigma] 20g<br>*Coix lacryma-jobivar. ma-*	*Rehmannia glutinosa(Gaertn.) DC.* [Orobanchaceae;R adix rehmanniae] 20g, *Centela asiatica(L.) Urb.* [Apiaceae;Centella e asiaticae herba] 15g	*Rheum oficinale Baill.* [Polygonac eae;Radix et rhizoma rhei] 10g	/

				yuen(Rom.Caill.) Stapf [Poaceae;Coicis semen] 20g			
2021 Ying L	*Astragalus mongholicus Bunge* [Fabaceae;Astragali mongholici radix] 60g<br>*Ganodermalucidum (Leyss.ex Fr.)* Karst.[Polyporaceae;Ganoderma] 30g<br>*Cuscuta chinensis Lam.* [Convolvulaceae;Cuscu tae semen] 30g	*Carthamus tinctorius L.* [Asteraceae;Cart hamiflos] 10g<br>*Ephedra sinica Stapf* [Ephedraceae;Ephedrae herba] 10g	/	*Plantago asiatica L.* [Plantaginaceae; Plantaginis semen] 20g<br>*Polyporus umbelatus(Pers.)Fries* [Polyporaceae;Polyporus] 20g	*Scleromitrion difusum(Wild.) R.J.Wang* [Rubiaceae;Oldenlandiae diffusae herba] 20g<br>*Scrophularia ningpoensis Hemsl.* [Scrophulariaceae; Scrophulariae radix] 20g	/	/
2022 An Z	*Astragalus mongholicus Bunge* [Fabaceae;Astragali mongholici radix] 45g<br>*Atractylodes macrocephala Koidz.* [Asteraceae;Atractylodi s macrocephalae	/	*Rosalaevigata Michx.* [Rosaceae;Rosa e laevigatae fructus] 10g<br>*Euryaleferox Salisb.* [Nymphaeaceae	/	*Rehmannia glutinosa(Gaertn.) DC.* [Orobanchaceae;Radix rehmanniae] 20g<br>*Centela asiatica (L.) Urb.*	/	*Cryptotympan apustulata* [Fabricius;Cicadae periostracum] 6g<br>*Saposhnikovia divaricata*

	rhizoma] 20g<br><br>*Cuscuta chinensis Lam.* [Convolvulaceae;Cuscu tae semen] 10g<br><br>*Eclipta prostrata(L.)L.* [Asteraceae;Ecliptae herba] 15g		;Euryales semen] 15g		[Apiaceae;Centella e asiaticae herba] 15g		*(Turcz. ex Ledeb.) Schischk.* [Apiaceae;Sap oshnikoviae radix] 15g<br><br>*Perila frutescens(L.) Briton* [Lamiaceae;Per illae folium] 10g
2022 Chuan yong R	*Astragalus mongholicus Bunge* [Fabaceae;Astragali mongholici radix] 30g<br><br>*Salvia miltiorrhiza Bunge* [Lamiaceae;Radix et rhizoma salviae miltiorrhizae] 30g<br><br>*Trionyx sinensis Wiegmann* [Trionychidae;Trionycis	*Lycopuslucidus Turcz. ex Benth.* [Lamiaceae;Lyco pi herba] 12g	*Cornus oficinalis Siebold&Zucc.* [Cornaceae;Corn i fructus] 12g	*Poria cocos (Schw.) Wolf* [Polyporaceae;P oria] 30g	*Paeonia × sufruticosa Andrews* [Paeoniaceae;Mou tan cortex] 15g<br><br>*Rehmannia glutinosa(Gaertn.) DC.* [Orobanchaceae;R adix rehmanniae] 15g	/	*Pheretima aspergilum(E. Perrier)* [Pheretima;Ph eretima] 6g<br><br>*Panax notoginseng (Burkil) F.H.Chen* [Araliaceae;No toginseng radix et

Carapax] 12g<br><br>*Angelica sinensis (Oliv.) Diels* [Apiaceae;Angelicae sinensis radix] 15g<br><br>*Ligustrumlucidum W.T.Aiton* [Oleaceae;Fructus ligustri lucidi]15g<br><br>*Dioscorea oppositifolia L.* [Dioscoreaceae;Dioscor eae rhizoma] 30g<br><br>*Polygonatum sibiricum Redouté* [Asparagaceae;Polygon ati rhizoma] 15g<br><br>*Ophiopogonjaponicus (Thunb.)Ker Gawl.* [Asparagaceae;Liriopis seu ophiopogonis tuber] 30g<br><br>*Eclipta prostrata(L.)L.*	rhizoma] 3g

	[Asteraceae;Ecliptae herba] 15g						
2022 Haitao X	*Astragalus mongholicus Bunge* [Fabaceae;Astragali mongholici radix] 30g<br>*Rehmannia glutinosa (Gaertn.)DC.* [Orobanchaceae;Radix rehmanniae praeparata] 30g<br>*Dioscorea oppositifolia L.* [Dioscoreaceae;Dioscor eae rhizoma] 20g	*Conioselinum anthriscoides 'Chuanxiong'* [Apiaceae;Chua nxiong rhizoma] 10g	*Cornus oficinalis Siebold&Zucc.* [Cornaceae;Corn i fructus] 10g	*Poria cocos (Schw.)Wolf* [Polyporaceae;P oria] 15g	/	*Rheum oficinale Baill.* [Polygonac eae;Radix et rhizoma rhei] 4g	*Cinnamomum verumJ.Presl* [Lauraceae;Cin namomi cortex] 8g
2022 Lei Z	*Astragalus mongholicus Bunge* [Fabaceae;Astragali mongholici radix] 15g<br>*Salvia miltiorrhiza Bunge* [Lamiaceae;Radix et rhizoma salviae	*Salvia miltiorrhiza Bunge* [Lamiaceae;Radi x et rhizoma salviae miltiorrhizae] 10g	*Rosalaevigata Michx.* [Rosaceae;Rosa e laevigatae fructus] 15g<br>*Euryaleferox Salisb.* [Nymphaeaceae	*Poria cocos (Schw.)Wolf* [Polyporaceae;P oria] 10g	/	*Rheum oficinale Baill.* [Polygonac eae;Radix et rhizoma rhei] 12g	/

	miltiorrhizae] 30g<br><br>*Atractylodes macrocephala Koidz.* [Asteraceae;Atractylodis macrocephalae rhizoma] 10g<br><br>*Angelica sinensis (Oliv.) Diels* [Apiaceae;Angelicae sinensis radix] 10g<br><br>*Cuscuta chinensis Lam.* [Convolvulaceae;Cuscutae semen] 12g		;Euryales semen] 15g				
2022 Li T	*Atractylodes macrocephala Koidz.* [Asteraceae;Atractylodis macrocephalae rhizoma] 9g	*Salvia miltiorrhiza Bunge* [Lamiaceae;Radix et rhizoma salviae miltiorrhizae] 20g<br><br>*Whitmania pigraWhitman*	*Schisandra chinensis (Turcz.)Baill.* [Schisandraceae ;Schisandrae chinensis fructus] 9g	*Poria cocos (Schw.)Wolf* [Polyporaceae;Poria] 12g	/	/	/

		[Hirudinidae;Hirudo] 6g					
2022 Meizhen L	*Pseudostelaria heterophyla(Miq.)Pax* [Caryophyllaceae;Pseudostellariae radix] 20g<br><br>*Atractylodes macrocephala Koidz.* [Asteraceae;Atractylodis macrocephalae rhizoma] 10g<br><br>*Glycyrrhiza glabra L.* [Fabaceae;Glycyrrhizae radix et rhizoma]6g, *Ophiopogonjaponicus (Thunb.)Ker Gawl.* [Asparagaceae;Liriopis seu ophiopogonis tuber] 15g<br><br>*Dioscorea oppositifolia L.* [Dioscoreaceae;Dioscor eae rhizoma] 15g	*Salvia miltiorrhiza Bunge* [Lamiaceae;Radix et rhizoma salviae miltiorrhizae] 10g	*Cornus oficinalis Siebold&Zucc.* [Cornaceae;Corn i fructus] 15g	*Poria cocos (Schw.)Wolf* [Polyporaceae;Poria] 15g<br><br>*Alisma plantago-aquatica subsp. orientale (Sam.) Sam.* [Alismataceae;Alismatis rhizoma] 15g	*Paeonia × sufruticosa Andrews* [Paeoniaceae;Moutan cortex] 15g<br><br>*Rehmannia glutinosa(Gaertn.) DC.* [Orobanchaceae;Radix rehmanniae] 15g<br><br>*Scrophularia ningpoensis Hemsl.* [Scrophulariaceae; Scrophulariae radix] 15g	/	/

2022 Ping W	*Atractylodes macrocephala Koidz.* [Asteraceae;Atractylodis macrocephalae rhizoma] 20g<br><br>*Panaxquinquefolius L. [Araliaceae;Panacis* quinquefolii radix] 30g<br><br>*Dioscorea oppositifolia L.* [Dioscoreaceae;Dioscor eae rhizoma] 20g	*Carthamus tinctorius L.* [Asteraceae;Cart hamiflos] 10g<br><br>*Prunus persica (L.)Batsch* [Rosaceae;Persic ae semen] 10g	/	*Poria cocos (Schw.)Wolf* [Polyporaceae;P oria] 15g<br><br>*Alisma plantago-aquatica subsp. orientale (Sam.) Sam.* [Alismataceae;Ali smatis rhizoma] 10g	*Buchoziajaponica (Thunb.)Calm.* [Rubiaceae;Bucho zia japonica herba] 15g	/	*Aconitum carmichaeli Debeaux* [Ranunculacea e;Aconiti lateralis radix preparata] 12g
2022 Yan C	*Astragalus mongholicus Bunge* [Fabaceae;Astragali mongholici radix] 30g<br><br>*Pseudostelaria heterophyla(Miq.)Pax* [Caryophyllaceae;Pseu dostellariae radix] 24g<br><br>*Dioscorea oppositifolia L.* [Dioscoreaceae;Dioscor	*Salvia miltiorrhiza Bunge* [Lamiaceae;Radi x et rhizoma salviae miltiorrhizae] 15g<br><br>*Carthamus tinctorius L.* [Asteraceae;Cart	*Cornus oficinalis Siebold&Zucc.* [Cornaceae;Corn i fructus] 12g	/	*Rehmannia glutinosa(Gaertn.) DC.* [Orobanchaceae;R adix rehmanniae] 15g<br><br>*Trichosanthes kirilowiMaxim.* [Cucurbitaceae;Ra dix trichosanthis] 10g	/	/

	eae rhizoma]15g<br>*Ophiopogonjaponicus (Thunb.)Ker Gawl.* [Asparagaceae;Liriopis seu ophiopogonis tuber] 10g	hamiflos] 10g			*Coptischinensis Franch.* [Ranunculaceae;Coptidis rhizoma] 3g		
2022 Yu Z	*Astragalus mongholicus Bunge* [Fabaceae;Astragali mongholici radix] 30g<br>*Salvia miltiorrhiza Bunge* [Lamiaceae;Radix et rhizoma salviae miltiorrhizae] 15g<br>*Rehmannia glutinosa (Gaertn.)DC.* [Orobanchaceae;Radix rehmanniae praeparata] 10g<br>*Dioscorea oppositifolia L.* [Dioscoreaceae;Dioscor	*Conioselinum anthriscoides 'Chuanxiong'* [Apiaceae;Chuanxiong rhizoma] 10g<br>*Salvia miltiorrhiza Bunge* [Lamiaceae;Radix et rhizoma salviae miltiorrhizae] 15g	*Cornus oficinalis Siebold&Zucc.* [Cornaceae;Corni fructus] 10g	*Poria cocos (Schw.)Wolf* [Polyporaceae;Poria] 15g<br>*Alisma plantago-aquatica subsp. orientale (Sam.) Sam.* [Alismataceae;Alismatis rhizoma] 15g<br>*Zea maysL.* [Poaceae;Maidis stigma] 30g	*Paeonia × sufruticosa Andrews* [Paeoniaceae;Moutan cortex] 8g	/	/

	eae rhizoma]12g<br><br>*Reynoutria multiflora (Thunb.)Moldenke* [Polygonaceae;Polygon i multiflori radix praeparata] 15g<br><br>*Cuscuta chinensis Lam.* [Convolvulaceae;Cuscu tae semen] 15g						
2023 Duany ang J	*Astragalus mongholicus Bunge* [Fabaceae;Astragali mongholici radix] 25g<br><br>*Rehmannia glutinosa (Gaertn.)DC.* [Orobanchaceae;Radix rehmanniae praeparata] 15g<br><br>*Angelica sinensis (Oliv.) Diels* [Apiaceae;Angelicae sinensis radix] 25g<br><br>*Dioscorea oppositifolia*	*Conioselinum anthriscoides 'Chuanxiong'* [Apiaceae;Chua nxiong rhizoma] 12g<br><br>*Salvia miltiorrhiza Bunge* [Lamiaceae;Radi x et rhizoma salviae miltiorrhizae] 12g	/	*Poria cocos (Schw.) Wolf* [Polyporaceae;P oria] 12g	*Phelodendron chinense C.K.Schneid.* [Rutaceae;Phellod endri chinensis cortex] 12g<br><br>*Coptischinensis Franch.* [Ranunculaceae;C optidis rhizoma] 12g<br><br>*Scutelaria baicalensis Georgi [Lamiaceae;Radix*	/	/

	L. [Dioscoreaceae;Dioscor eae rhizoma]12g				*scutelariae]* 12 g<br><br>*Rehmannia glutinosa(Gaertn.) DC.* [Orobanchaceae;Radix rehmanniae] 12g		
2023 Jiali Y	*Astragalus mongholicus Bunge* [Fabaceae;Astragali mongholici radix] 30g<br><br>*Panax ginseng C.A.Mey.* [Araliaceae;Ginseng radix] 15g<br><br>*Rehmannia glutinosa (Gaertn.)DC.* [Orobanchaceae;Radix rehmanniae praeparata] 15g<br><br>*Dioscorea oppositifolia L.* [Dioscoreaceae;Dioscor	/	*Cornus oficinalis Siebold&Zucc.* [Cornaceae;Corn i fructus] 10g	*Poria cocos (Schw.) Wolf* [Polyporaceae;Poria] 12g<br><br>*Alisma plantago-aquatica subsp. orientale (Sam.) Sam.* [Alismataceae;Alismatis rhizoma] 15g	*Paeonia × sufruticosa Andrews* [Paeoniaceae;Moutan cortex] 9g<br><br>*Trichosanthes kirilowiMaxim.* [Cucurbitaceae;Radix trichosanthis] 10g	/	*Pueraria montana var. lobata (Wild.) Maesen& S.M.Almeida ex Sanjappa& Predeep* [Fabaceae;Pueraria radix] 10g<br><br>*Saposhnikovia divaricata (Turcz. ex Ledeb.) Schischk.* [Apiaceae;Saposhnikoviae

	eae rhizoma] 15g						radix] 15g
2023 Ruixuan W	*Astragalus mongholicus Bunge* [Fabaceae;Astragali mongholici radix] 20g<br>*Salvia miltiorrhiza Bunge* [Lamiaceae;Radix et rhizoma salviae miltiorrhizae] 20g<br>*Atractylodes macrocephala Koidz.* [Asteraceae;Atractylodis macrocephalae rhizoma] 15g<br>*Dioscorea oppositifolia L.* [Dioscoreaceae;Dioscor eae rhizoma]30g<br>*Angelica sinensis (Oliv.) Diels* [Apiaceae;Angelicae sinensis radix] 12g	*Salvia miltiorrhiza Bunge* [Lamiaceae;Radix et rhizoma salviae miltiorrhizae] 15g	*Cornus oficinalis Siebold&Zucc.* [Cornaceae;Corni fructus] 10g	*Poria cocos (Schw.) Wolf* [Polyporaceae;Poria] 15g<br>*Alisma plantago-aquatica subsp. orientale (Sam.) Sam.* [Alismataceae;Alismatis rhizoma] 15g<br>*Buchozia japonica (Thunb.) Calm.* [Rubiaceae;Buchozia japonica herba] 15g	*Rehmannia glutinosa(Gaertn.) DC.* [Orobanchaceae;Radix rehmanniae] 10g<br>*Paeonia × sufruticosa Andrews* [Paeoniaceae;Moutan cortex] 9g<br>*Centela asiatica (L.) Urb.* [Apiaceae;Centellae asiaticae herba] 15g	/	/

2023 Xin Y	*Astragalus mongholicus Bunge* [Fabaceae;Astragali mongholici radix] 20g<br><br>*Salvia miltiorrhiza Bunge* [Lamiaceae;Radix et rhizoma salviae miltiorrhizae] 10g<br><br>*Atractylodes macrocephala Koidz.* [Asteraceae;Atractylodis macrocephalae rhizoma] 10g<br><br>*Rehmannia glutinosa (Gaertn.)DC.* [Orobanchaceae;Radix rehmanniae praeparata] 15g<br><br>*Cuscuta chinensis Lam.* [Convolvulaceae;Cuscu tae semen] 10g<br><br>*Dioscorea oppositifolia L.* [Dioscoreaceae;Dioscor eae rhizoma]15g<br><br>*Epimedium sagitatum (Siebold&Zucc.) Maxim.* [Berberidaceae;Epimed ii folium] 15g	*Conioselinum anthriscoides 'Chuanxiong'* [Apiaceae;Chuanxiong rhizoma] 12g<br><br>*Salvia miltiorrhiza Bunge* [Lamiaceae;Radix et rhizoma salviae miltiorrhizae] 15g<br><br>*Euonymus alatus(Thunb.) Siebold* [Celastraceae;Euonymilignum suberalatum] 15g<br><br>*Achyranthes bidentata Blume* [Amaranthaceae ;Achyranthis bidentatae radix] 15g	*Cornus oficinalis Siebold&Zucc.* [Cornaceae;Corn i fructus] 12g	*Poria cocos (Schw.)Wolf* [Polyporaceae;Poria] 15g	*Paeonia × sufruticosa Andrews* [Paeoniaceae;Moutan cortex] 10g	/	/

2023 Xue F	*Astragalus mongholicus Bunge* [Fabaceae;Astragali mongholici radix] 60g *Angelica sinensis (Oliv.) Diels* [Apiaceae;Angelicae sinensis radix] 10g	*Conioselinum anthriscoides 'Chuanxiong'* [Apiaceae;Chuanxiong rhizoma] 10g *Carthamus tinctorius L.* [Asteraceae;Carthamiflos] 10g *Prunus persica (L.) Batsch* [Rosaceae;Persicae semen] 10g	/	/	*Buchoziajaponica (Thunb.) Calm.* [Rubiaceae;Buchozia japonica herba] 10g	/	*Pheretima aspergilum (E. Perrier)* [Pheretima;Pheretima] 10g

Table 9 Adverse events

	expeimental group	control group
2013 Yi S	no adverse reaction	no adverse reaction
2014 Angui J	no adverse reaction	no adverse reaction
2014 Liping T	bloating 、nauseous, dysappetite, dry cough, rashes, pruritus et al. 3 cases	bloating、nauseous, dysappetite, dry cough, rashes, pruritus et al. 4 cases
2015 Beide S	no adverse reaction	no adverse reaction
2015 Jialing Y	no adverse reaction	no adverse reaction
2016 Juanjuan W	no adverse reaction	no adverse reaction
2016 Shulan W	mild diarrhoea 2 cases	mild diarrhoea1 1 case
2016 Xiaojing D	no adverse reaction	no adverse reaction
2017 Ni Z	no adverse reaction	no adverse reaction
2017 Ping D	no adverse reaction	no adverse reaction
2018 Aimin Hu	no adverse reaction	no adverse reaction
2018 Min Z	no adverse reaction	cough 1 case

2018 Shijian Q	no adverse reaction	no adverse reaction
2019 Changsong Z	Gastrointestinal symptoms 2 cases	abnormal liver function 1 case， transient hypotension 1 case
2019 Chenhui D	no adverse reaction	no adverse reaction
2019 Suqin W	no adverse reaction	no adverse reaction
2019 Xiaoze S	no adverse reaction	no adverse reaction
2019 Zhixiong T	no adverse reaction	no adverse reaction
2020 Dandan P	no adverse reaction	no adverse reaction
2020 Xinxin P	no adverse reaction	no adverse reaction
2020 Yalan H	mild diarrhoea 1 case	fatigue 1 case
2021 Hongye C	nausea and vomiting 1 case, insomnia 1 case, headache 1 case	nausea and vomiting 1 case, insomnia 2 cases, diarrhoea 1 case, headache 2 cases
2021 Huaizhi L	abnormal liver function 2 cases, Gastrointestinal symptoms 2 cases, cough 2 cases, rashes1 case, palpitation2 cases	abnormal liver function 2 cases, Gastrointestinal symptoms 3 cases, rashes 1 case, cough 3 cases, palpitation 1 case
2021 Jinfeng S	no adverse reaction	no adverse reaction
2021 Ying L	nausea and vomiting 3 cases, diarrhoea 2 cases, Redness and swelling at injection site 1 case, dizzy	nausea and vomiting 2 cases, diarrhoea 3 cases, Redness and swelling at injection site 2 cases, dizzy 1 case

	1 case	
2022 Li T	dizzy 2 cases, nausea and vomiting 1 case	dizzy1 case, nausea and vomiting 1 case
2022 Yan C	no adverse reaction	no adverse reaction
2023 Xue F	nausea and vomiting 3 cases, dysappetite 5 cases, fever 2 cases, rashes 3 cases	nausea and vomiting 4 cases, dysappetite 3 cases, fever 1 case, rashes 2 cases

Supplementary Figure 1. The figure legends are required to have the same font as the main text, 12 point normal Times New Roman, single spaced. Please use a single paragraph for each legend and prepare the figures keeping in mind the PDF layout.

2.1 Supplementary Figures

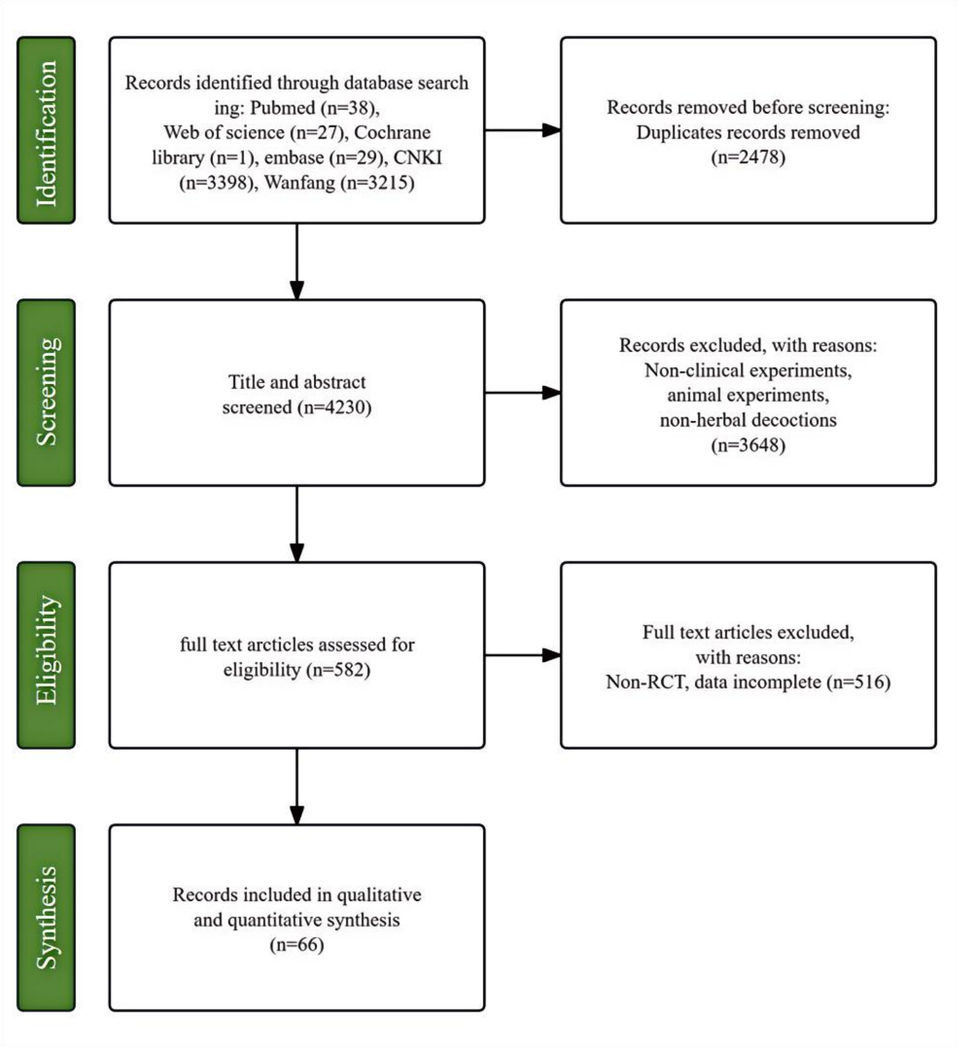

Fig. 1 Flow graph

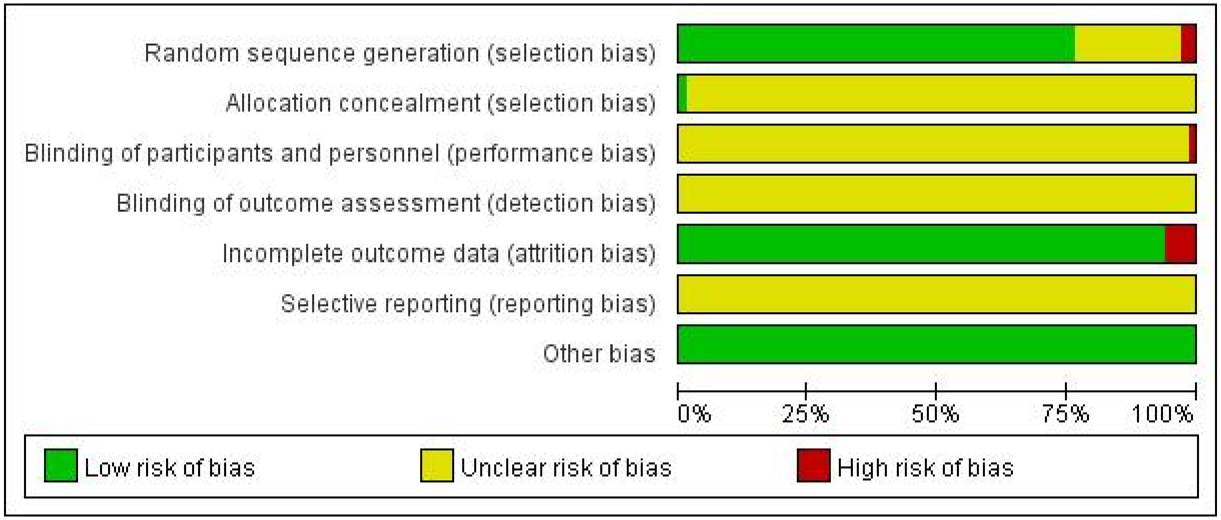

Fig. 2 Risk of bias

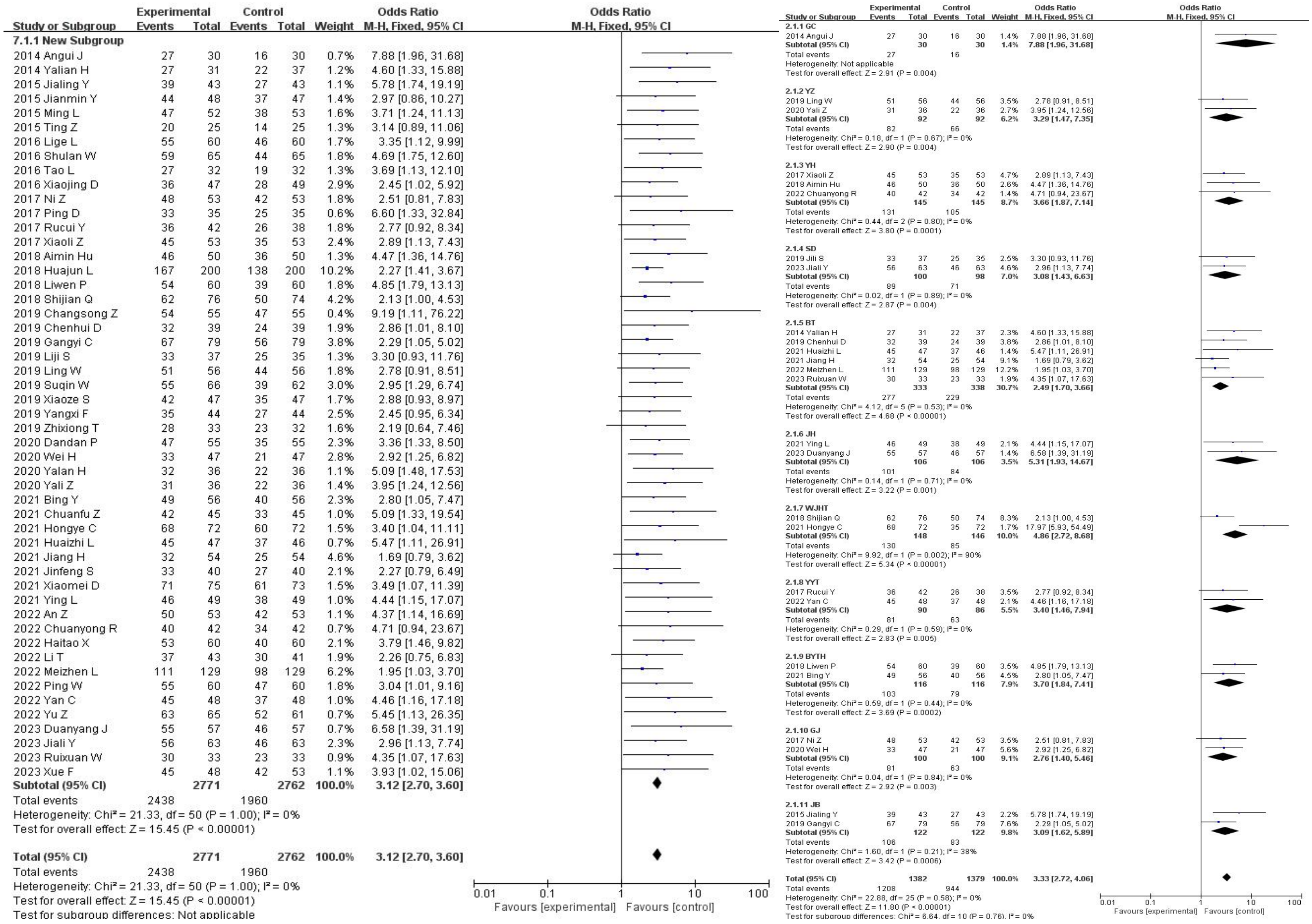

FIGURE 3 Efficacy forest plot& efficacy prescription forest plot

Study or Subgroup	Experimental Events	Experimental Total	Control Events	Control Total	Weight	Odds Ratio M-H, Fixed, 95% CI
2013 Yi S	0	52	0	52		Not estimable
2014 Angui J	0	30	0	30		Not estimable
2014 Liping T	3	39	4	39	9.5%	0.73 [0.15, 3.50]
2015 Beide S	0	89	0	89		Not estimable
2015 Jialing Y	0	43	0	43		Not estimable
2016 Shulan W	2	65	1	65	2.5%	2.03 [0.18, 22.98]
2016 Xiaojing D	0	47	0	49		Not estimable
2017 Ni Z	0	53	0	53		Not estimable
2017 Ping D	0	35	0	35		Not estimable
2018 Aimin Hu	0	50	0	50		Not estimable
2018 Min Z	0	36	1	36	3.8%	0.32 [0.01, 8.23]
2018 Shijian Q	0	76	0	74		Not estimable
2019 Changsong Z	2	55	2	55	5.0%	1.00 [0.14, 7.36]
2019 Chenhui D	0	39	0	39		Not estimable
2019 Suqin W	0	66	0	62		Not estimable
2019 Xiaoze S	0	47	0	47		Not estimable
2019 Zhixiong T	0	33	0	32		Not estimable
2020 Dandan P	0	55	0	55		Not estimable
2020 Xinxin P	0	36	0	34		Not estimable
2020 Yalan H	1	36	1	36	2.5%	1.00 [0.06, 16.63]
2021 Hongye C	3	72	6	72	14.8%	0.48 [0.11, 1.99]
2021 Huaizhi L	9	47	10	46	21.1%	0.85 [0.31, 2.34]
2021 Jinfeng S	0	40	0	40		Not estimable
2021 Ying L	6	49	8	49	18.1%	0.72 [0.23, 2.24]
2022 Li T	3	43	2	41	4.9%	1.46 [0.23, 9.23]
2022 Yan C	0	48	0	48		Not estimable
2023 Xue F	13	48	10	53	17.9%	1.60 [0.63, 4.08]
**Total (95% CI)**		**1329**		**1324**	**100.0%**	**0.94 [0.60, 1.48]**
Total events	42		45			

Heterogeneity: Chi² = 3.48, df = 9 (P = 0.94); I² = 0%
Test for overall effect: Z = 0.25 (P = 0.80)

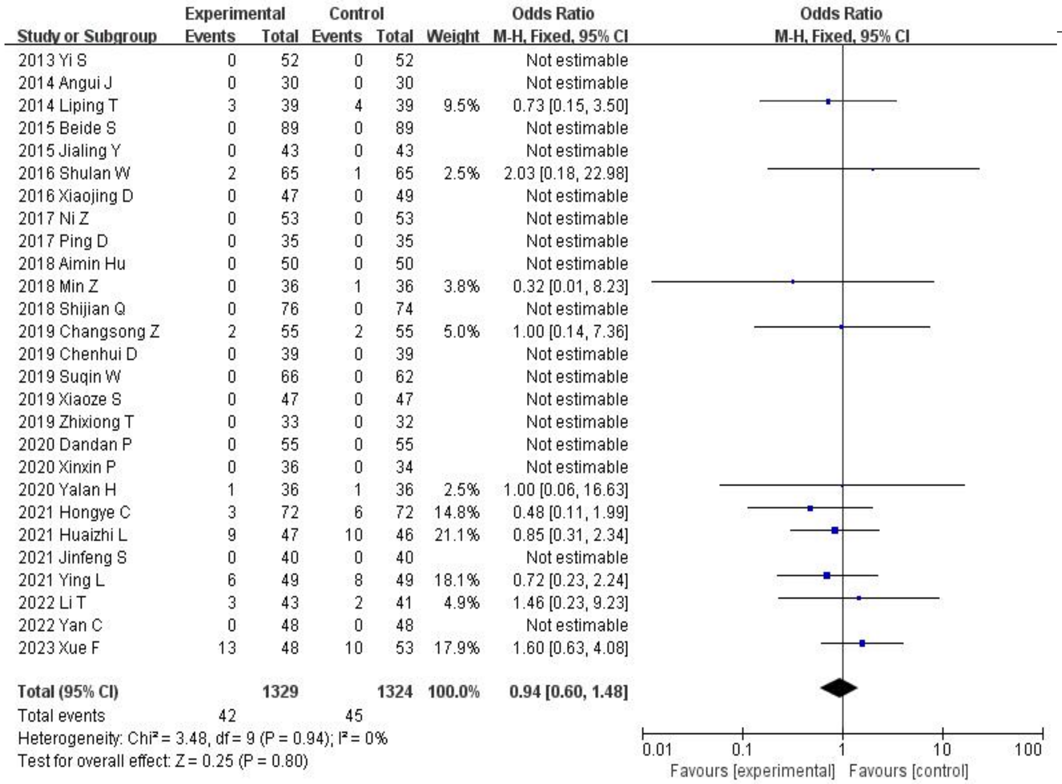

Study or Subgroup	Experimental Events	Experimental Total	Control Events	Control Total	Weight	Odds Ratio M-H, Fixed, 95% CI
**3.2.1 GC**						
2014 Angui J	0	52	0	52		Not estimable
**Subtotal (95% CI)**		**52**		**52**		**Not estimable**
Total events	0		0			
Heterogeneity: Not applicable						
Test for overall effect: Not applicable						
**3.2.2 YH**						
2018 Aimin Hu	0	50	0	50		Not estimable
**Subtotal (95% CI)**		**50**		**50**		**Not estimable**
Total events	0		0			
Heterogeneity: Not applicable						
Test for overall effect: Not applicable						
**3.2.3 JH**						
2021 Ying L	6	49	8	49	33.5%	0.72 [0.23, 2.24]
**Subtotal (95% CI)**		**49**		**49**	**33.5%**	**0.72 [0.23, 2.24]**
Total events	6		8			
Heterogeneity: Not applicable						
Test for overall effect: Z = 0.58 (P = 0.56)						
**3.2.4 BT**						
2019 Chenhui D	0	39	0	39		Not estimable
2021 Huaizhi L	9	47	10	46	39.0%	0.85 [0.31, 2.34]
**Subtotal (95% CI)**		**86**		**85**	**39.0%**	**0.85 [0.31, 2.34]**
Total events	9		10			
Heterogeneity: Not applicable						
Test for overall effect: Z = 0.31 (P = 0.76)						
**3.2.5 WJHT**						
2018 Shijian Q	0	76	0	74		Not estimable
2021 Hongye C	3	72	6	72	27.5%	0.48 [0.11, 1.99]
**Subtotal (95% CI)**		**148**		**146**	**27.5%**	**0.48 [0.11, 1.99]**
Total events	3		6			
Heterogeneity: Not applicable						
Test for overall effect: Z = 1.01 (P = 0.31)						
**3.2.6 YYT**						
2022 Yan C	0	48	0	48		Not estimable
**Subtotal (95% CI)**		**48**		**48**		**Not estimable**
Total events	0		0			
Heterogeneity: Not applicable						
Test for overall effect: Not applicable						
**3.2.7 GJ**						
2015 Jialing Y	0	53	0	53		Not estimable
**Subtotal (95% CI)**		**53**		**53**		**Not estimable**
Total events	0		0			
Heterogeneity: Not applicable						
Test for overall effect: Not applicable						
**3.2.8 HY**						
2015 Jialing Y	0	43	0	43		Not estimable
**Subtotal (95% CI)**		**43**		**43**		**Not estimable**
Total events	0		0			
Heterogeneity: Not applicable						
Test for overall effect: Not applicable						
**Total (95% CI)**		**529**		**526**	**100.0%**	**0.70 [0.36, 1.37]**
Total events	18		24			

Heterogeneity: Chi² = 0.42, df = 2 (P = 0.81); I² = 0%
Test for overall effect: Z = 1.04 (P = 0.30)
Test for subgroup differences: Chi² = 0.42, df = 2 (P = 0.81), I² = 0%

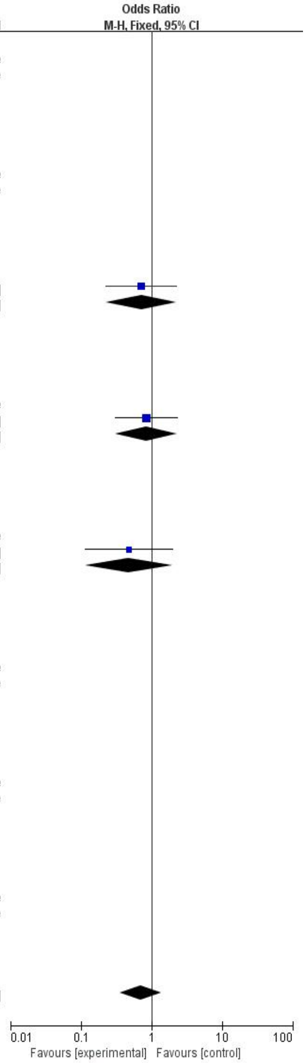

FIGURE 4 Adverse events forest plot& adverse events prescription forest plot

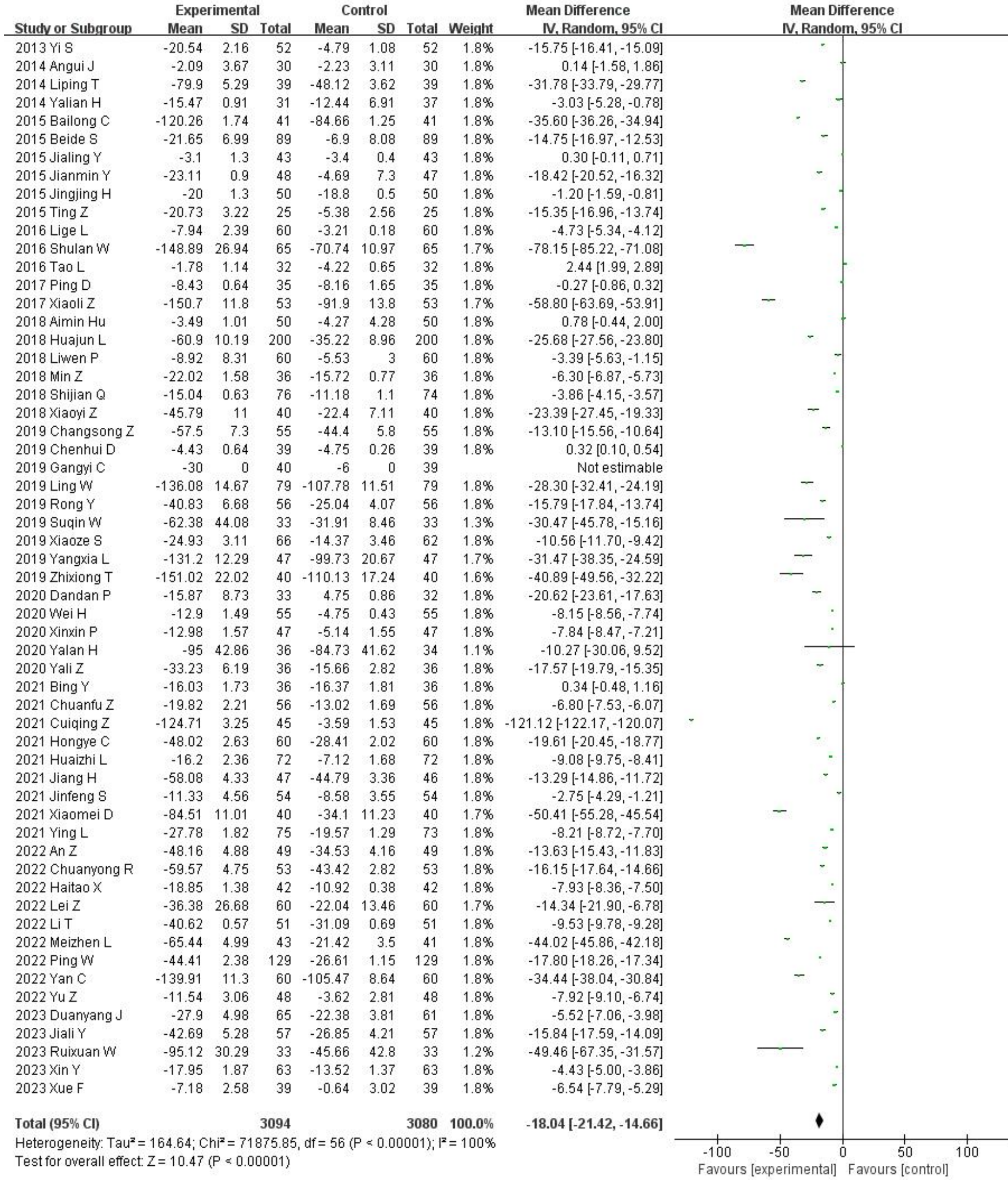

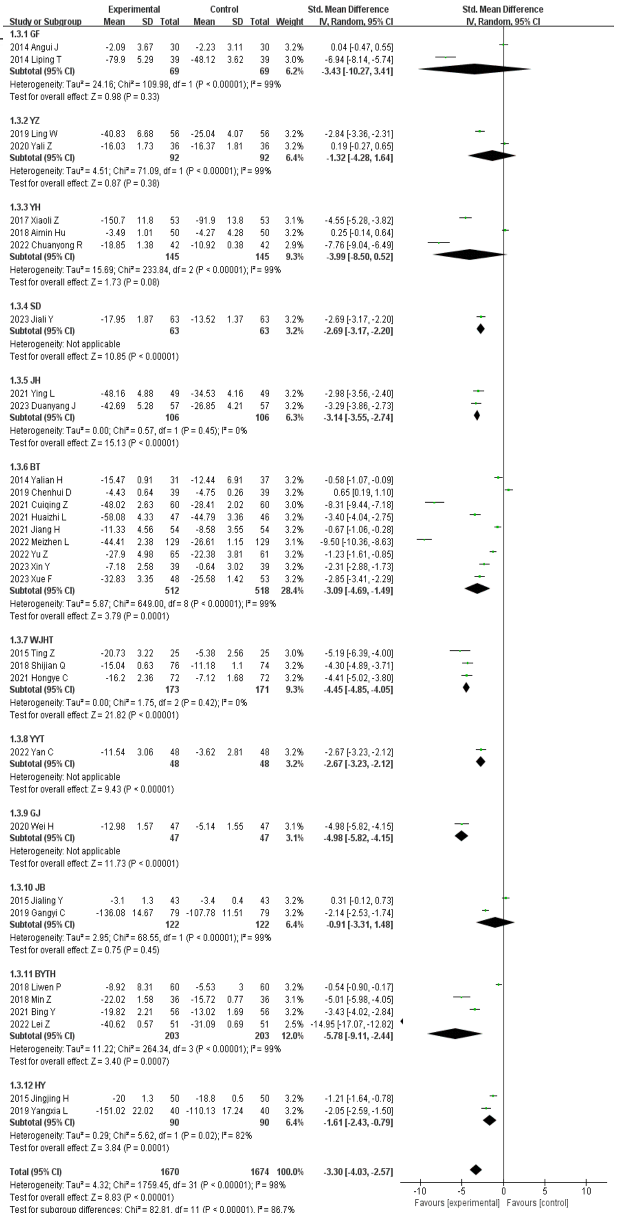

FIGURE 5 SCR forest plot & SCR prescription forest plot

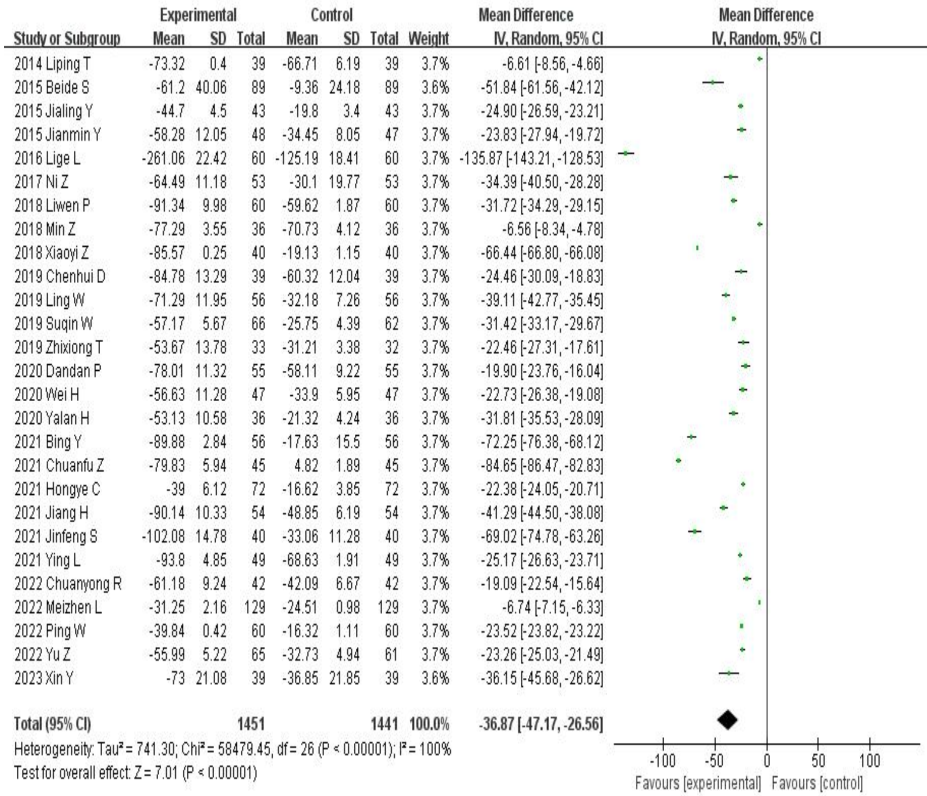

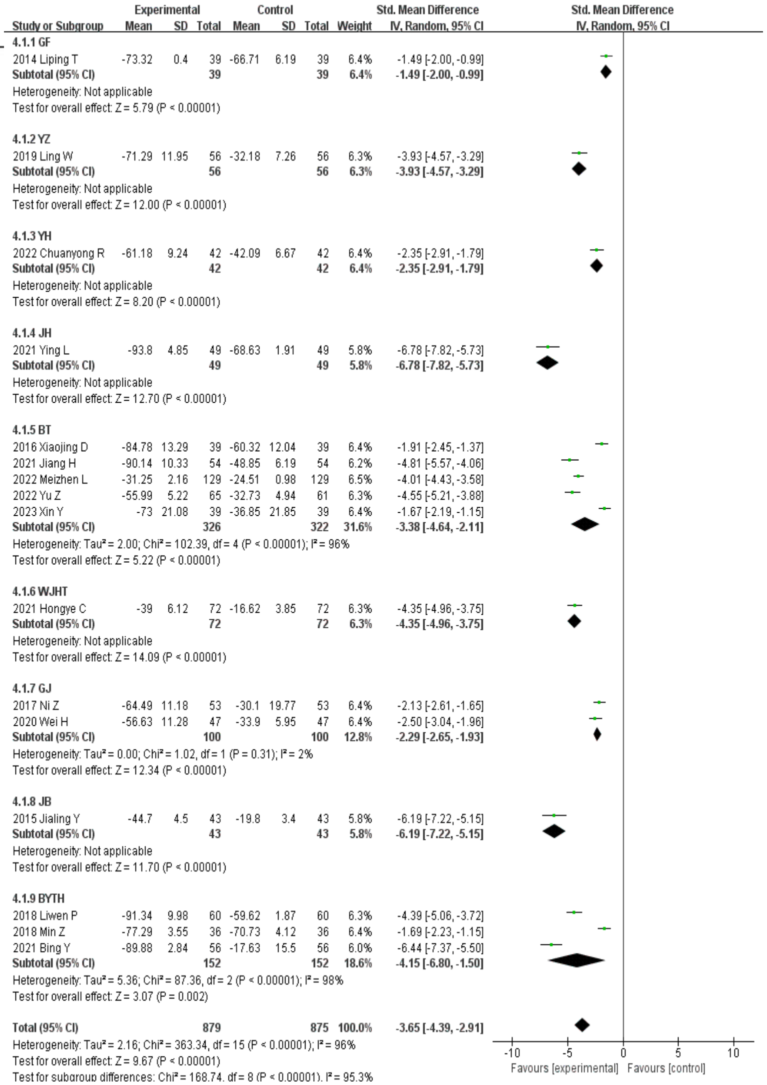

FIGURE 6 UAER forest plot & UAER prescription forest plot

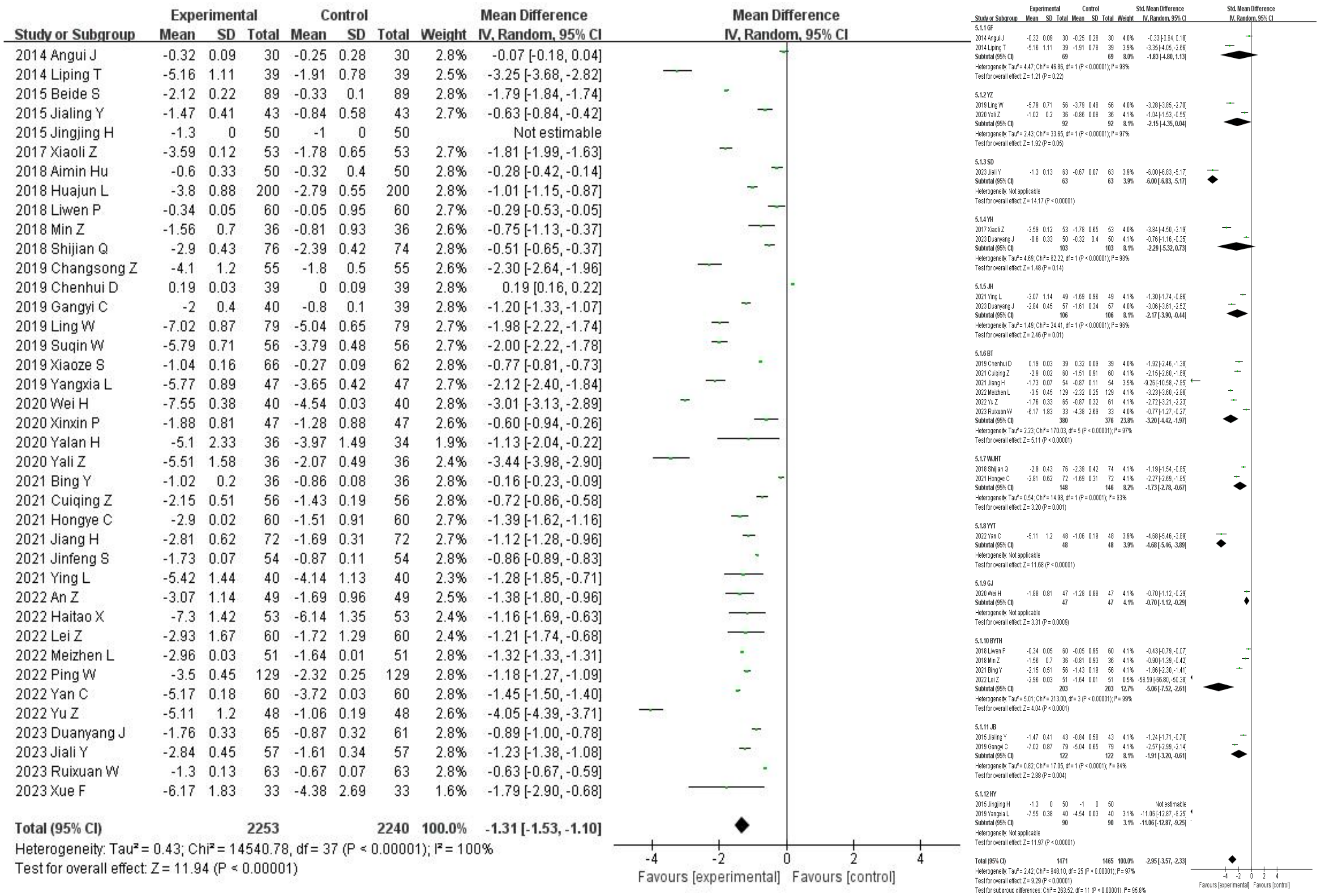

FIGURE 7 BUN forest plot & BUN prescription forest plot

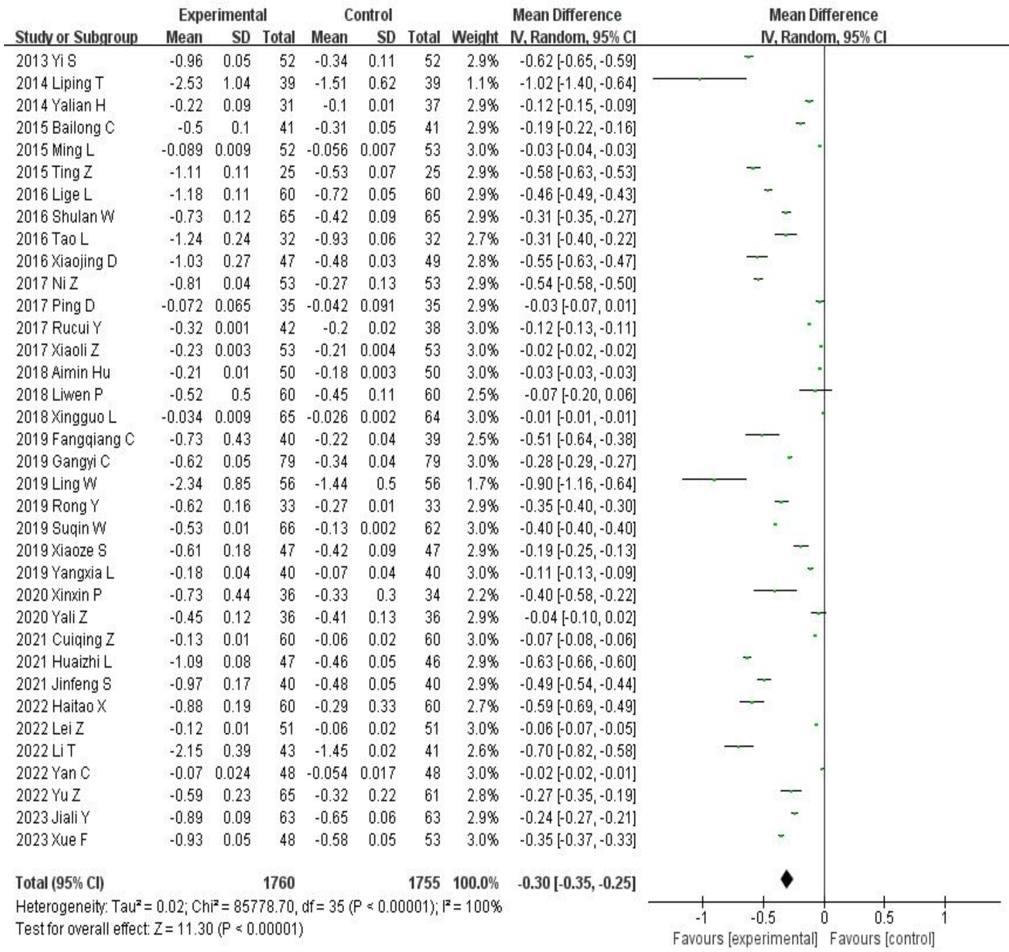

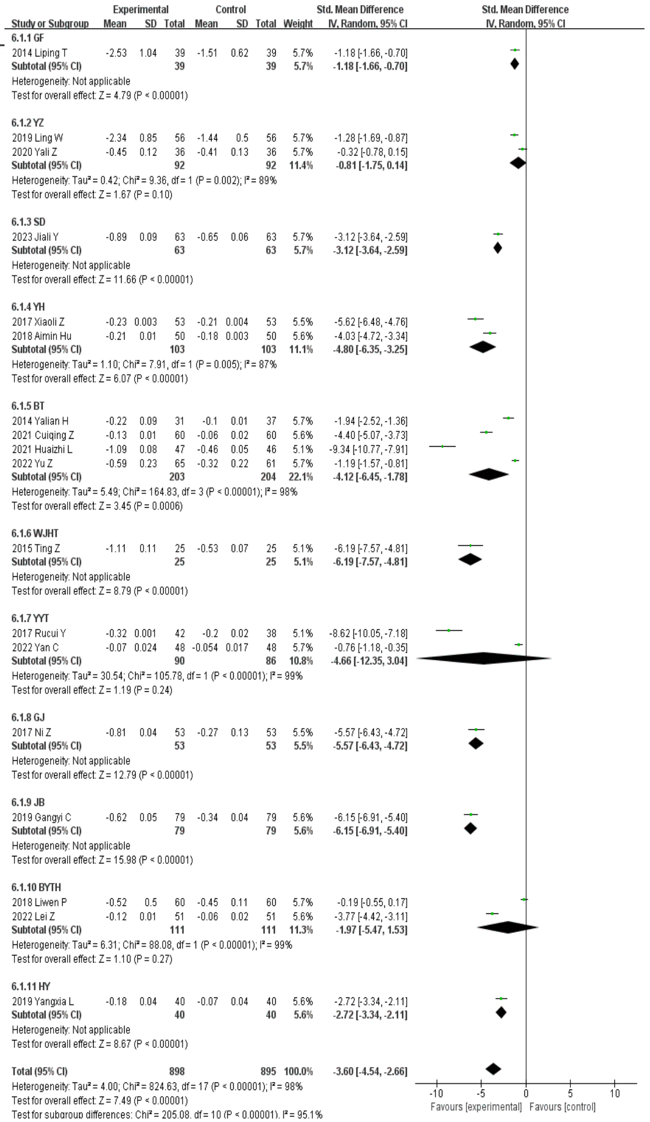

FIGURE 8 24h-utp forest plot & 24h-utp prescription forest plot

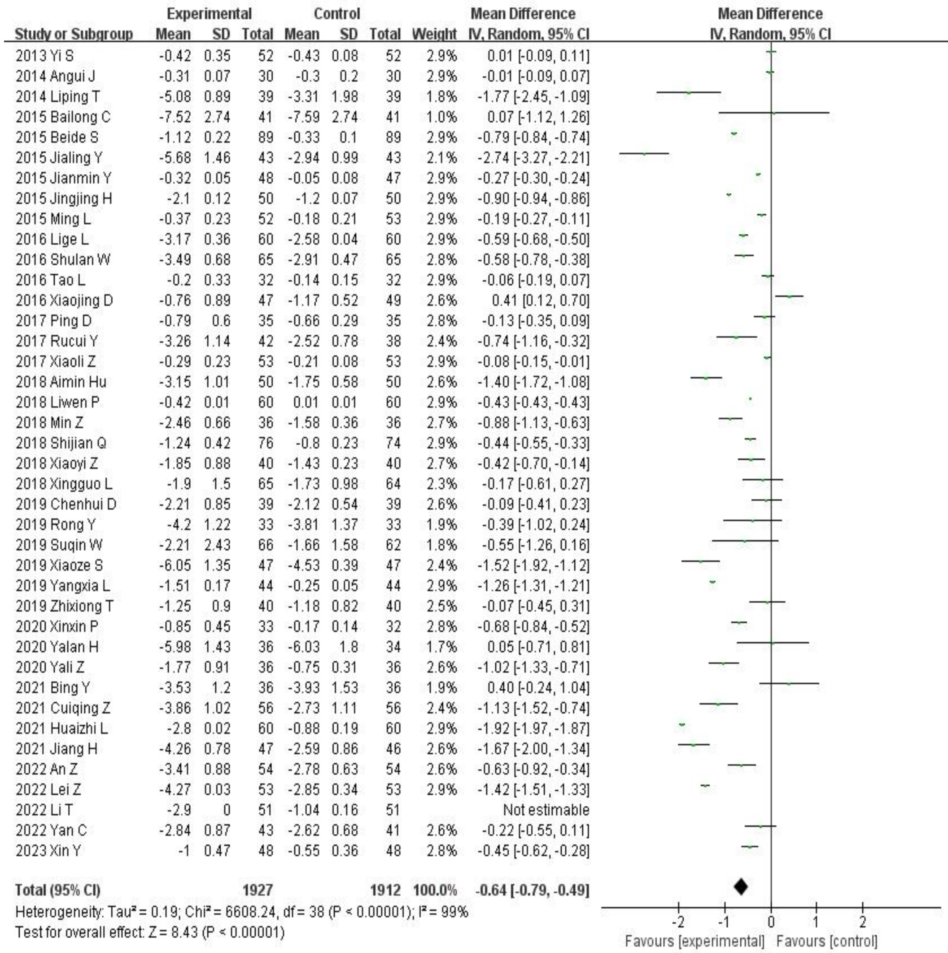

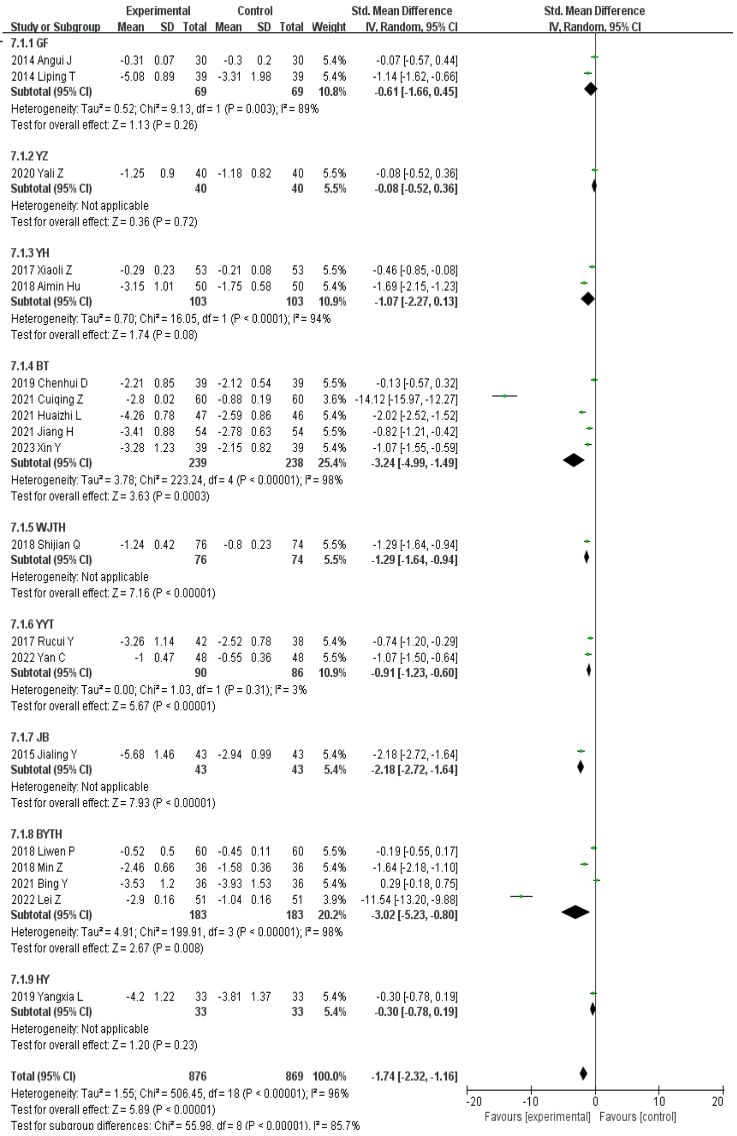

FIGURE 9 FPG forest plot & FPG prescription forest plot

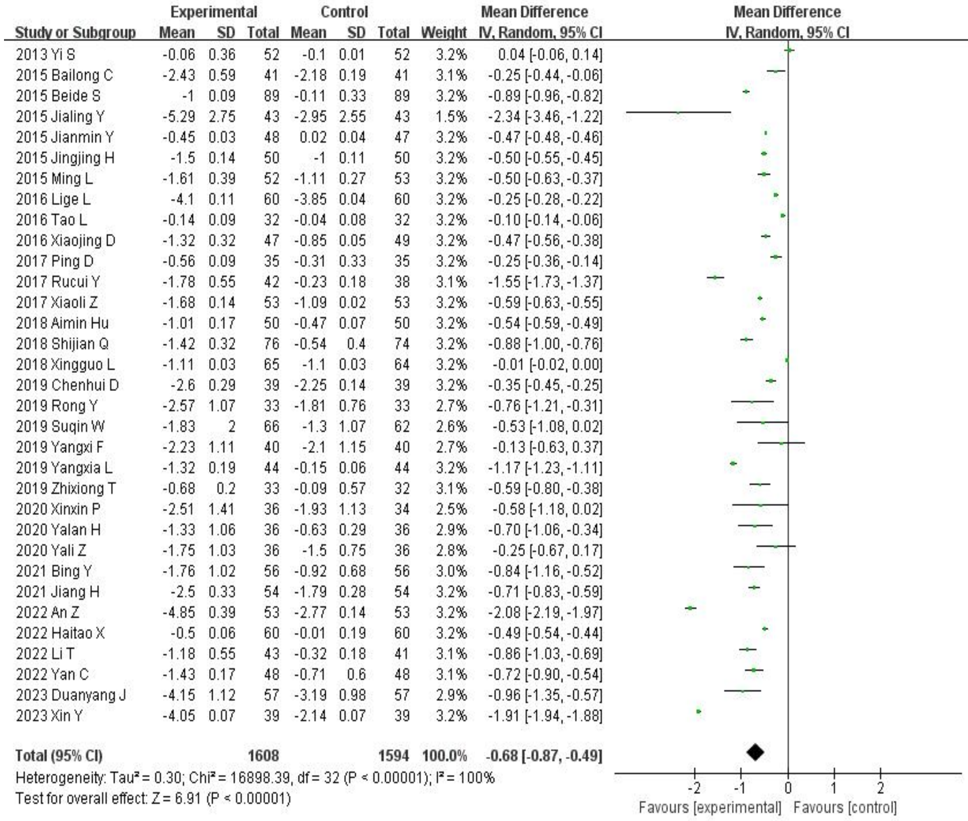

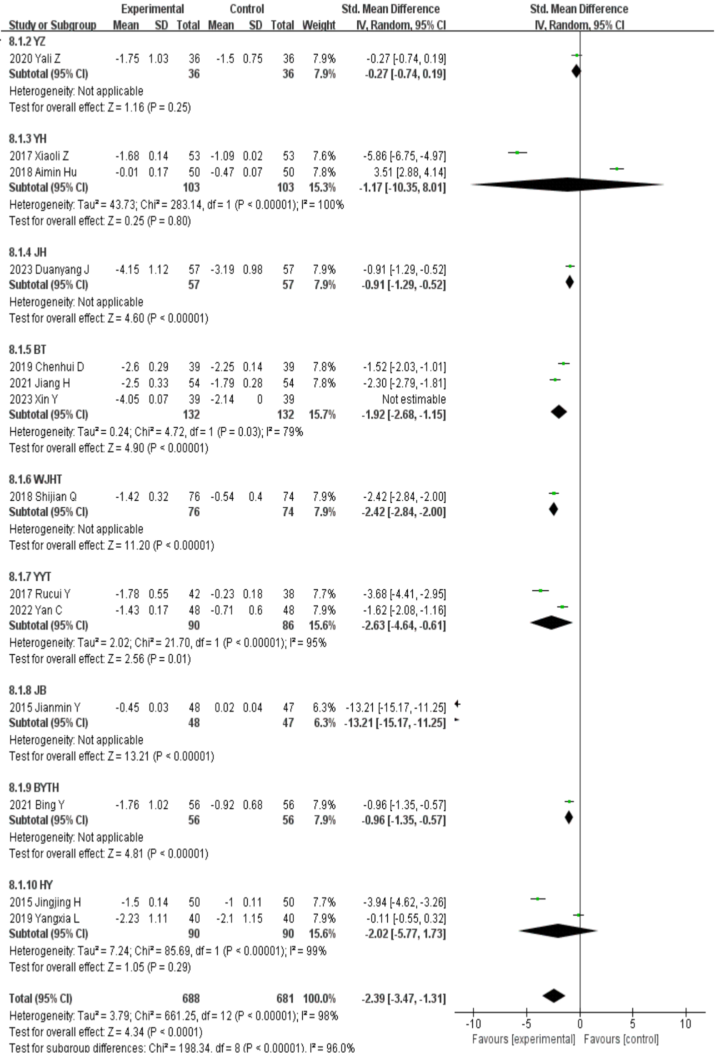

FIGURE 10 HbA1c forest plot & HbA1c prescription forest plot

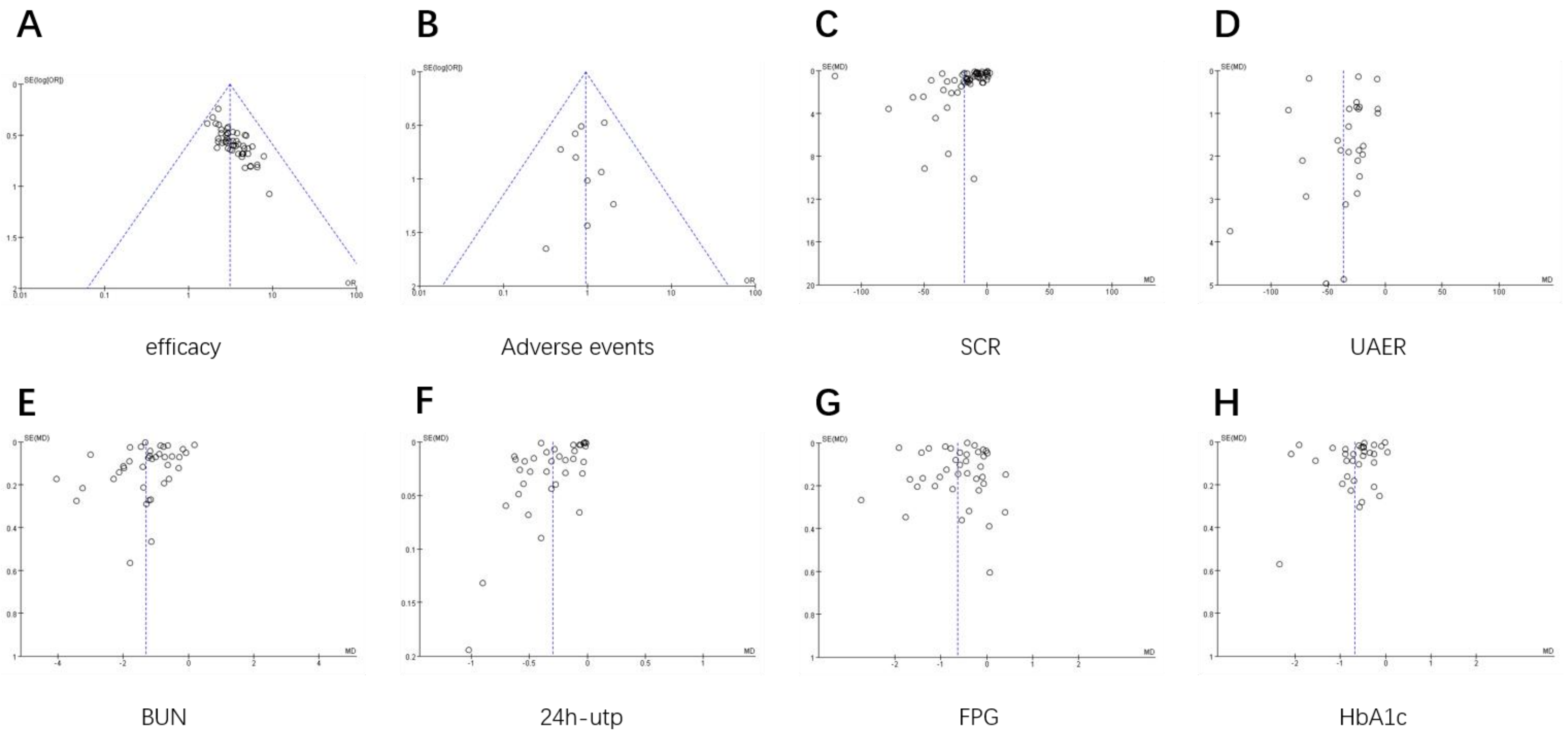

Fig. 11 Funnel plot

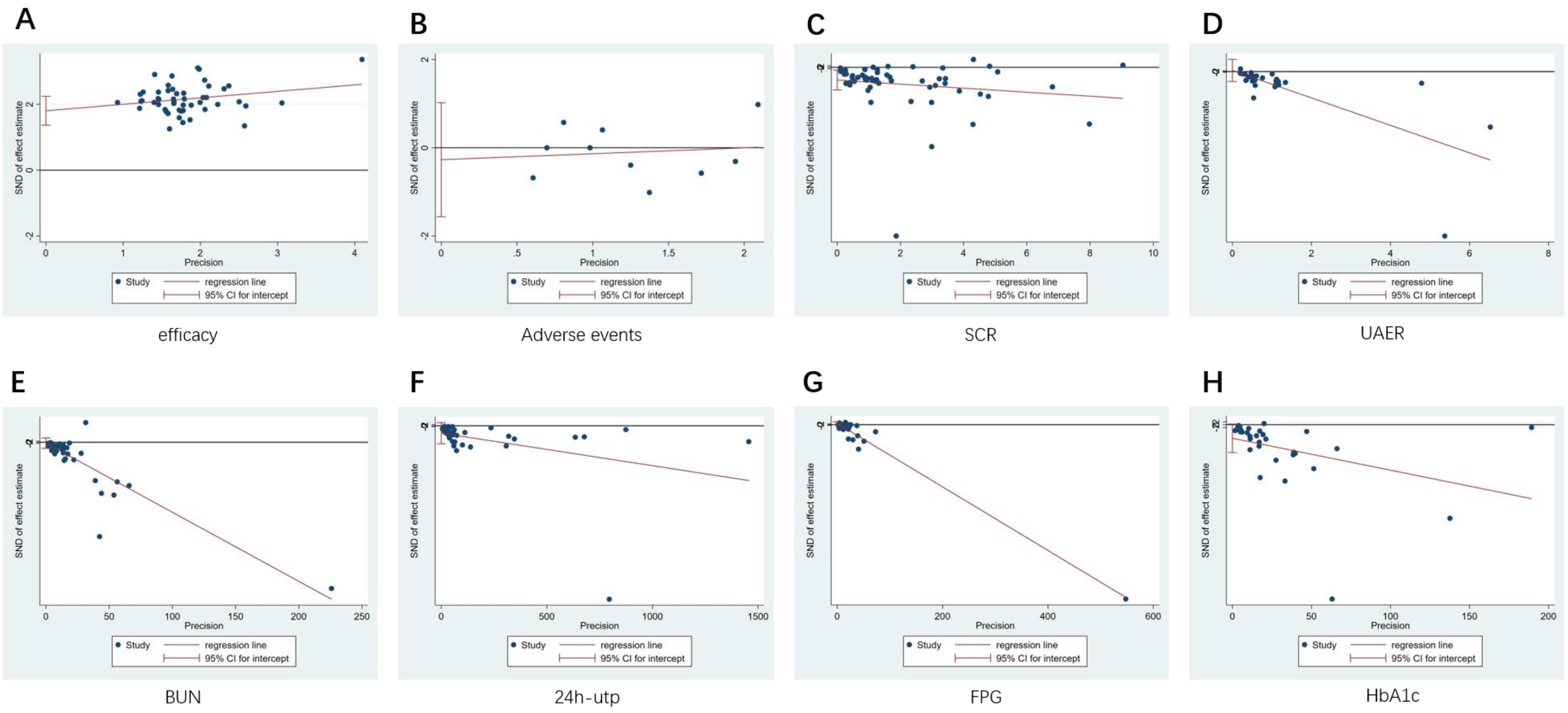

Fig. 12 Egger graph

ConPhyMP checklist of information for reporting plant material and its initial processing [1,2] (relevant for all studies on medicinal and foodplants including extract types A, B, and C)

SECTION/TOPIC	ITEM-NO.	CHECKLIST-ITEM	YES–NO–NOT-APPLICABLE	PAGE-NO., IF ANY
Title and abstract	1	A clear and concise title including an informative abstract and balanced summary.	✓ YES	page1
Description of the botanical drug and taxonomic authentication	2	Botanical or morphological authentication of the plant material (desirable is a combination with DNA barcoding, e.g., PCR, RFLP, genome sequencing) and the information must be included in a separate section of Material and Methods, if applicable, combined with the information required under item 3:	✓ NOT-APPLICABLE	
Description of the extract and extraction process	3	A separate section in Material and Methods, covers the relevant information on the material investigated, including the full species name(s), authorites and family; e.g. Salvia miltorrhiza Bunge [Lamiaceae; Salviaemiltorrhizae radix et rhizoma], and on the processing and extraction of the crude drug including the traditional processing of the material used medicinally (fumigation, steaming, roasting, cooking, frying, etc.).	✓ NOT-APPLICABLE	
Documentation of the legal basis for collection and processing	4	Full compliance with the Nagoya protocol, CITES, and all associated treaties including phytosanitary regulations.	✓ NOT-APPLICABLE	
Description of product characteristics, in case of a finished (commercial) product	5	Information on the characteristics of the commercial products including batch number and date of production/best by information and regulatory status.	✓ NOT-APPLICABLE	

Note: Please also include here the following information about your submitted manuscript:

Name of the journal:	Journal of Pharmacology
Date of the enquiry:	2023-12-22
Title of the manuscript:	Efficacy and Safety of Traditional Chinese Medicine Decocti
List of the authors:	Shyu Zheng1, Yunxi Xu1, Ya Zhang1*, Caiyi Long 2, Guo

Accessible at https://ga-online.org/conphymp

Society for Medicinal Plant and Natural Product Research

GA

[1] Please acknowledge/cite this as follows: Heinrich M, Jalil B, Abdel-TawabM, Echeverria J, Kulić Ž, McGaw LJ, et al. Best Practice in the chemical characterisation of extracts used in pharmacological and toxicological research—The ConPhyMP—Guidelines. Frontiers in Pharmacology. 2022;13:953205. https://doi.org/10.3389/fphar.2022.953205

[2] We strongly recommend reading this checklist in conjuncton with ConPhyMP 2022 explanaton and elaboraton for important clarifications on all items. If relevant, we also recommend after reading Heinrich et al. (2020) Best practice in research—Overcoming common challenges in phytopharmacological research. Journal of Ethnopharmacology. 2020;246:112230. https://doi.org/10.1016/j.jep.2019.112230

ConPhyMP checklist of items for conducting and reporting analytical methods[1,2] relevant for extract type A (for species or botanical drugs covered in a monograph in one of the national or regional pharmacopoeias)

SECTION/TOPIC	ITEM NO.	CHECKLIST ITEM	YES	NO	NOT APPLICABLE	PAGE NO., IF ANY
Type of extract	1	A – Confirm that the species or botanical drug under investigation is covered in a monograph in one of the national or regional pharmacopoeias.	✓			page 5
Preferred/main methods for extract characterisation/ chemical analysis	2	Compliance with pharmacopoeial standards to be followed:	✓			
		(a) The description of the active ingredients in the botanical drug (if known) or analytical marker compounds as defined.			✓	page 8
		(b) An analysis as defined in the monograph is needed if the extract has not been supplied with a certificate.			✓	
		(c) If the preparation was purchased, the manufacturer and certificate of analysis need to be included.				
		Including either the preferred or alternative approaches for characterisation:				
		(a) Triple chemical fingerprinting methods, each with one or more detection parameters.			✓	
		(b) Quantification of at least two marker compounds (unless this is not feasible, evidence needs to be provided), and justification of the choice of markers (if applicable).			✓	
Alternative methods for extract characterisation/chemical analysis	3	(a) Single chemical fingerprinting method with at least three different detection parameters (i.e., altered detection parameters, like TLC/ HPTLC with different staining reagents and/or UV excitation wave- lengths, HPLC-DAD/LCDAD with different wavelengths). The same applies to coupling MS or NMR to chromatographic techniques.			✓	
		(b) Quantification of at least two marker compounds (unless this is not feasible, evidence needs to be provided), and justification of the choice of markers (if applicable).			✓	
Use of reference standards	4	(a) Direct overlay of the chromatogram of the sample with that of an officially specified reference standard (if applicable).			✓	
		(b) Chromatographic fingerprinting: Direct overlay of the chromato- gram of the sample with that of official reference standards of the powdered plant material or the dry extract from the plant material.				
Comparison of different extracts/ samples of the same plants	5	(a) Direct comparison of the chromatographic/spectroscopic system and/or scoring system for "similarity" to be followed.			✓	

Note: Please also include here the following information about your submitted manuscript:

Name of the journal:	Journal of Pharmacology
Date of the enquiry:	2023-12-24
Title of the manuscript:	Efficacy and Safety of Traditional Chinese Medicine Decocti
List of the authors:	Shyu Zheng1, Yunxi Xu1, Ya Zhang1*, Caiyi Long2, Guo

[1] Please acknowledge/cite this as follows: Heinrich M, Jalil B, Abdel-TawabM, Echeverria J, Kulić Ž, McGaw LJ, et al. Best Practice in the chemical characterisation of extracts used in pharmacological and toxicological research—

The ConPhyMP—Guidelines. Frontiers in Pharmacology. 2022;13:953205. https://doi.org/10.3389/fphar.2022.953205

[2] We strongly recommend reading this checklist in conjuncton with ConPhyMP 2022 explanaton and elaboraton for important clarifications on all items. If relevant, we also recommend after reading Heinrich et al. (2020) Best practice in research—Overcoming common challenges in phytopharmacological research. Journal of Ethnopharmacology. 2020;246:112230. https://doi.org/10.1016/j.jep.2019.112230
